# Modeling mixoplankton along the biogeochemical gradient of the Southern North Sea

**DOI:** 10.1016/j.ecolmodel.2021.109690

**Published:** 2021-11-01

**Authors:** Lisa K. Schneider, Nathalie Gypens, Tineke A. Troost, Willem Stolte

**Affiliations:** a Deltares, Boussinesqweg 1, 2629 HV Delft, South-Holland, The Netherlands; b Université Libre de Bruxelles, Laboratoire d’Ecologie des Systèmes Aquatiques, CP221, Boulevard du Triomphe, B-1050, Belgium

**Keywords:** Mixoplankton, Trophic modes, Aquatic ecosystem model, North sea, Biogeochemical gradient, Delft3D-WAQ

## Abstract

The ecological importance of mixoplankton within marine protist communities is slowly being recognized. However, most aquatic ecosystem models do not include formulations to model a complete protist community consisting of phytoplankton, protozooplankton and mixoplankton. We introduce PROTIST, a new module for the aquatic ecosystem modelling software Delft3D-WAQ that can model a protist community consisting of two types of phytoplankton (diatoms and green algae), two types of mixoplankton (constitutive mixoplankton and non-constitutive mixoplankton) and protozooplankton. We employed PROTIST to further explore the hypothesis that the biogeochemical gradient of inorganic nutrient and suspended sediment concentrations drives the observed occurrence of constitutive mixoplankton in the Dutch Southern North Sea. To explore this hypothesis, we used 11 1D-vertical aquatic ecosystem models that mimic the abiotic conditions of 11 routine monitoring locations in the Dutch Southern North Sea. Our models result in plausible trophic compositions across the biogeochemical gradient as compared to in-situ data. A sensitivity analysis showed that the dissolved inorganic phosphate and silica concentrations drive the occurrence of constitutive mixoplankton in the Dutch Southern North Sea.

## Highlights

• A model to simulate the protist community including mixoplankton is introduced.
• The model was tested in 1D-V models along a biogeochemical gradient.
• Modelled mixoplankton are more sensitive to nutrients than to light.

## 1 Introduction

The trophic mode of marine protists is an important functional trait with which to characterize protists (Mitra et al., 2016). Flynn et al. (2019) classified marine protists into three categories based on their trophic mode: phytoplankton, protozooplankton and mixoplankton. Phytoplankton, such as diatoms and green algae (phototrophic non-diatoms), are defined as protists that can only utilize the photo-osmotrophic pathways. They cover their energy requirements through the photosynthetic fixation of inorganic carbon and their nutrient requirements through the uptake of dissolved inorganic nutrients. Protozooplankton are defined as protists that can only utilize the phagotrophic pathways. They cover their energy and nutrient requirements through the assimilation of prey.

In contrast to phytoplankton and protozooplankton, mixoplankton can utilize the photo-, osmo- and phagotrophic pathways simultaneously (Flynn et al., 2019). They can be divided into constitutive mixoplankton (CM) and non-constitutive mixoplankton (NCM) (Mitra et al., 2016). CMs have the constitutive ability to perform photosynthesis and they can uptake dissolved inorganic nutrients as well as assimilate prey. NCMs need to acquire the photosynthetic machinery from their prey. They cover their nutrient requirements mainly through the assimilation of prey (Stoecker et al., 2017).

The composition and productivity of protist communities is an interplay between external resource availability (such as nutrients, light and prey) and the protists’ physiologies. While the importance of phytoplankton (as the base of marine ecosystems) and protozooplankton (as trophic transfers) has long been recognized, the ecological relevance of mixoplankton has long been ignored (Flynn et al., 2013). However, mixoplankton contribute notably to marine protist communities worldwide (Leles et al., 2017, Leles et al., 2018, Faure et al., 2019), change the inorganic nutrient and predation dynamics (Hansen et al., 2019) and have a non-negligible impact on the carbon cycle (Worden et al., 2015). Furthermore, mixoplankton are an important connector between microbial, protist and mesozooplankton food webs (Stoecker et al., 2017) and they play an important role in ecosystems governed by strong light and nutrient gradients (Selosse et al., 2017).

In a recent analysis of routine monitoring data on the protist community of the Dutch Southern North Sea (from here on referred to as the Southern North Sea - SNS), Schneider et al. (2020) showed that CMs occur mostly in inorganic nutrient depleted, highly transparent, stratified environments. Eutrophied, turbid, mixed environments were devoid of CMs. Schneider et al. (2020) hypothesized that the environmental factors which exhibit a biogeochemical gradient drive the trophic composition of protist communities in the SNS. However, Schneider et al. (2020) were not able to elucidate which environmental factor – the dissolved inorganic nutrient or the suspended sediment gradient – governed the occurrence of CMs.

**Fig. 1:**
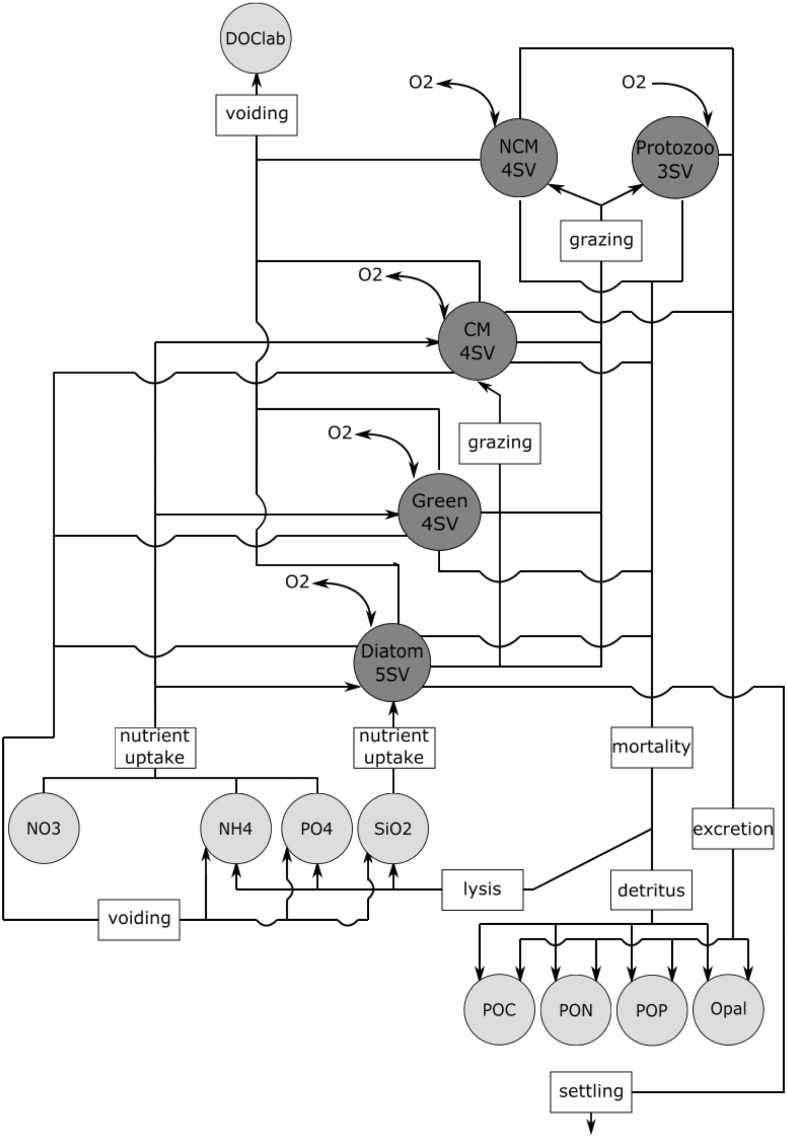
Conceptual visualization of the module PROTIST. The lightgrey circles are abiotic state variables (SV) and the darkgrey circles the protist functional types (PFT). Each PFT consists of multiple SVs. The arrows and the labels depict the interaction between the PFTs as well as the interactions between the abiotic SVs and the PFTs. DOClab stands for labile dissolved organic carbon.

While routine monitoring data allows us to gain insight on protist communities in different environments, it is difficult to test causalities between separate abiotic factors and the community composition using field data. Aquatic ecosystem models (AEM) provide a tool to estimate the impact of external or internal forcing on aquatic ecosystem dynamics (i.e. Janssen et al., 2015). AEMs are able to depict various aquatic processes such as primary production, secondary grazing, remineralization or denitrification. However, most AEMs still do not include adequate formulations for mixoplankton (Flynn et al., 2019).

To help close this gap, we implemented a new module that includes formulations to model a protist community consisting of diatoms, green algae, protozooplankton, CMs and NCMs in the open-source AEM Delft3D-WAQ. This new module is called PROTIST and can be used to model the primary production of as well as the competition and grazing within the protist community.

We validated PROTIST using 11 1D-vertical (1D-V) models that mimic 11 routine monitoring location classes located in the estuaries, coasts and offshore regions of the Dutch SNS (Schneider et al., 2020). The 11 1D-V models were forced with timeseries of inorganic nutrients and suspended sediments sampled at the 11 location classes. Thus, we were able to quantitatively and qualitatively validate PROTIST across the biogeochemical gradient of the SNS using timeseries comparisons, target diagrams and heatmaps.

Using the 11 1D-V models, we were also able to further explore the hypothesis put forward by Schneider et al. (2020) that the biogeochemical gradient drives the trophic composition of protist communities. We conducted a sensitivity analysis to determine whether the inorganic nutrients or the suspended sediment concentration has more effect on the occurrence of CMs. Especially against the background of climate change (Wilken et al., 2013) and anthropogenic changes to marine environments (Burkhard et al., 2011), it is important to acquire more knowledge about the abiotic drivers of protist community compositions.

In summary, the objectives of this study are (1) to introduce the module PROTIST, (2) to quantitatively and qualitatively validate PROTIST against routine monitoring data and (3) to further explore the hypothesis that the biogeochemical gradient drives the trophic composition of protist communities.

## 2 Material and methods

### 2.1 The module PROTIST

The module PROTIST simulates the growth and mortality of the protist community while taking the trophic modes of protists into account. PROTIST can thus be used to simulate primary production in marine ecosystems. The module PROTIST combines model equations from Flynn, 2001, Flynn and Mitra, 2009 and Flynn (2021). These equations are based on first principles that were implemented unchanged in PROTIST. Firstly, the growth of protists is not only determined by the external availability of resources such as light, nutrients and prey, but also by the protists’ internal stoichiometry. The internal nutrient quotas of the protists regulate the protists’ affinity to uptake nutrients (Grover, 1991), synthesize chlorophyll-a (Davey et al., 2008) and assimilate prey (Mitra and Flynn, 2005). Secondly, the trophic modes of protists determine the interactions within protist communities (Flynn et al., 2019). However, some changes were needed for the equations to run stably and efficiently as a Delft3D-WAQ module:

• the nutrient uptake equations were described using continuous functions (instead of coupled conditional statements as in Flynn, 2021).
• the uptake of dissolved amino acids was not implemented, as Delft3D-WAQ does not simulate dissolved amino acids explicitly due to the lack of validation data.
• the assimilation of dissolved organic carbon was not implemented, as all protist functional types (PFT) can assimilate dissolved organic carbon (DOC) (Stoecker et al., 2017), so it is not a distinguishing pathway between the PFTs.
• PROTIST enables multiple PFTs to interact with, compete against and graze on each other.

PROTIST consists of five different PFTs and each PFT consists of state variables (SV) that describe carbon (C), nitrogen (N) and phosphorus (P) biomass. Chlorophyll-a (Chl) is an additional SV for phototrophic protists. Diatoms contain an additional SV to describe the silica (Si) content. This makes PROTIST fully stoichiometrically variable. The PFTs require either light and/or prey and/or nutrients. Fig. 1 visualizes the SVs required for PROTIST and the interactions between the different SVs.

### 2.2 Functional types in PROTIST

#### 2.2.1 Diatoms

The PFT diatoms are defined as phytoplankton that can utilize silica. Diatoms are described with five SVs: $Diat_{C}$, $Diat_{N}$, $Diat_{P}$, $Diat_{\mathrm{Si}}$ and $Diat_{Chl}$. Table A.6 provides the conservation equations for the diatom SVs. The diatom SVs increase over time through the uptake of nutrients ($up_{\mathrm{NH}4}$, $up_{\mathrm{NO}3}$, $up_{\mathrm{PO}4}$, $up_{\mathrm{Si}}$), the fixation of carbon (Cfix) and the synthesis of chlorophyll-a (synChl). The diatom SVs decrease over time through predation (Pred), mortality (mrt), the leakage of photosynthate (Cleak), the voiding of excess nutrients or carbon (Nout, Pout, Cvoid), the degradation of chlorophyll-a (degChl) and respiration (totR).

#### 2.2.2 Green algae

The PFT green algae are defined as phytoplankton that cannot utilize silica. Green algae are described with four SVs: $Green_{C}$, $Green_{N}$, $Green_{P}$ and $Green_{Chl}$. Table A.7 provides the conservation equations for the green algae SVs. The green algae SVs increase over time through the uptake of nutrients ($up_{\mathrm{NH}4}$, $up_{\mathrm{NO}3}$, $up_{\mathrm{PO}4}$), the fixation of carbon (Cfix) and the synthesis of chlorophyll-a (synChl). The green algae SVs decrease over time through predation (Pred), mortality (mrt), the leakage of photosynthate (Cleak), the voiding of excess nutrients or carbon (Nout, Pout, Cvoid), the degradation of chlorophyll-a (degChl) and respiration (totR).

#### 2.2.3 Protozooplankton

The PFT protozooplankton are defined as protists that are only capable of phagotrophy. Protozooplankton are described using three SVs: $Zoo_{C}$, $Zoo_{N}$ and $Zoo_{P}$. Table A.8 provides the conservation equations for the protozooplankton SVs. The protozooplankton SVs increase over time through the assimilation of prey (assC, assN, assP). The protozooplankton SVs decrease over time through mortality (mrt - includes implicit grazing by higher trophic levels through use of a quadratic closure function), the voiding of unassimilated prey (POCout, PONout, POPout) and respiration (totR).

#### 2.2.4 Constitutive mixoplankton

The PFT CM are defined as mixoplankton that are primarily phototrophic, but are also capable of phagotrophy. CMs require four SVs: $CM_{C}$, $CM_{N}$, $CM_{P}$ and $CM_{Chl}$. Table A.9 provides the conservation equations for the CM SVs. The CM SVs increase over time through the uptake of nutrients ($up_{\mathrm{NH}4}$, $up_{\mathrm{NO}3}$, $up_{\mathrm{PO}4}$), the fixation of carbon (Cfix), the synthesis of chlorophyll-a (synChl) and the assimilation of prey (assC, assN, assP). The CM SVs decrease over time through predation (Pred), mortality (mrt), the leakage of photosynthate (Cleak), the voiding of excess nutrients or carbon (Nout, Pout, Cvoid), the voiding of unassimilated prey (POCout, PONout, POPout), the degradation of chlorophyll-a (degChl) and respiration (totR).

#### 2.2.5 Non-constitutive mixoplankton

The PFT NCM are defined as mixoplankton that are primarily phagotrophic, but are also capable of enslaving the photosynthetic machinery of their phototrophic prey. NCMs require 4 SVs: $NCM_{C}$, $NCM_{N}$, $NCM_{P}$ and $NCM_{Chl}$. Table A.10 provides the conservation equations for the NCM SVs. While NCMs have been shown to also uptake inorganic nutrients, the percentage of uptake is negligible compared to the acquisition of nutrients from prey (Schoener and McManus, 2017). The NCM SVs increase over time through the assimilation of prey (assC, assN, assP), the uptake of chloroplasts (upChl) and the fixation of carbon (Cfix). The NCM SVs decrease over time through predation (Pred), mortality (mrt), the leakage of photosynthate (Cleak), the voiding of unassimilated prey (POCout, PONout, POPout), the loss of chlorophyll-a (lossChl) and respiration (totR).

### 2.3 Physiological processes in PROTIST

The following sections describe the mathematical formulations needed to compute the physiological processes of the different PFTs. The equations are listed in Appendix A.6.

**Fig. 2:**
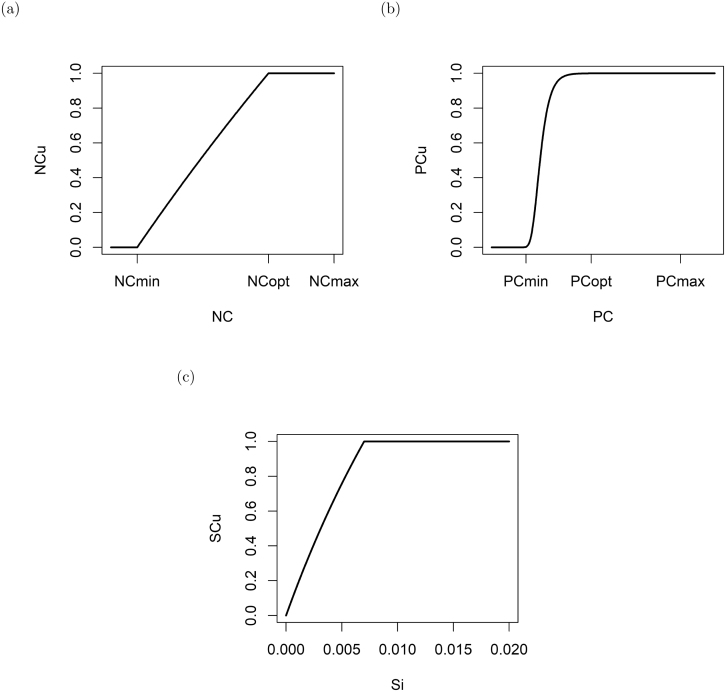
Visualization of the internal nutrient status for (a) nitrogen NCu [dl], (b) phosphate PCu [dl] and (c) silica SCu [dl]. A value of 1 denotes that the internal nutrient stores are optimal, a value of 0 that the internal nutrient stores are completely depleted. The figures display that while NCu decreases linearly as soon as the optimal quota is not reached, PCu does not. These functions mathematically describe that nitrogen cannot be stored within the cell, while phosphate as polyphosphate can.

Modified from Flynn (2021).

**Fig. 3:**
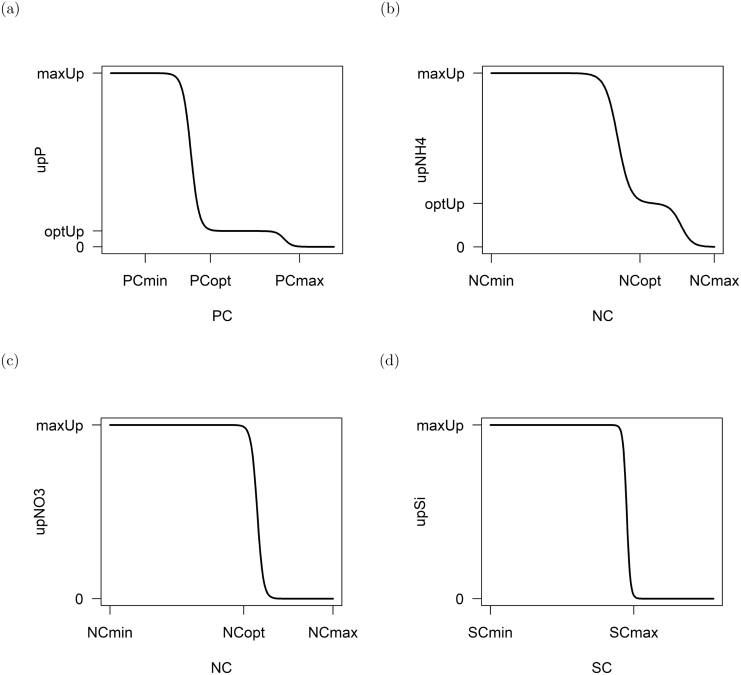
Visualization of the nutrient uptake for (a) phosphate (upP [gP gC^−1^ d^−1^]), (b) ammonium (upNH4 [gN gC^−1^ d^−1^]), (c) nitrate (upNO3 [gN gC^−1^ d^−1^]) and (d) silica (upSi [gSi gC^−1^ d^−1^]). The figures display that the uptake of phosphate and ammonium is repressed once the optimum cellular status is reached, while the uptake of nitrate and silica is stopped all together after the optimum quota is passed.

Modified from Flynn (2021).

**Fig. 4:**
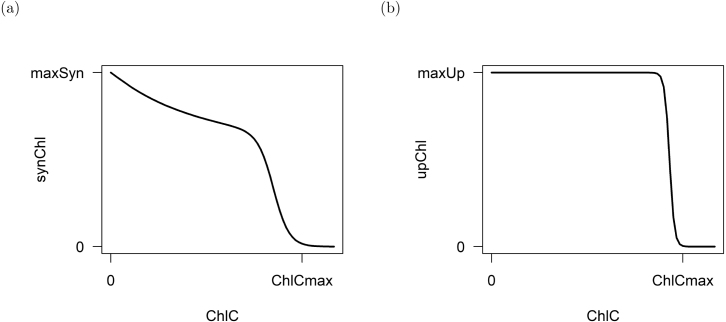
Visualization of the (a) synthesis of chlorophyll-a for diatoms, green algae and CMs (synChl)and (b) the uptake of chloroplasts by NCMs from their prey (upChl). The figures display that the synthesis of chlorophyll-a is repressed depending on the amount of carbon fixed and that NCMs can uptake chloroplasts until a maximum chlorophyll-a:carbon is reached.

**Fig. 5:**
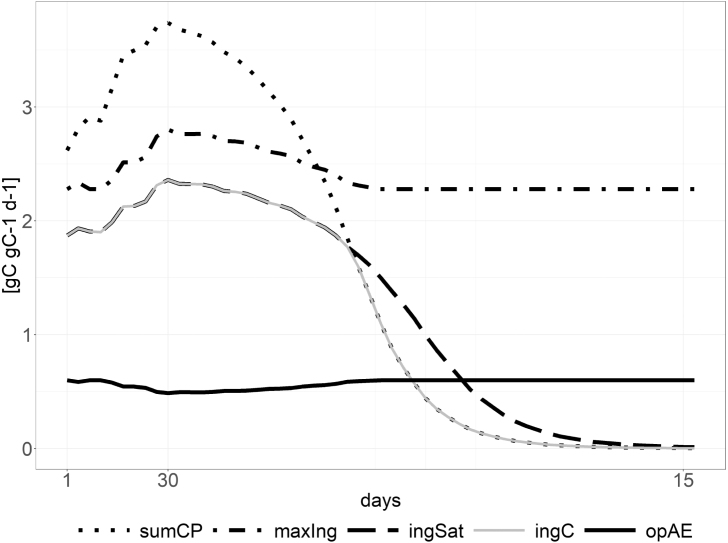
Visualization of the ingestion rate. The ingestion increases with decreasing prey quality (opAE - solid, black line), while the actual ingestion (ingC - solid, grey line) is limited either by the satiation rate (ingSat - dashed, black line) or by the amount of captured prey (sumCP - dotted, black line).

**Fig. 6:**
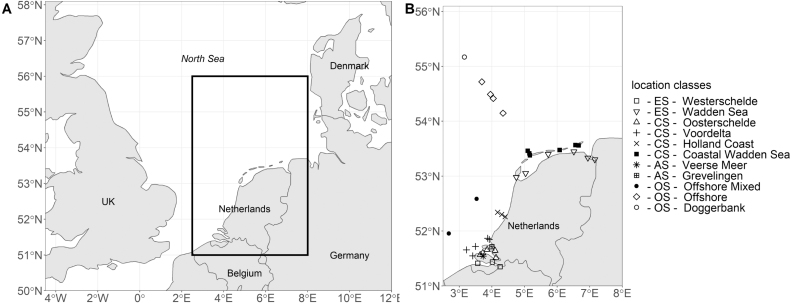
Geographic location of the location classes (A - overview and B - detailed) that are simulated with the 1D-V models.

**Fig. 7:**
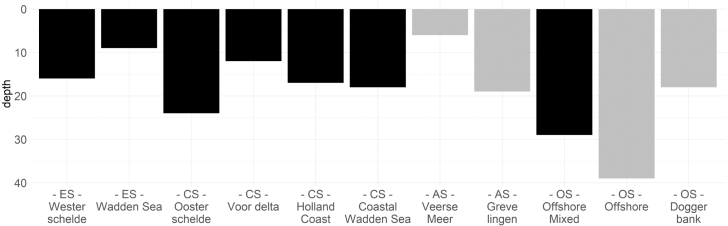
Depth of the 1D-V models. The grey colour highlights that stratification was applied to those 1D-V models.

**Fig. 8:**
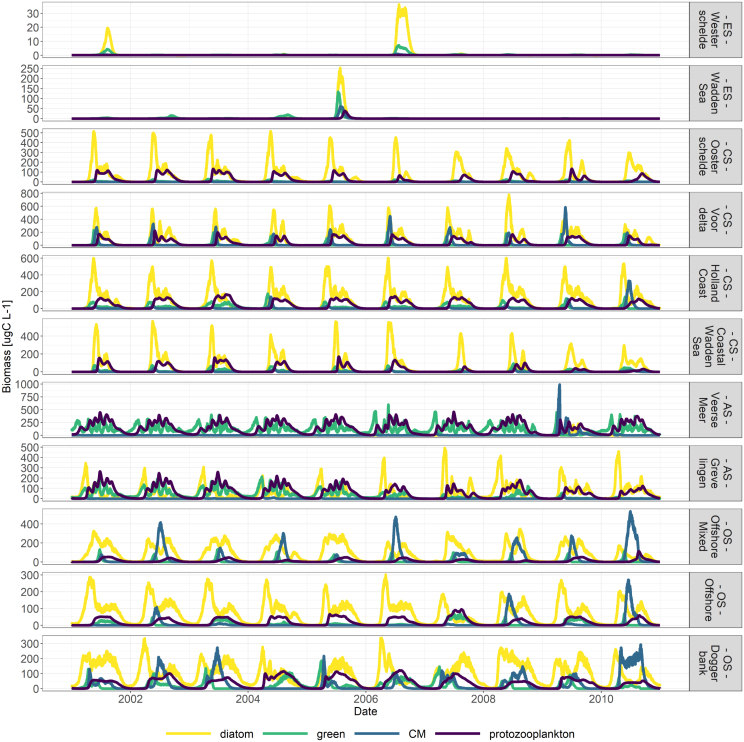
Timeseries of protist carbon SVs for the four different PFTs. The colour yellow depicts diatoms, the colour green the green algae, light blue the CMs and dark blue the protozooplankton. The timeseries clearly show different orders of magnitude from CS to OS as well as year-to-year variations. Please note the differences in the y-axes.

#### 2.3.1 Cellular status

For each PFT the cellular quota, the maximum growth rate, the mortality rate, the basal respiration rate, the total respiration rate, the carbon-specific growth rate as well as the cellular nutrient status of nitrogen, phosphate and silica needs to be calculated. Table A.12 summarizes the description of the auxiliaries and Appendix A.6.2 provides the detailed mathematical equations.

The cellular quotas (NC, PC, SC) describe the ratio of the respective protist nutrient SVs to the protist carbon SVs according to Droop (1974). The maximum growth rate (UmT) as well as the mortality rate (mrt) are calculated using the Q10 approach (Van’t Hoff, 1884). The basal respiration rate (BR) is defined as a fraction of maximum growth rate (Geider and Osborne, 1989). The total respiration is the sum of the metabolic cost (redco) of nitrate reduction (upNO3) (Flynn and Flynn, 1998), the anabolic cost (AR) of nitrogen utilization (upNH4, assN) (Wirtz and Pahlow, 2010), the foraging costs for prey (SDA, assC) (Pahlow and Prowe, 2010) and the basal respiration (BR) (Geider and Osborne, 1989).

Furthermore, the nutrient status for nitrogen (NCu), phosphate (PCu) and silica (SCu - only for diatoms) is calculated. The nutrient status returns values between 0 (severely deprived of the respective nutrient) and 1 (at the optimal nutrient quota). The form of the functions depends on the protist’s physiology to store the respective nutrient (see Fig. 2). As protist cells store nitrogen in a form that is not physiologically active (Andersen et al., 1991), the nutrient status for nitrogen (NCu - see Fig. 2(a)) is a linear function between the minimum and maximum quota. The nutrient status for phosphate (PCu - see Fig. 2(b)) is calculated using a sigmoidal function to mimic the storage of phosphate as polyphosphate within the cell (Lin et al., 2016). The cellular status of silica (SCu - see Fig. 2(c)) is a function of the external silica availability, as incorporated silica is not accessible by the cells anymore (Martin-Jézéquel et al., 2003). Applying Liebig’s law of minimum (Liebig, 1840), the limiting nutrient (NPCu or NPSiCu - for diatoms) is determined by the minimum nutrient status within the cell.

To ensure that the nitrogen:carbon and phosphate:carbon quotas do not exceed the maximum nutrient quota between time steps, cellular nitrogen and phosphate is voided as soon as the cellular nutrient quota exceeds the maximum nutrient quota (Nout and Pout). This does not occur for silica, as incorporated silica cannot be dispelled from the cell walls of diatoms (Martin-Jézéquel et al., 2003). If the nitrogen:carbon quota falls below the minimum nitrogen:carbon quota, then carbon is voided (Cvoid).

#### 2.3.2 Uptake

In general, the uptake of dissolved inorganic nutrients(upNH4, upNO3, upP, upSi) is a combination of the external availability of the nutrients and the acquisition capability, which depends on the internal nutrient status (Grover, 1991, Moreno and Martiny, 2018). This is achieved by enhancing or repressing the optimal nutrient uptake via sigmoidal functions (see Fig. 3). Table A.13 summarizes the description of the auxiliaries and Appendix A.6.3 provides the detailed mathematical equations.

The nutrient uptake at the optimal nutrient quota is regulated via the Michaelis–Menten function and scaled to the maximum growth rate and the optimal nutrient:carbon quota. For the uptake of NH${}_{4}^{+}$ and NO${}_{3}^{-}$, the optimum nutrient uptake is also scaled to the relative growth feasible with the respective nutrient. If the cellular nutrient quota is below the optimum nutrient quota (i.e. nutrient stressed), the nutrient uptake is enhanced until the maximum nutrient uptake is reached (Goldman and Glibert, 1982, Perry, 1976). If the cellular nutrient quota is above the optimum nutrient quota, the nutrient uptake for NH${}_{4}^{+}$ and PO${}_{4}^{3-}$ are repressed (Wirtz and Pahlow, 2010), while the nutrient uptake for NO${}_{3}^{-}$ (Dugdale et al., 2007, Domingues et al., 2011) and silica are stopped all together. Furthermore, the uptake of nitrogen is a function of the cellular phosphate:carbon quota resulting in a decrease of the cellular nitrogen:carbon quota during phosphate stress (Pahlow and Oschlies, 2009).

#### 2.3.3 Phototrophy

The photosynthesis equations are based on the photosynthesis-irradiance curve that requires three input parameters: the maximum photosynthetic rate (PSqm), the chlorophyll-a specific initial slope ($\alpha^{Chl}$) and photon flux density (PFD) (Jassby and Platt, 1976). Table A.14 summarizes the description of the auxiliaries and Appendix A.6.4 provides the detailed mathematical equations.

The maximum rate of photosynthesis covers the basal respiration (BR), the maximum growth rate (UmT), the leakage of photosynthate as DOC (PSDOC) (Thornton, 2014) and the costs of reducing nitrate (redco and AR) (Dugdale et al., 2007) which (with the exception of basal respiration) are all influenced by the nitrogen quota of the cell (NCu) (Droop, 1974, Thornton, 2014, Flynn and Flynn, 1998). Furthermore, the maximum rate of photosynthesis depends on the organism’s physiology, i.e. their capability to overcompensate the photosynthetic rate (relPS) (Geider, 1993). The maximum photosynthetic rate along with the initial slope ($\alpha^{Chl}$) and the photon flux density (PFD) are used to calculate gross photosynthesis (Cfix) using the Smith equation (Smith, 1936). The net photosynthesis rate (netPS) is determined by subtracting the loss through leakage (Cleak).

Primarily phototrophic organisms such as diatoms, green algae and CMs can regulate their chlorophyll-a synthesis (synChl - see Fig. 4(a)) (Geider and Piatt, 1986). If the cell is nutrient limited or the cell fixed too much carbon, the synthesis of chlorophyll-a is repressed (Moreno and Martiny, 2018). Under low light, the synthesis of chlorophyll-a is enhanced (Sukenik et al., 1987). Chlorophyll-a is also decomposed with a linear relationship to the nitrogen status (degChl) (Wirtz and Pahlow, 2010). Primarily phagotrophic organisms such as NCMs cannot produce their own chloroplasts, so they acquire them from prey (upChl). This acquisition is limited by a maximum chlorophyll-a:carbon quota via a sigmoidal function (see Fig. 4(b)). Those acquired chloroplasts are subsequently lost at a fixed linear rate (lossChl) (Ghyoot et al., 2017).

#### 2.3.4 Phagotrophy

The phagotrophic functions can be divided into four subsections: determining the prey capture, determining the prey quality, determining the predator ingestion rate and determining the predator assimilation rate. Table A.15 summarizes the description of the auxiliaries and Appendix A.6.5 provides the detailed mathematical equations.

The prey capture depends on the motility of predator and prey as well as the density of the prey. The motility (mot) is derived from a linear regression by Flynn and Mitra (2016) that uses the organisms’ equivalent spherical diameter as an input. The density of the prey (nrPrey) is calculated from the cellular carbon content (Ccell) and the current carbon protist state variable (protC). The motility of predator and prey as well as the density of prey are input parameters to determine the encounter rate (enc) according to the empirical Rothschild equation (Rothschild and Osborn, 1988). This encounter rate multiplied with the optimum capture rate (optCR) of the predator and the predator specific prey handling index (PR) determines the amount of specific prey the predator can capture. This is summed over all prey items (sumCP). As mixoplankton do not have the same capacity to ingest prey in the dark as in light (Skovgaard, 1996, Anderson et al., 2018), a light-dependent inhibition curve (inhLight - sigmoidal curve) is multiplied with the encounter rate and limits the capture of prey depending on the light availability. The light-dependent inhibition curve takes the photon flux density as well as the parameter relPhag (fraction of prey that can be ingested in the dark) as input.

The prey quality determines the assimilation efficiency (opAE) of the predator. A decrease in prey quality leads to a decrease in assimilation efficiency (Elser et al., 2000). The nutrient quota of the captured prey is compared against the nutrient quota of the predator. This returns a value between minimum (AEo) and the maximum assimilation efficiency (AEm see solid, black line in Fig. 5).

The predator ingestion rate (ingC - see Fig. 5) at very low prey densities is limited by the amount of prey (sumCP) that can be captured and at very high prey densities by the predator’s satiation (ingSat) (Flynn and Mitra, 2016). This satiation ingestion rate is calculated using a Holling type II curve (Holling, 1959) scaled to its maximum ingestion rate (maxIng). The maximum ingestion covers the maximum growth rate and basal respiration rate taking the quality of the captured prey into account. The ingestion of the other prey nutrients (ingN, ingP) is referenced to the carbon ingestion and the prey nutrient quota.

The predator assimilation rate (assC) is determined by taking the carbon specific ingestion rate and limiting it to the assimilation efficiency. The assimilation of the other prey nutrients (assN, assP) is referenced to the carbon assimilation and the optimum predator nutrient quota. Non-assimilated prey is voided as particulate organics, i.e. POCout, PONout and POPout.

### 2.4 AEM application

The module PROTIST was implemented in the software Delft3D-WAQ. Delft3D-WAQ solves the advection–diffusion reaction equation on a predefined grid and is part of the open-source modelling suite Delft-3D maintained by Deltares (Deltares, 2020).

The AEM of this study employs established Delft3D-WAQ modules to simulate nutrients (NH${}_{4}^{+}$, NO${}_{3}^{-}$, PO${}_{4}^{3-}$ and Si), organic matter i.e particulate organic nitrogen (PON), phosphate (POP) and carbon (POC) as well as opal, dissolved oxygen, solar radiation and suspended sediment. The modules compute the settling of organic matter, the decomposition of organic matter, the dissolution of silica, nitrification and denitrification, the extinction of light as well as the reaeration of the water column. For more information on those modules, the authors refer to Blauw et al. (2009).

To simulate primary production, this AEM employs PROTIST. Although the aim was to run the AEM with all five PFTs, it was difficult to parameterize NCMs for this AEM application using literature and data-based knowledge. Unfortunately, NCMs are not sampled in the routine monitoring program of the SNS (Schneider et al., 2020), so there is a lack of knowledge about the distribution of NCMs in the SNS. Furthermore, there is still a lack of physiological understanding of NCMs (Hansen et al., 2019). So, for this AEM, we were only able to simulate four PFTs: diatom, green, protozooplankton and CM.

However, using a steady-state box model, we successfully demonstrated growth and competition between the five PFTs in a simplified, idealized environment. For more details on this technical test, the authors refer to Appendix D.

#### 2.4.1 Model domain

The SNS was chosen as a model domain as it is a well-monitored shelf sea that covers strong abiotic gradients. Abiotic and biotic parameters are routinely monitored at 11 location classes by the Rijkswaterstaat monitoring program (Dutch Directorate-General for Public Works and Water Management). Schneider et al. (2020) showed that these 11 location classes can be grouped into four environmental systems based on dissolved inorganic nutrient concentrations, suspended sediment concentrations and water column stratification. These four environmental systems are (a) the unstratified estuary systems (ES) with high dissolved inorganic nutrient and high suspended sediment concentrations, (b) the unstratified coastal systems (CS) with lower dissolved inorganic nutrient and suspended sediment concentrations compared to the estuary systems, (c) the anthropogenically modified systems (AS) that are characterized by high dissolved inorganic nutrient but low suspended sediment concentrations and (d) the offshore systems (OS) that have low dissolved inorganic nutrient and low suspended sediment concentrations throughout the year. Fig. 6 shows the geographic location of these routine monitoring location classes.

#### 2.4.2 Model schematization

11 1D-V models, consisting of two model cells each, were constructed to mimic these 11 location classes of the SNS. The 11 1D-V models differ from each other in dissolved inorganic nutrient and suspended sediment concentration boundary conditions as well as depth and stratification. Salinity was not considered. The dissolved inorganic nutrient and suspended sediment concentrations forced at the boundaries were derived from monthly averaged data (see fig. B.1 - B.5 in Appendix B). Total nitrogen was distributed in a ratio of 5:1 to the ${\mathrm{NO}}_{3}^{-}$ and ${\mathrm{NH}}_{4}^{+}$ timeseries. Total phosphate and silica were used as an input for PO${}_{4}^{3-}$ and SiO_2_, respectively. The depth was determined from the average depth at the location classes. Stratification was applied to the location classes Veerse Meer, Grevelingen, Offshore and Doggerbank during the summer months by decreasing the diffusion parameter in the model set-up. Fig. 7 visualizes the physical attributes of the 11 1D-V models.

The same temperature and radiation timeseries were applied to all 1D-V models (see fig. C.1 and C.2 in Appendix C), as the geographical extent of the SNS is small enough to allow this simplification. Furthermore, the transport through the 1D-V models was determined in such a way that the water residence time for all 1D-V models was equal (30 days). A very low biomass concentration of each PFT was applied to the boundaries at all 11 1D-V models to ensure that there is always seeding biomass available for each PFT. Lastly, the 1D-V models were run with a timestep of 3 min from 2000 to 2010 and an output timestep of 2h. The first year was used as a spin-up.

#### 2.4.3 Model parameterization

For the PFTs capable of nutrient uptake, the minimum, optimum and maximum nutrient quotas (e.g. NCmin, NCopt, NCmax) for the different PFTs were derived from Leonardos and Geider (2004). The minimum and optimum nitrogen:carbon quotas (PCminNCmin and PCminNCmax) during phosphate limitation were calibrated using the quotas from Leonardos and Geider (2004). The optimum and maximum nitrogen:carbon quotas for the uptake of nitrate (NO3Copt and NO3Cmax) were set to be slightly lower than the optimum and maximum nitrogen:carbon quotas for the uptake of ammonium (NCopt and NCmax). The minimum, optimum and maximum nutrient quotas (e.g. NCmin, NCopt, NCmax) for protozooplankton were set according to Flynn (2021).

The maximum chlorophyll-a:carbon quota (ChlCmax) as well as the initial slope $\alpha^{Chl}$ for the different PFTs were set according to averages per class taken from Geider et al. (1997). Phototrophic organisms have an overcapacity for photosynthesis in order to cover their loss rates (Geider et al., 1998), so the dimensionless parameter relPS (the ratio of photosynthesis rate to maximum growth rate) was set to 2 for the primarily phototrophic organisms.

Previous studies (Skovgaard, 1996, Li et al., 1999, Adolf et al., 2006, Anderson et al., 2018) showed that CMs ingest very little prey in the dark, so ingestion of prey by CMs is light dependent via the dimensionless parameter relPhag. Jeong et al. (2010) showed that CMs cannot capture prey larger than themselves. This was implemented by setting the prey handling index (PR) for each predator accordingly. The sedimentation of diatoms (sed) was set according to Stokes law. A variable density in diatoms due to vacuole was not implemented. Lastly, the size of each PFT was derived from the median size per PFT from the protist dataset used in Schneider et al. (2020) that covers the same study area.

All 1D-V models were initialized with the same biomass values and the same reference maximum growth rate for each PFT (UmRT = 0.81 d^−1^). Thus, no initial advantage was given to any PFT. A linear mortality function with a reference mortality of 0.07 d^−1^ (Blauw et al., 2009) was applied to the modelled diatom, green algae and CM. A quadratic mortality function with a reference mortality of 0.007 d^−1^ was applied as a closure function to the modelled protozooplankton. Table A.3 summarizes PFT specific parameters established through literature.

#### 2.4.4 Model validation

Routine monitoring data on the SNS provided the in-situ comparison data for the 1D-V model runs (Schneider et al., 2020). A quantitative comparison of the SVs was done in a target diagram. A target diagram compactly visualizes standard deviations, bias and correlations between model results and field observations (Joliff et al., 2009). On target diagrams, the unity circle provides a marker for the quantification of the fit between model results and observations. SVs that lie within the unity circle are positively correlated and perform well compared to observations. SVs that lie outside of the unity circle have a significant bias and difference in variance between model results and observations. Furthermore, modelled phytoplankton biomass as well as chlorophyll-a timeseries were compared visually against field data timeseries. Nutrients were not compared as they are forced with the transport (see figures B.1 - B.5 in Appendix B). The modelled trophic composition was compared qualitatively to the in-situ trophic composition provided in Schneider et al. (2020). The trophic compositions were calculated as fractions per PFT of the total protist biomass.

#### 2.4.5 Sensitivity analysis

A sensitivity analysis was completed to determine the influence of different abiotic factors on the biomass concentration of CMs. To verify that the abiotic factors had variations in the same order of magnitude, the normalized standard deviation was calculated ($\bar{sd_{x}}=sd_{x}/mean_{x}$). In separate model runs, the inorganic nutrient concentrations and the suspended sediment concentration were modified by 10% and the resulting CM biomass analysed.

## 3 Results

### 3.1 PFT timeseries

Fig. 8 displays results of the eleven 1D-V models for the years 2001 to 2010. It shows the carbon biomass of each PFT within each 1D-V model over the whole timeframe. The 1D-V model results can be grouped into four categories that align with the environmental systems previously described (see Fig. 6, Fig. 7).

This figure highlights five important aspects. Firstly, the 1D-V models were unable to capture the dynamics of estuary systems (ES) with protist biomass near zero along the 10-year simulation in both the Westerschelde and Waddensea location classes. Through tidal mixing these systems import protist biomass produced in neighbouring coastal waters where conditions are more favourable. Since this transport of PFTs is not included in these simple 1D-V models, it can be expected that the model underestimates PFT values within the 1D-V estuary models and, thus, the model results of the ES must be neglected. Secondly, the biomass order of magnitude varies between the 1D-V models with the coastal systems (CS) (Oosterschelde to Coastal Waddensea) displaying the highest peaks in biomass and the offshore systems (OS) the lowest (Offshore Mixed to Doggerbank). Thirdly, in each 1D-V model, the effect of the year-to-year variations of the nutrient and suspended sediment boundary conditions (see figures B.1 - B.5 in Appendix B) are visible in the spring bloom strength, timing and composition. Fourthly, the onset of the spring bloom is the earliest in the 1D-V models that are stratified (Veerse Meer, Grevelingen, Offshore and Doggerbank). Lastly, the 1D-V models of the OS display protists throughout the whole year, while the 1D-V models of the CS display stark peaks at the beginning of spring.

**Fig. 9:**
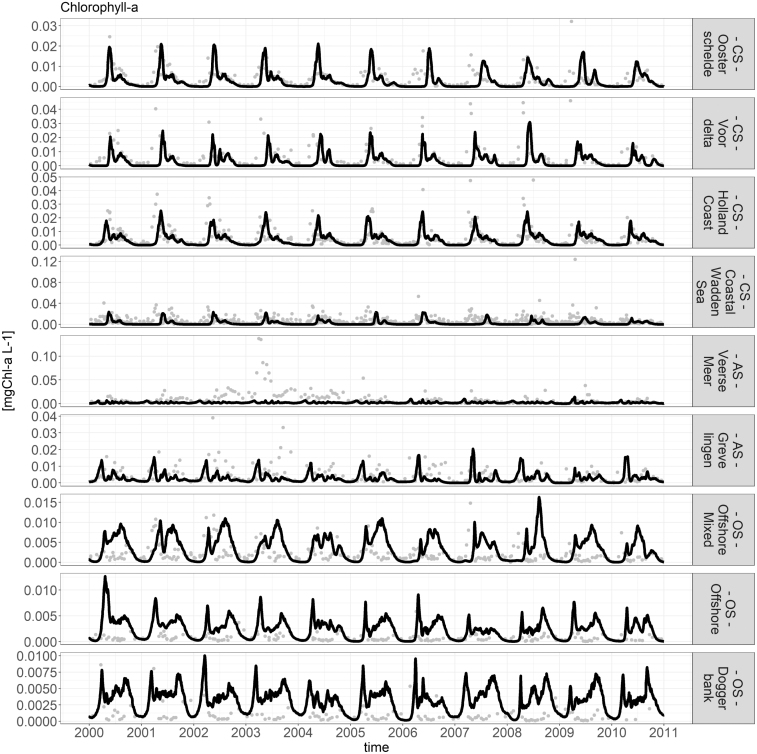
Comparison of model chlorophyll-a (line) and data chlorophyll-a (points).

**Fig. 10:**
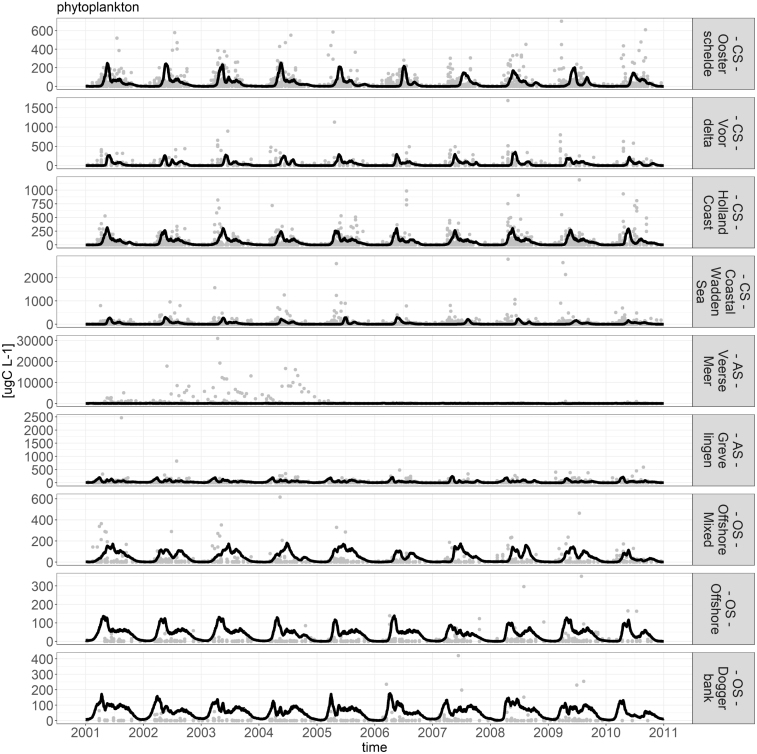
Comparison of model phytoplankton (line) and data phytoplankton (points).

**Fig. 11:**
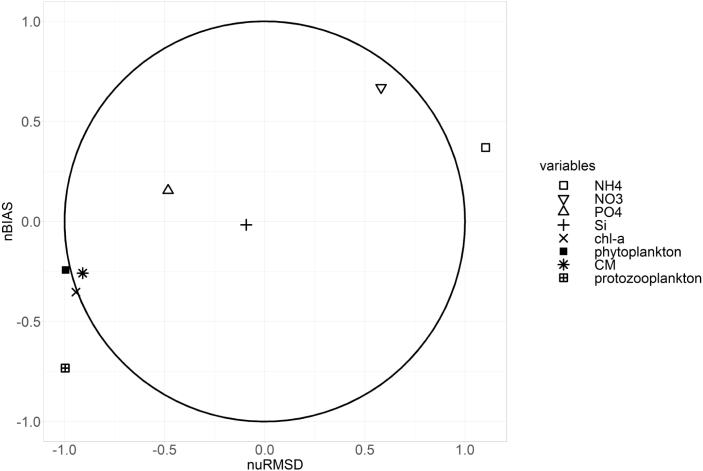
Target diagram visualizing the model-sample data comparison (nuRMSD — normalized root-mean square difference; nuBIAS — normalized bias). Silica, nitrate, phosphate, chlorophyll-a, CM and phytoplankton lie within the unity circle which shows that the model performs well compared to the in-situ data. Ammonium and protozooplankton display a significant bias and difference in variance between the model results and the in-situ data.

**Fig. 12:**
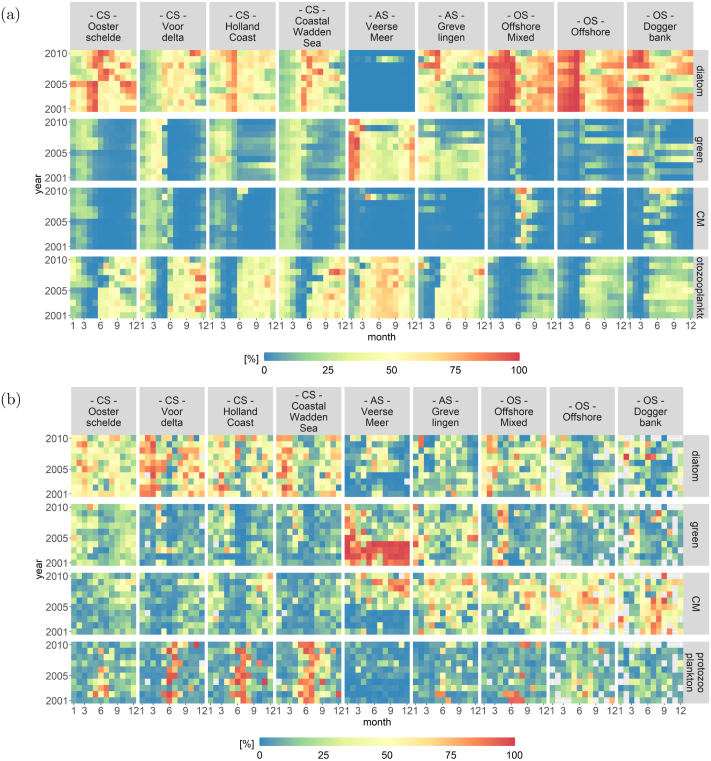
Heatmaps displaying the monthly fractions of the total biomass per PFT from (a) the 1D-V models mimicking the SNS and (b) the routine monitoring data.

Modified from Schneider et al. (2020).

**Fig. 13:**
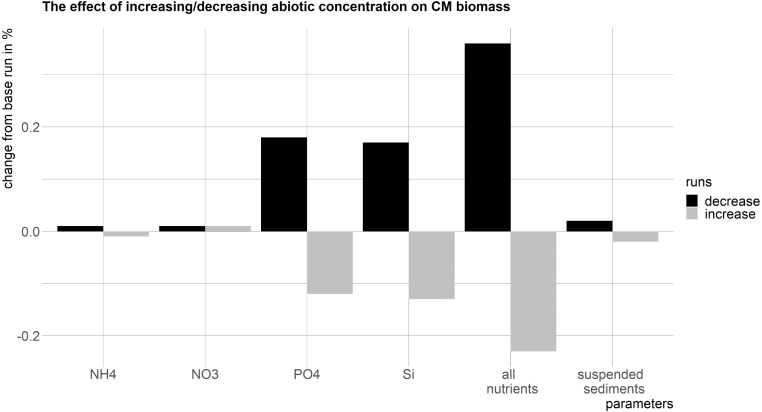
The sensitivity analysis shows that changing the nutrient concentration, specifically phosphate and silica concentrations, has the largest effect on the CM biomass. The values display the changes from the base run in %.

### 3.2 Quantitative validation

Fig. 9, Fig. 10 show the comparison between the model and data for chlorophyll-a and phytoplankton biomass. These two variables were chosen as they have the most reliable and complete data source. The figures show that the model manages to capture the most relevant dynamics. Especially in the CS, the model results for chlorophyll-a and phytoplankton biomass compare well against observations. In the OS, both the chlorophyll-a and phytoplankton biomass tend to be overestimated by the model in the late summer months. The figures also show that the model did not manage to capture the dynamics of Veerse Meer.

Fig. 11 displays model-data comparisons for inorganic nutrients, carbon biomass of the different PFTs and chlorophyll-a in a target diagram. It should be noted that the ES Westerschelde and Waddensea were not taken into account as the dynamics of those location classes could not be captured by the 1D-V models. Furthermore, to determine whether the 1D-V models manage to capture the difference in order of magnitude between the location classes, the maximum values for the protist and chlorophyll-a state variables per year and location class were extracted. Therefore, this target diagram evaluates the ability of the models to capture the variation over the whole biogeochemical gradient.

Three important aspects can be highlighted in Fig. 11. Firstly, all nutrients, except for ammonium, lie within the unity circle and thus compare well to the sampled data. This is not unexpected as the nutrient timeseries are transported into the column models via the boundary. Secondly, phytoplankton, CM and chlorophyll-a lie on the boundary of the unity circle and thus also compare well to the observations. Lastly, ammonium and protozooplankton lie outside the unity circle and thus show significant bias and difference in variance between the model results and the in-situ data.

### 3.3 Qualitative validation of the trophic composition

Fig. 12 displays the protist community composition of the 1D-V models (Fig. 12(a)) and the monitoring data (Fig. 12(b)) over the whole timeframe for environmental systems except the ES. The ES were removed because the 1D-V schematization was not able to capture the dynamics of those systems. The colours depict the percentage of each PFT. Fig. 12 can once again be divided according to the environmental systems and both figures display a increasing gradient of CMs from the CS to the OS.

It must be noted that there are severe shortcomings in the protozooplankton identification of the monitoring program, as it is geared towards identifying phytoplankton. Mainly easily recognizable protozooplankton such as *Noctiluca scintillans* were identified and, thus, the protozooplankton of the routine monitoring data in Fig. 12(b) are only indicative for protozooplankton occurrence in the 1D-V models.

The CS of the 1D-V model runs are mainly dominated by diatoms, but also green algae and CMs until June and are succeeded by the occurrence of protozooplankton (Fig. 12(a)). In the data analysis (Fig. 12(b)), the CS are also dominated by a spring phytoplankton bloom, followed by a very distinct bloom of protozooplankton.

The 1D-V model run of the anthropogenically modified system (AS) Veerse Meer (Fig. 12(a)) is characterized by a lack of diatoms and CMs compared to the measured concentrations (Fig. 12(b)). The biomass is almost evenly divided among green algae and protozooplankton. During the first part of the simulation period (until 2007), the biomass of the 1D-V model run of the AS Grevelingen is divided fairly even between green algae, protozooplankton and diatoms. After 2007, the fraction of diatoms increases in the simulated results (Fig. 12(a)). In the data analysis (Fig. 12(b)), the AS are characterized by a lack of protozooplankton. Apart from an obvious dominance of green algae in Veerse Meer before 2004, the measured biomass is fairly evenly divided among diatoms, green algae and CMs.

The OS of the 1D-V model runs are characterized by a dominant diatom bloom in spring, succeeded by a bloom of CMs. Protozooplankton also occur to a lesser extent in the offshore 1D-V models compared with the other systems. Compared to the measured concentrations, the Offshore Mixed and Doggerbank model runs perform well as they display a dominance of diatoms at the beginning of the year followed by CMs. However, the modelled CM occurrence ends earlier in the year compared to the measured concentrations.

### 3.4 Sensitivity analysis to test CM occurrence hypothesis

The normalized standard deviations of the abiotic factors verified that the variations for the abiotic factors are in the same order of magnitude (see table E.1 in Appendix E). Thus, the sensitivity analyses of the different abiotic factors are comparable.

The sensitivity analysis results show that the CM biomass changes anti-proportionally to the dissolved inorganic nutrient concentrations (see Fig. 13). A decrease of dissolved inorganic nutrient concentrations leads to an increase of CM biomass and vice versa. The sensitivity analyses in which each dissolved inorganic nutrient was modified independently show that a decrease of phosphate and of silica result in an increase of CM biomass, while an increase of phosphate and silica result in a decrease of CM biomass. However, increasing or decreasing the suspended sediment, ammonium or nitrate concentration by 10% does not result in similar changes of the CM biomass. Thus, changes in phosphate and silica concentrations have a larger relative effect on the CM biomass.

## 4 Discussion

In this study, we introduced PROTIST, a module that calculates the primary production of and competition within protist communities. The aim of this study was to estimate the ability of the module PROTIST to simulate the growth and mortality of a protist community and to further explore the hypothesis put forward by Schneider et al. (2020) that the biogeochemical gradient drives the trophic composition of protist communities in the SNS. By applying the module PROTIST to a group of 1D-V models that mimic the biogeochemical gradient of the SNS, we were able to show that it responds to different biogeochemical forcings and results in different, plausible trophic compositions that are in line with observed data (see Fig. 12).

CMs have often been shown to occur in oligotrophic environments (Stoecker and Lavrentyev, 2018, Duhamel et al., 2019). Using state-of-the art knowledge on protist physiology, trophic pathways and protist parameters, this modelling study shows that CMs are likely to occur in environments and during months with low dissolved inorganic nutrient supply. The sensitivity analysis showed that the availability of dissolved inorganic phosphate and silica strongly influenced the occurrence of mixoplankton. However, the availability of dissolved inorganic nitrogen had little effect on the occurrence of mixoplankton, which is most likely due to the fact that dissolved inorganic nitrogen concentrations are rarely limiting in the North Sea coastal zone (Philippart et al., 2007). The suspended sediment gradient (which affects light availability) had very little impact on the occurrence of mixoplankton. Using experimental data, Li et al. (2000) and Smalley et al. (2012) found that nutrient limitation induces phagotrophy in mixoplankton. So, based on the model results, we can conclude that in the SNS the biogeochemical gradient drives the trophic composition of the protist community primarily through the availability of dissolved inorganic nutrients such as phosphate and silica.

The chosen schematization and set-up of the 1D-V models proved useful as a first approach, but also shows some caveats. While the 1D-V models of the CS and OS perform well compared to the observations, the 1D-V models of the ES and AS perform poorly. The poor performance in the ES is due to three reasons. Firstly, the lack of transport of biotic SVs from coastal waters into the estuary environments and secondly, the lack of tidal dynamics. Most of the organic carbon stems from allochthonous sources (Soetaert and Herman, 1995) and tides dominate the estuaries (Heip, 1988). Thirdly (in concert with the tidal dynamics), the depth distribution of the location classes is not captured in the 1D-V models. There are very shallow places where growth can occur, but as we used the average depth over all sampling locations per location class this is not represented in the current schematization. A 3D grid with hydrodynamics that include transport and stratification could improve simulations also with regard to the timing and duration of blooms.

The 1D-V models of the AS perform poorly as the anthropogenic impact on the hydrodynamics of those systems was not considered. This is clearly visible in the lack of modelled CMs (see Fig. 12). In general, it is difficult to capture the dynamics of those AS with the limited hydrodynamics of the generic 1D-V models. In 2004, Veerse Meer was re-opened to the Oosterschelde thus allowing exchange of water masses between the Oosterschelde and Veerse Meer (Wijnhoven et al., 2010) turning the freshwater lake into a marine fjord-like water body. During the transition period (2002–2004), a period of high phytoplankton biomass was observed likely due to the absence of benthic grazers (RIKZ, 2007). This could not be captured by the 1D-V models. Grevelingen was also hydrodynamically altered during the 10-year time period (Hoeksema, 2002).

Furthermore, the occurrence of green algae was low in all 1D-V models with the exception of the AS Veerse Meer and Grevelingen. The main reason for this lies in the lack of modelling a defining trait of *Phaeocystis*, a phytoplankton which commonly occurs in the Dutch SNS. *Phaeocystis* avoids predation by forming colonies (Lancelot et al., 2005), a trait currently not included in PROTIST.

Lastly, the organisms’ size determines competition between the organisms (Finkel, 2007). To have a point of reference, the size of the PFTs was derived from routine monitoring data by taking the median size per PFT. However, protist sizes within a community are often not normally distributed, but rather bi- or multimodal. As there is only one size per PFT and the handling rate of the prey depends mainly on the relation between predator and prey size, the chosen size of the organisms impacts the model results. Applying multiple species per PFT may give better results, but will also increase calculation times. At the same time, with only one species per PFT, the model results look realistic. We conclude that while these 1D-V models have caveats, the model results are realistic and the caveats correspond to the chosen model schematization.

An interesting outcome of this study was the coupling of the trophic pathways for CMs within PROTIST. Even though the model equations used in PROTIST for the different trophic pathways are quite detailed in their physiological descriptions, the trade-off between the phototrophic and phagotrophic pathways for CMs is not. Both the phototrophic and phagotrophic pathways are accessible to CMs. The efficiency of each trophic pathway per PFT is set using measured physiological parameters derived from literature. CMs have a lower affinity to light (lower $\alpha^{Chl}$) and a lower chlorophyll-a:carbon quota compared to diatoms and green algae (Geider et al., 1997). Additionally, CMs have very low ingestion rates during night (Skovgaard, 1996, Li et al., 1999, Adolf et al., 2006, Anderson et al., 2018) compared to protozooplankton. So, it can be hypothesized that CMs employ their mixotrophic genes to remain competitive (Litchman, 2007).

Consequently, CMs do well in environments and seasons in which there is an advantage of combining both trophic modes (Hartmann et al., 2012). Such environments or seasons have a low supply of dissolved inorganic nutrients and/or prey (Mitra et al., 2014). The great ocean gyres can be classified as such environments and so it is not surprising that recent research has found mixoplankton to occur notably in the world’s oceans (Faure et al., 2019). Global warming and the construction of offshore windfarms may change the pelagic environment towards stronger stratification and longer nutrient limitation (Falkowski, 1994, Richardson and Schoeman, 2003, Falkowski and Oliver, 2007). This could allow CMs to become successful due to their mixotrophic genes. As many harmful algal blooms are caused by CMs (Burkholder et al., 2008), it is important for managers to have access to adequate monitoring and modelling techniques with which to assess the probability of potentially harmful CMs occurring (Peperzak, 2003).

## 5 Conclusion

This study has shown that the newly developed module PROTIST for Delft3D-WAQ is capable of modelling different PFTs that interact with, compete against and graze on each other. The module PROTIST responds to biogeochemical gradients and results in different trophic compositions for the protist communities very similar to in-situ observations in those simulations where comparison is useful. Furthermore, this study used modelling results to provide a layer of evidence that the availability of dissolved inorganic phosphate and silica drives the occurrence of CMs in a system with strong gradients of dissolved inorganic nutrients and suspended sediments.

This study demonstrates that PROTIST can be applied in an AEM setting. AEMs provide an important tool to help understand and predict the consequence of changing pressures on the productivity of an ecosystem (Schuwirth et al., 2019). Especially against the background of future anthropogenic changes in coastal environments, it is important that AEMs, such as the one presented here, can model the main trophic pathways within the protist community under dynamic conditions.

## CRediT authorship contribution statement

**Lisa K. Schneider:** Undertook the primary model development, Simulations, Preparation of the figures, Writing - original draft, Contributed to the development and interpretations of the subsequent manuscript revisions. **Nathalie Gypens:** Contributed to the development and interpretations of the subsequent manuscript revisions. **Tineke A. Troost:** Contributed to the development and interpretations of the subsequent manuscript revisions. **Willem Stolte:** Contributed to the development and interpretations of the subsequent manuscript revisions.

## Declaration of Competing Interest

The authors declare that they have no known competing financial interests or personal relationships that could have appeared to influence the work reported in this paper.

## Appendix A Supplementary data

MMC S1 Model equations and parameters for the primary production module PROTIST and additional information for PROTIST application.
