# Corrigendum to ‘Modeling mixoplankton along the biogeochemical gradient of the Southern North Sea’ Ecological Modelling 458 (2021) 0304-3800/109690

**DOI:** 10.1016/j.ecolmodel.2023.110374

**Published:** 2023-07

**Authors:** Lisa K. Schneider, Natalie Gypens, Tineke A. Troost, Willem Stolte

**Affiliations:** a Boussinesqweg 1, 2629 HV Delft, South Holland, The Netherlands; b Université Libre de Bruxelles, Laboratoire d'Ecologie des Systèmes Aquatiques, CP221, Boulevard du Triomphe, B-1050, Belgium

DOI of original article: https://doi.org/10.1016/j.ecolmodel.2021.109690

The authors regret to submit following correction of the published article listed above. The corrected supplementary material can be found on the following pages. The authors would like to apologise for any inconvenience caused.

## Appendix Supplementary materials

Image, application 1

