# Supplementary material for: Modeling mixoplankton along the biogeochemical gradient of the Southern North Sea
Source: Ecol Modell. 2021 Nov 1;459:109690. doi: 10.1016/j.ecolmodel.2021.109690 (PMC8507435; doi:10.1016/j.ecolmodel.2021.109690)
Supplement: MMC S1 — Model equations and parameters for the primary production module PROTIST and additional information for PROTIST application. [file mmc1.pdf]

Appendix A. Model description

Appendix A.1. Model SV

Table A.1: List of all model state variables, their description, unit and initial value. Values labeled with a * differ for each location class.

state variable	state variable description	unit	value
PO4	initial DIP	gP m^{-3}	*
NH4	initial NH_4^+	gN m^{-3}	*
NO3	initial NO_3^-	gN m^{-3}	*
Si	initial Si	gSi m^{-3}	*
Opal	Opal-Si	gSi m^{-3}	*
POC1	POC1 (fast decomposing fraction)	gC m^{-3}	0.0
PON1	PON1 (fast decomposing fraction)	gN m^{-3}	0.0
POP1	POP1 (fast decomposing fraction)	gP m^{-3}	0.0
POS1	POS1 (fast decomposing fraction)	gS m^{-3}	0.0
DOClab	labile DOC	gC m^{-3}	0.0
OXY	oxygen	gO2 m^{-3}	0.0
greenC	green C-biomass	gC m^{-3}	0.01
greenChl	green Chl-biomass	gChl m^{-3}	0.0002
greenN	green N-biomass	gN m^{-3}	0.0015
greenP	green P-biomass	gP m^{-3}	0.00024
diatC	diatom C-biomass	gC m^{-3}	0.01
diatChl	diatom Chl-biomass	gChl m^{-3}	0.0002
diatN	diatom N-biomass	gN m^{-3}	0.0015
diatP	diatom P-biomass	gP m^{-3}	0.00024
diatSi	diatom Si-biomass	gSi m^{-3}	0.002
cmC	CM C-biomass	gC m^{-3}	0.01
cmChl	CM Chl-biomass	gChl m^{-3}	0.0002
cmN	CM N-biomass	gN m^{-3}	0.0015
cmP	CM P-biomass	gP m^{-3}	0.00024
zooC	protozooplankton C-biomass	gC m^{-3}	0.01
zooN	protozooplankton N-biomass	gN m^{-3}	0.0015

Continued on next page

Table A.1 – *Continued from previous page*

**state variable**	**state variable description**	**unit**	**value**
zooP	protozooplankton P-biomass	gP m^{-3}	0.00024
ncmC	NCM C-biomass	gC m^{-3}	0.01
ncmChl	NCM Chl-biomass	gChl m^{-3}	0.0002
ncmN	NCM N-biomass	gN m^{-3}	0.0015
ncmP	NCM P-biomass	gP m^{-3}	0.00024

Appendix A.2. Model parameters

Table A.2: List of all model parameters for a generic PFT, their description, unit and default value. Values labeled with a * can be found in table A.3.

**parameter**	**parameter description**	**unit**	**value**
AEm	maximum assimilation efficiency (AE)	dl	0.6
AEo	minimum AE	dl	0.3
alpha	alpha for photosynthesis in protist	gC $gChl^{-1}$ m^2 $umol^{-1}$ photon	*
abcChl	light absorbance coefficient for chlorophyll	m^2 $gChl^{-1}$	20
Ccell	C content of protist cell	pgC cell-1	*
ChlCm	maximum cellular Chl:C ratio	gChl gC^{-1}	*
ChlCo	minimum cellular Chl:C ratio	gChl gC^{-1}	0.001
CR	catabolic respiration quotient	dl	0.05
degChl	Chl degradation	d^{-1}	0.72
FrAut	fraction of mortality to autolysis	dl	0.3
FrDet	fraction of mortality to detritus	dl	0.7
kAE	control of AE in response to prey quality	dl	1.00E+03
KtNH4	Kt for NH_4^+ transport	gN m^{-3}	0.007
KtNO3	Kt for NO_3^- transport	gN m^{-3}	0.007
KtP	Kt for DIP transport	gP m^{-3}	0.031
MrtRT	mortality at reference temperature	dl	*
Mphoto	acclimation rate to light	dl	0.5
NCm	N:C that totally represses NH_4^+ transport	gN gC^{-1}	*
NCo	minimum N-quota	gN gC^{-1}	*
NCopt	N:C for growth under optimal conditions	gN gC^{-1}	*
NO3Cm	N:C that totally represses NO_3^- transport	gN gC^{-1}	*
NO3Copt	N:C for growth on NO_3^- under optimal conditions	gN gC^{-1}	*
optCR	proportion of prey captured by starved Zoo	dl	0.1
PCm	PC maximum quota	gP gC^{-1}	*
PCo	PC minimum quota	gP gC^{-1}	*
PCoNCm	maximum NC when PC is minimum (PCu = 0)	gN gC^{-1}	*
PCoNCop	optimum NC when PC is minimum (PCu = 0)	gN gC^{-1}	*

Continued on next page

Table A.2 – *Continued from previous page*

**parameter**	**parameter description**	**unit**	**value**
PCopt	PC optimum quota	gP gC^{-1}	*
PSDOC	proportion of current PS being leaked as DOC	dl	0.1
Q10	Q10 for UmRT	dl	*
r	radius of nutrient repleted protist cell	um	*
redco	C respired to support nitrate reduction for NH_4^+	gC gN-1	1.71
relPhag	relative phagotrophy in night:day	dl	*
relPS	relative PSmax:Umax on phototrophy	dl	*
ReUmNH4	max. growth rate supported by NH_4^+:Umax	dl	0.9
ReUmNO3	max. growth rate supported by NO_3^-:Umax	dl	0.8
RT	reference temperature for UmRT	deg C	*
SDA	specific dynamic action	dl	0.3
UmRT	maximum growth rate at reference T	d^{-1}	*

Table A.3: Summary of the PFT specific parameters established through literature as stated in text. Note that the protozooplankton mortality (marked with *) uses a quadratic closure function, while the phytoplankton and CM mortality use a linear mortality function.

parameter	units	diatom	green algae	CM	protozoo-plankton	origin
ESD	μm	24.0	10.0	18.0	40.0	Schneider et al. (2020)
ChlCmax	gChl gC^{-1}	0.058	0.033	0.021	-	Geider et al. (1997)
α^{Chl}	gC $gChl^{-1}$ m^2 $umol^{-1}$ photon	9.5e-6	7e-6	7e-6	-	Geider et al. (1997)
NCmin	gN gC^{-1}	0.11	0.14	0.09	0.05	Leonardos & Geider (2004)
NCopt	gN gC^{-1}	0.15	0.17	0.12	0.15	Leonardos & Geider (2004)

Continued on next page

Table A.3 – *Continued from previous page*

parameter	units	diatom	green algae	CM	protozoo-plankton	origin
NCmax	gN gC^{-1}	0.2	0.2	0.2	0.2	Leonardos & Geider (2004)
PCminNCopt	gN gC^{-1}	0.12	0.15	0.1	-	calibrated using Flynn (2020)
PCminNCmax	gN gC^{-1}	0.13	0.16	0.11	-	calibrated using Flynn (2020)
NO3Copt	gN gC^{-1}	0.14	0.16	0.11	-	based on Leonardos & Geider (2004)
NO3Cmax	gN gC^{-1}	0.16	0.18	0.13	-	based on Leonardos & Geider (2004)
PCmin	gP gC^{-1}	0.009	0.02	0.006	0.005	Leonardos & Geider (2004)
PCopt	gP gC^{-1}	0.014	0.028	0.012	0.024	Leonardos & Geider (2004)
PCmax	gP gC^{-1}	0.029	0.036	0.028	0.05	Leonardos & Geider (2004)
relPS	dl	2	2	2	-	Geider et al. (1998)
relPhag	dl	-	-	0.1	1	Skovgaard (1996); Li et al. (1999); Adolf et al. (2006); Anderson et al. (2018)
PR diatom	dl	-	-	-	1	information from Jeong et al. (2010)

Continued on next page

Table A.3 – *Continued from previous page*

parameter	units	diatom	green algae	CM	protozoo-plankton	origin
PR green algae	dl	-	-	1	1	information from Jeong et al. (2010)
PR CM	dl	-	-	-	1	information from Jeong et al. (2010)
sed	m d^{-1}	0.38	-	-	-	Stokes law
mrt	d^{-1}	0.07	0.07	0.07	0.007 *	Blauw et al. (2009)

Appendix A.3. Model auxiliaries

Table A.4: List of all model auxiliaries for a generic PFT, their description and unit.

**auxiliary**	**auxiliary description**	**unit**
NC	cellular nitrogen:carbon ratio	gN gC^{-1}
PC	cellular phosphate:carbon ratio	gP gC^{-1}
SC	cellular silica:carbon ratio	gSi gC^{-1}
ChlC	cellular chlorphyll:carbon ratio	gChl gC^{-1}
UmT	temperature dependent maximum growth rate	gC gC^{-1} d^{-1}
BR	temperature dependent basal respiration rate	gC gC^{-1} d^{-1}
NCu	cellular nitrogen status	dl
PCu	cellular phosphate status	dl
SCu	cellular silica status	dl
NPCu	Liebig nutrient limitation	dl
mot	motility of the protist	m s-1
upP	uptake rate of phosphate	gP gC^{-1} d^{-1}
upNH4	uptake rate of ammonium	gN gC^{-1} d^{-1}
upNO3	uptake rate of nitrate	gN gC^{-1} d^{-1}
upSi	uptake rate of silica	gSi gC^{-1} d^{-1}
upChl	uptake rate of chlorphyll	gChl gC^{-1} d^{-1}
PSqm	maximum photosynthetic rate	gC gC^{-1} d^{-1}
PS	gross photosynthetic rate	gC gC^{-1} d^{-1}
Cfix	net photosynthetic rate	gC gC^{-1} d^{-1}
synChl	synthesis rate of chlorophyll-a	gChl gC^{-1} d^{-1}
degChl	degradation rate of chlorphyll	gChl gC^{-1} d^{-1}
sumCP	rate of all potential prey captures	gC gC^{-1} d^{-1}
ingNC	rate of captured nitrogen:carbon	gN gC^{-1} d^{-1}
ingPC	rate of captured phosphate:carbon	gP gC^{-1} d^{-1}
ppNC	ratio of captured prey nitrogen: predator nitrogen	dl
ppPC	ratio of captured prey nitrogen: predator nitrogen	dl
stoichP	limiting nutrient in prey	dl
opAE	assimilation efficiency of predator	dl
maxIng	maximum ingestion rate	gC gC^{-1} d^{-1}

Continued on next page

Table A.4 – *Continued from previous page*

**auxiliary**	**auxiliary description**	**unit**
ingSat	satiation ingestion rate	gC gC^{-1} d^{-1}
ingC	ingestion rate of prey carbon	gC gC^{-1} d^{-1}
assC	assimilation rate of prey carbon	gC gC^{-1} d^{-1}
ingN	ingestion rate of prey nitrogen	gN gC^{-1} d^{-1}
ingP	ingestion rate of prey phosphate	gP gC^{-1} d^{-1}
assN	assimilation rate of prey nitrogen	gN gC^{-1} d^{-1}
assP	assimilation rate of prey phosphate	gP gC^{-1} d^{-1}
totR	total respiration rate	gC gC^{-1} d^{-1}
Cu	carbon-specific growth rate	gC gC^{-1} d^{-1}
mrt	mortality rate	gC gC^{-1} d^{-1}
mrtAut	fraction of mortality rate towards autolysis	gC gC^{-1} d^{-1}
mrtDet	fraction of mortality rate towards detritus	gC gC^{-1} d^{-1}
lInh	light inhibition factor	dl

Appendix A.4. Model fluxes

Table A.5: List of all model fluxes for a generic PFT, their description and unit.

**flux**	**flux description**	**unit**
NH4up	uptake of NH_4^+ into algal biomass	gN m^{-3} d^{-1}
NO3up	uptake of NO_3^- into algal biomass	gN m^{-3} d^{-1}
Pup	uptake of PO_4^{3-} into algal biomass	gP m^{-3} d^{-1}
Siup	uptake of Si into algal biomass	gSi m^{-3} d^{-1}
Cfix	contribution to biomass growth from C-fixation	gC m^{-3} d^{-1}
Chlsyn	synthesis Chl rate of change	gChl m^{-3} d^{-1}
Chldeg	degradation Chl rate of change	gChl m^{-3} d^{-1}
Chlup	acquistion of prey Chl by NCM	gChl m^{-3} d^{-1}
Cresp	total respiration rate	gC m^{-3} d^{-1}
Cleak	release of DOC	gC m^{-3} d^{-1}
Cvoid	voiding of C as DOC if NC falls below NCo	gC m^{-3} d^{-1}
NH4out	NH_4^+ release by regeneration	gP m-1 d^{-1}
Pout	PO_4^{3-} release by regeneration	gN m-1 d^{-1}
Ceat	assimilation of C from prey	gC m^{-3} d^{-1}
Neat	assimilation of N from prey	gN m^{-3} d^{-1}
Peat	assimilation of P from prey	gP m^{-3} d^{-1}
POCout	rate of voiding of C as particulates	gC m^{-3} d^{-1}
PONout	rate of voiding of N as particulates	gN m^{-3} d^{-1}
POPout	rate of voiding of P as particulates	gP m^{-3} d^{-1}
AutC	protist-C mortality through Autolysis	gC m^{-3} d^{-1}
DetC	protist-C mortality through Detritus	gC m^{-3} d^{-1}
AutN	protist-N mortality through Autolysis	gN m^{-3} d^{-1}
DetN	protist-N mortality through Detritus	gN m^{-3} d^{-1}
AutP	protist-P mortality through Autolysis	gP m^{-3} d^{-1}
DetP	protist-P mortality through Detritus	gP m^{-3} d^{-1}
AutChl	protist-Chl mortality through Autolysis	gChl m^{-3} d^{-1}
DetChl	protist-Chl mortality through Detritus	gChl m^{-3} d^{-1}
D1C	mortality of prey i through predator j	gNut m^{-3} d^{-1}
D1Chl	mortality of prey i through predator j	gNut m^{-3} d^{-1}

Continued on next page

Table A.5 – *Continued from previous page*

**flux**	**flux description**	**unit**
D1N	mortality of prey i through predator j	gNut m^{-3} d^{-1}
D1P	mortality of prey i through predator j	gNut m^{-3} d^{-1}
D1Si	mortality of prey i through predator j	gNut m^{-3} d^{-1}

Appendix A.5. Conservation equations

Table A.6: Conservation equations for diatom SVs.

conservation equation		unit
$\frac{dDiat_C}{dt} = Diat_C \cdot (Cfix - Cleak - Cvoid - totR - mrt) - \sum Pred$	(A.1)	gC m^{-3} d^{-1}
$\frac{dDiat_N}{dt} = Diat_N \cdot (up_{NH4} + up_{NO3} - Nout - mrt) - \sum Pred$	(A.2)	gN m^{-3} d^{-1}
$\frac{dDiat_P}{dt} = Diat_P \cdot (up_{PO4} - Pout - mrt) - \sum Pred$	(A.3)	gP m^{-3} d^{-1}
$\frac{dDiat_{Si}}{dt} = Diat_{Si} \cdot (up_{Si} - mrt) - \sum Pred$	(A.4)	gSi m^{-3} d^{-1}
$\frac{dDiat_{Chl}}{dt} = Diat_{Chl} \cdot (synChl - degChl - mrt) - \sum Pred$	(A.5)	gChl m^{-3} d^{-1}

Table A.7: Conservation equations for green algae SVs.

conservation equation		unit
$\frac{dGreen_C}{dt} = Green_C \cdot (Cfix - Cleak - Cvoid - totR - mrt) - \sum Pred$	(A.6)	gC m^{-3} d^{-1}
$\frac{dGreen_N}{dt} = Green_N \cdot (up_{NH4} + up_{NO3} - Nout - mrt) - \sum Pred$	(A.7)	gN m^{-3} d^{-1}
$\frac{dGreen_P}{dt} = Green_P \cdot (up_{PO4} - Pout - mrt) - \sum Pred$	(A.8)	gP m^{-3} d^{-1}
$\frac{dGreen_{Chl}}{dt} = Green_{Chl} \cdot (synChl - degChl - mrt) - \sum Pred$	(A.9)	gChl m^{-3} d^{-1}

Table A.8: Conservation equations for protozooplankton SVs.

conservation equation		unit
$\frac{dZoo_C}{dt} = Zoo_C \cdot (assC - POCout - totR - mrt)$	(A.10)	gC m^{-3} d^{-1}
$\frac{dZoo_N}{dt} = Zoo_N \cdot (assN - PONout - mrt)$	(A.11)	gN m^{-3} d^{-1}
$\frac{dZoo_P}{dt} = Zoo_P \cdot (assP - POPout - mrt)$	(A.12)	gP m^{-3} d^{-1}

Table A.9: Conservation equations for CM SVs.

conservation equation		unit
$\frac{dCM_C}{dt} = CM_C \cdot (Cfix + assC - Cleak - Cvoid - POCout - totR - mrt) - \sum Pred$	(A.13)	gC m^{-3} d^{-1}
$\frac{dCM_N}{dt} = CM_N \cdot (up_{NH4} + up_{NO3} - assN - Nout - PONout - mrt) - \sum Pred$	(A.14)	gN m^{-3} d^{-1}
$\frac{dCM_P}{dt} = CM_P \cdot (up_{PO4} + assP - Pout - POPout - mrt) - \sum Pred$	(A.15)	gP m^{-3} d^{-1}
$\frac{dCM_{Chl}}{dt} = CM_{Chl} \cdot (synChl - degChl - mrt) - \sum Pred$	(A.16)	gChl m^{-3} d^{-1}

Table A.10: Conservation equations for NCM SVs.

conservation equation		unit
$\frac{dNCM_C}{dt} = NCM_C \cdot (Cfix + assC - Cleak - Cvoid - totR - mrt) - \sum Pred$	(A.17)	gC m^{-3} d^{-1}
$\frac{dNCM_N}{dt} = NCM_N \cdot (assN - Nout - PONout - mrt) - \sum Pred$	(A.18)	gN m^{-3} d^{-1}
$\frac{dNCM_P}{dt} = NCM_P \cdot (assP - Pout - POPout - mrt) - \sum Pred$	(A.19)	gP m^{-3} d^{-1}
$\frac{dNCM_{Chl}}{dt} = NCM_{Chl} \cdot (upChl - lossChl - mrt) - \sum Pred$	(A.20)	gChl m^{-3} d^{-1}

Appendix A.6. Model equations

Appendix A.6.1. Mathematical equations

$$normalize(x, x_{min}, x_{max}) = \frac{x - x_{min}}{x_{max} - x_{min}} \tag{A.21}$$

$$gompertz(L, b, x) = L \cdot exp(-b \cdot exp(-k \cdot x)) \tag{A.22}$$

$$monod(R, kt) = \frac{R}{R + kt} \tag{A.23}$$

Table A.11: List of all parameters for the mathematical functions listed above.

**parameter**	**parameter description**	**unit**
L	upper asymptote	dl
b	displacement along the x-axis	dl
k	growth rate of gompetz curve	dl
R	resource	dl
kt	hald-saturation constant	dl

Appendix A.6.2. Module cellular status

Table A.12: Summary of the auxiliaries in the module cellular status.

auxiliary	description	unit	origin	eq. #
Nut_iC	cellular carbon quota for nitrogen, phosphate, silica and chlorophyll-a	gNut gC^{-1}	Flynn (2001)	A.24, A.25, A.26,A.27
UmT	maximum possible growth rate at the current temperature	d^{-1}	Flynn (2020)	A.28
BR	basal respiration at the current temperature	d^{-1}	Flynn (2001)	A.30
$totR$	total respiration taking metabolic, anabolic and foraging costs into account.	gC gC^{-1} d^{-1}	Flynn (2020)	A.37
Cu	net carbon specific growth rate taking phagotrophic and phototrophic carbon sources into account	gC gC^{-1} d^{-1}	Flynn (2020)	A.38
NCu	cellular nitrogen status (1 = saturated; 0 = limited) determined using a linear relationship.	dl	modified from Flynn (2020)	A.34
PCu	cellular phosphate status (1 = saturated; 0 = limited) determined using a Gompertz curve	dl	modified from Flynn (2020)	A.35
SCu	cellular silica status (1 = saturated; 0 = limited)	dl	Flynn (2020)	A.36
$Nout$	voiding of N when exceeding maximum quota	gN gC^{-1}	Flynn (2020)	A.31
$Pout$	voiding of P when exceeding maximum quota	gP gC^{-1}	Flynn (2020)	A.32
$DOCvoid$	voiding of DOC if minimum quota is reached	gC gC^{-1}	Flynn (2020)	A.33

$$NC = \frac{protN}{protC} \tag{A.24}$$

$$PC = \frac{protP}{protC} \tag{A.25}$$

$$SC = \frac{protSi}{protC} \tag{A.26}$$

$$ChlC = \frac{protChl}{protC} \tag{A.27}$$

$$UmT = UmRT \cdot Q10^{\frac{Temp-RT}{10}} \tag{A.28}$$

$$mrt = mrtRT \cdot Q10^{\frac{Temp-RT}{10}} \tag{A.29}$$

$$BR = UmT \cdot CR \tag{A.30}$$

$$Nout = max(0.0, protN - protC \cdot NCmax) \tag{A.31}$$

$$Pout = max(0.0, protP - protC \cdot PCmax) \tag{A.32}$$

$$DOCvoid = NC < NCmin, protC - \frac{protN}{NCmin}, 0.0 \tag{A.33}$$

$$NCu = min(1.0, max(0.0, normalizeNC, NCmin, NCmax)) \tag{A.34}$$

$$PCu = gompertz(1.0, 6.0, 10.0, normalize(PC, PCmin, PCmax)) \tag{A.35}$$

$$SCu = min((monod(Si, ktSi) \cdot \frac{SCopt}{SCmin}), 1.0) \tag{A.36}$$

$$totR = (redco \cdot upNO3) + AR \cdot (upNH4 + upNO3 + assN \cdot SDA) + (assC \cdot SDA) + BR \tag{A.37}$$

$$Cu = Cfix + assC - totR \tag{A.38}$$

Appendix A.6.3. Module uptake

Table A.13: Summary of the auxiliaries in the module uptake.

auxiliary	description	unit	origin	eq. #
upP	uptake of phosphate described using the monod function and enhanced or repressed using two logistic sigmoid functions.	gP gC^{-1} d^{-1}	modified from Flynn (2020)	A.39
$upNH4$	uptake of ammonium described using the monod function and enhanced or repressed using two logistic sigmoid functions.	gN gC^{-1} d^{-1}	modified from Flynn (2020)	A.40
$upNO3$	uptake of nitrite described using the monod function and enhanced using a logistic sigmoid functions.	gN gC^{-1} d^{-1}	modified from Flynn (2020)	A.41
$upSi$	uptake of silica described using the monod function and enhanced using a logistic sigmoid functions.	gSi gC^{-1} d^{-1}	modified from Flynn (2020)	A.42

P uptake

$$APin_P = logistic(1.0, -16.0, 0.7, normalize(PC, PCmin, PCopt))$$
$$APde_P = logistic(1.0, -40.0, 0.9, normalize(PC, PCmin, PCmax))$$
$$upP_{opt} = monod(P, ktP) \cdot UmT \cdot PCopt$$
$$upP = upP_{opt} \cdot APin_P \cdot 10.0 + upP_{opt} \cdot APde_P \quad (A.39)$$

NH_4^+ uptake

$$NCPopt = ((PCu < NCu), PCoNCop + PCu \cdot (NC - PCoNCop), NC)$$
$$APin_{NH4} = logistic(1.0, -24.0, 0.85, normalize(NC, NCmin, NCPopt))$$
$$NCPopt = ((PCu < NCu), PCoNCm + PCu \cdot (NC - PCoNCm), NC)$$
$$APde_P = logistic(1.0, -40.0, 0.85, normalize(NC, NCmin, NCPmax))$$
$$upNH4_{opt} = monod(NH4, ktNH4) \cdot UmT \cdot NCopt \cdot relUm_{NH4}$$
$$upNH4 = upNH4_{opt} \cdot APin_{NH4} \cdot 3.0 + upNH4_{opt} \cdot APde_{NH4} \quad (A.40)$$

$\underline{NO_3^- \text{ uptake}}$

$$NCPm = ((PCu < NCu), PCoNCm + PCu \cdot (NC - PCoNCm), NC)$$
$$APde_{NO3} = logistic(1.0, -55.0, 0.9, normalize(NC, NCmin, NCPm))$$
$$upNO3_{opt} = monod(NO3, ktNO3) \cdot UmT \cdot NCopt \cdot relUm_{NO3}$$
$$upNO3 = upNO3_{opt} \cdot APde_{NO3} \tag{A.41}$$

Si uptake

$$APde_{Si} = logistic(1.0, -80.0, 0.95, normalize(SC, SCmin, SCmax))$$
$$upSi_{opt} = monod(Si, ktSi) \cdot UmT \cdot SCopt$$
$$upSi = upSi_{opt} \cdot APde_{Si} \tag{A.42}$$

Appendix A.6.4. Module phototrophy

Table A.14: Summary of the auxiliaries in the module phototrophy.

auxiliary	description	unit	origin	eq. #
$PSqm$	maximal attainable photosynthetic rate under optimum light (plateau of the PE-curve)	gC gC^{-1} d^{-1}	Flynn (2001)	A.43
$grossPS$	carbon fixation through photosynthesis at current light and current cellular status	gC gC^{-1} d^{-1}	Flynn (2001)	A.44
$netPS$	net carbon fixation taking leakage into account	gC gC^{-1} d^{-1}	Flynn (2001)	A.45
$synChl$	synthesis of chlorophyll-a	gChl gC d^{-1}	modified from Flynn (2020)	A.46
$degChl$	degradation of chlorophyll-a	gChl gC^{-1} d^{-1}	Flynn (2020)	A.47
$lossChl$	loss of chlorophyll-a	gChl gC^{-1} d^{-1}	Ghyoot et al. (2017)	A.48
$upChl$	uptake of chlorophyll-a from prey	gChl gC^{-1} d^{-1}	modified from Ghyoot et al. (2017)	A.49

$$PSqm = [UmT \cdot relPS \cdot (1 + PSDOC) + NCm \cdot UmT \cdot (redco + AR)] \cdot NCu + BR \tag{A.43}$$

$$X = \frac{\alpha^{Chl} \cdot ChlC \cdot PFD \cdot 24.0 \cdot 60.0 \cdot 60.0}{PSqm}$$

$$grossPS = \frac{PSqm \cdot (log(X + sqrt(1.0 + X^2)) - log(X \cdot exat + sqrt(1.0 + (X \cdot exat)^2)))}{atten} \tag{A.44}$$

$$netPS = grossPS \cdot (1.0 - PSDOC) \tag{A.45}$$

$$synChl = ChlCmax \cdot UmT \cdot NPSiCu \cdot M \cdot (1.0 - \frac{netPS}{PSqm}) \cdot$$

$$logistic(0.95, -24.0, 0.85, normalize(ChlC, ChlCmin, ChlCmax)) \tag{A.46}$$

$$degChl = (min(ChlC, ChlCmax) \cdot UmT \cdot (1.0 - NPSiCu)) \tag{A.47}$$

$$degChl_{NCM} = constant \tag{A.48}$$

$$upChl = logistic(1.0, -80, 0.93, normalize(ChlC, 0.0, ChlCmax)) \tag{A.49}$$

Appendix A.6.5. Module phagotrophy

Table A.15: Summary of the auxiliaries in the module phagotrophy.

auxiliary	description	unit	origin	eq. #
mot	motility of the protists	m s-1	Flynn & Mitra (2016)	A.50
$nrPrey$	density of prey in segment	nr cells m^{-3}	Flynn (2020)	A.51
enc	encounter rate	prey predator-1 d^{-1}	Rothschild & Osborn (1988)	A.52
$sumCP$	captured prey	gC gC^{-1} d-1	Flynn (2020)	A.54
$opAE$	assimilation efficiency	dl	Flynn (2020)	A.58
$maxIng$	maximum ingestion rate	gC gC^{-1} d-1	Flynn (2020)	A.59
$satIng$	saturation ingestion rate	gC gC^{-1} d^{-1}	Flynn (2020)	A.60
$ingC$	actual carbon ingestion rate	gC gC^{-1} d^{-1}	Flynn (2020)	A.61
$ingNut_i$	nutrient ingestion rate	gNut gC^{-1} d^{-1}	Flynn (2020)	A.62, A.63
$assC$	carbon assimilation rate	gC gC^{-1} d^{-1}	Flynn (2020)	A.64
$assNut_i$	nutrient assimilation rate	gNut gC^{-1} d^{-1}	Flynn (2020)	A.65, A.66

$$mot = 1e^{-6} \cdot (38.542 \cdot (r \cdot 2)^{0.5424}) \tag{A.50}$$

$$lightInh = sigmoidLogistic((1 - relPhag), 10.0, 1.0, PFD) + (1.0 - (1 - relPhag))$$

$$nrPrey = lightInh \cdot 1e12 \cdot \frac{preyC}{CcellPrey} \tag{A.51}$$

$$encPrey = (24.0 \cdot 60.0 \cdot 60.0) \cdot \pi \cdot (\frac{rPrey}{1E6} + \frac{rProt}{1E6})^2 \cdot nrPrey$$

$$\cdot ((vel_{prey}^2 + 3 * vel_{pred}^2 + 4 * wTurb^2) * ((vel_{pred}^2 + wTurb^2)^{-0.5})) \cdot 3.0^{-1.0} \tag{A.52}$$

$$capPrey = encPrey * PR * optCR * \frac{CcellPrey}{CcellPred} \tag{A.53}$$

$$sumCP = sum(capPrey) \tag{A.54}$$

$$ingNC = \frac{capPrey}{sumCP} \cdot \frac{preyN}{preyC} \tag{A.55}$$

$$ingPC = \frac{capPrey}{sumCP} \cdot \frac{preyP}{preyC} \tag{A.56}$$

$$stoichP = min(\frac{ingNC}{NCopt}, \frac{ingPC}{PCopt}, 1.0) \tag{A.57}$$

$$opAE = (AEo + (AEm - AEo) \cdot monod(stoichP, kAE) \cdot (1.0 + kAE)) \cdot stoichP \tag{A.58}$$

$$maxIng = \frac{UmT + BR}{1.0 - SDA} \cdot \frac{1}{opAE} opAE \tag{A.59}$$

$$satIng = maxIng \cdot monod(sumCP, \frac{maxIng}{4}) \tag{A.60}$$

$$ingC = min(ingSat, sumCP) \tag{A.61}$$

$$ingN = ingC \cdot ingNC \tag{A.62}$$

$$ingP = ingC \cdot ingPC \tag{A.63}$$

$$assC = ingC \cdot opAE \tag{A.64}$$

$$assN = assC \cdot NCopt \tag{A.65}$$

$$assP = assC \cdot PCopt \tag{A.66}$$

Appendix B. Boundary forcings

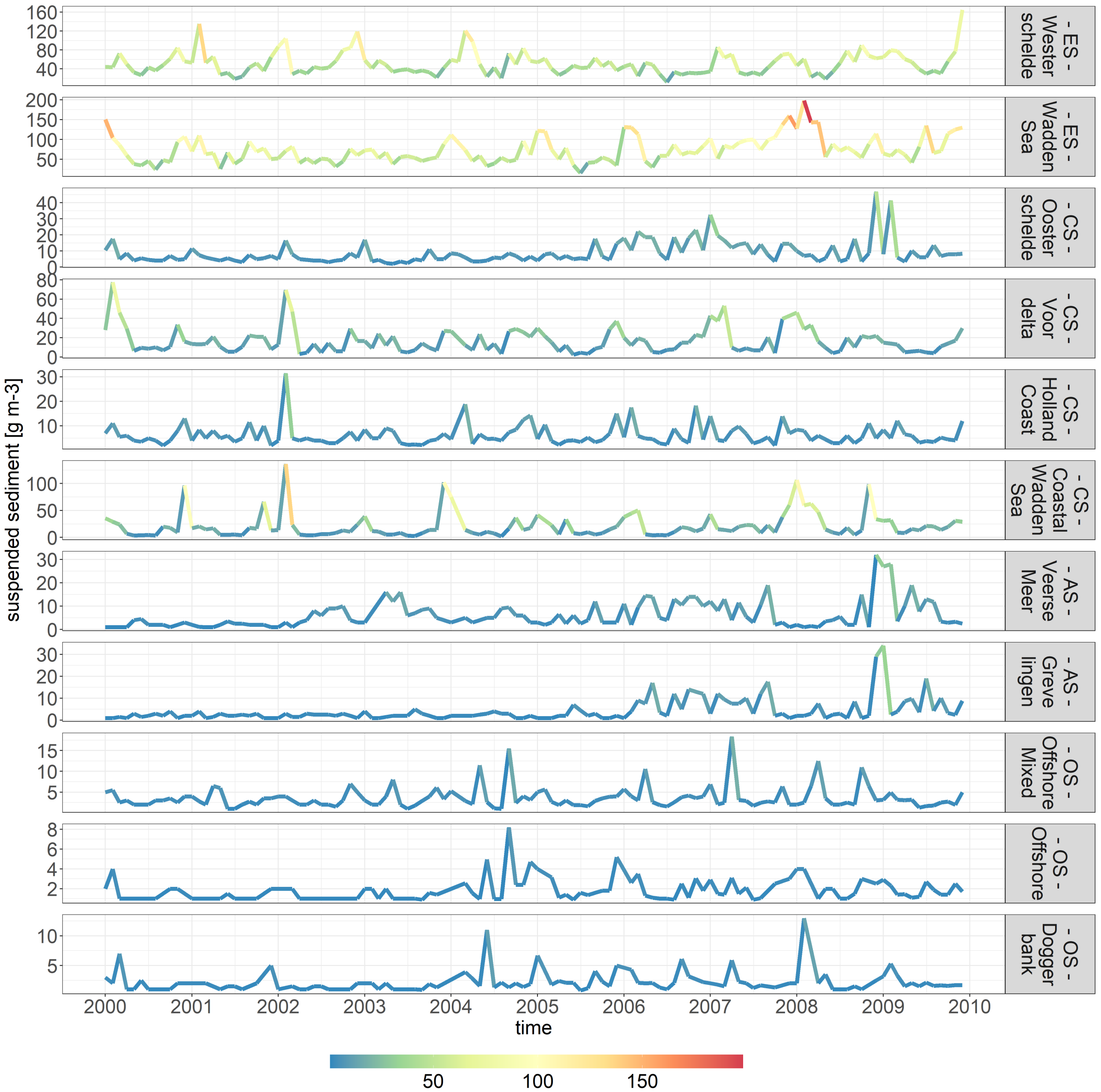

Figure B.1: Boundary transport of suspended sediment.

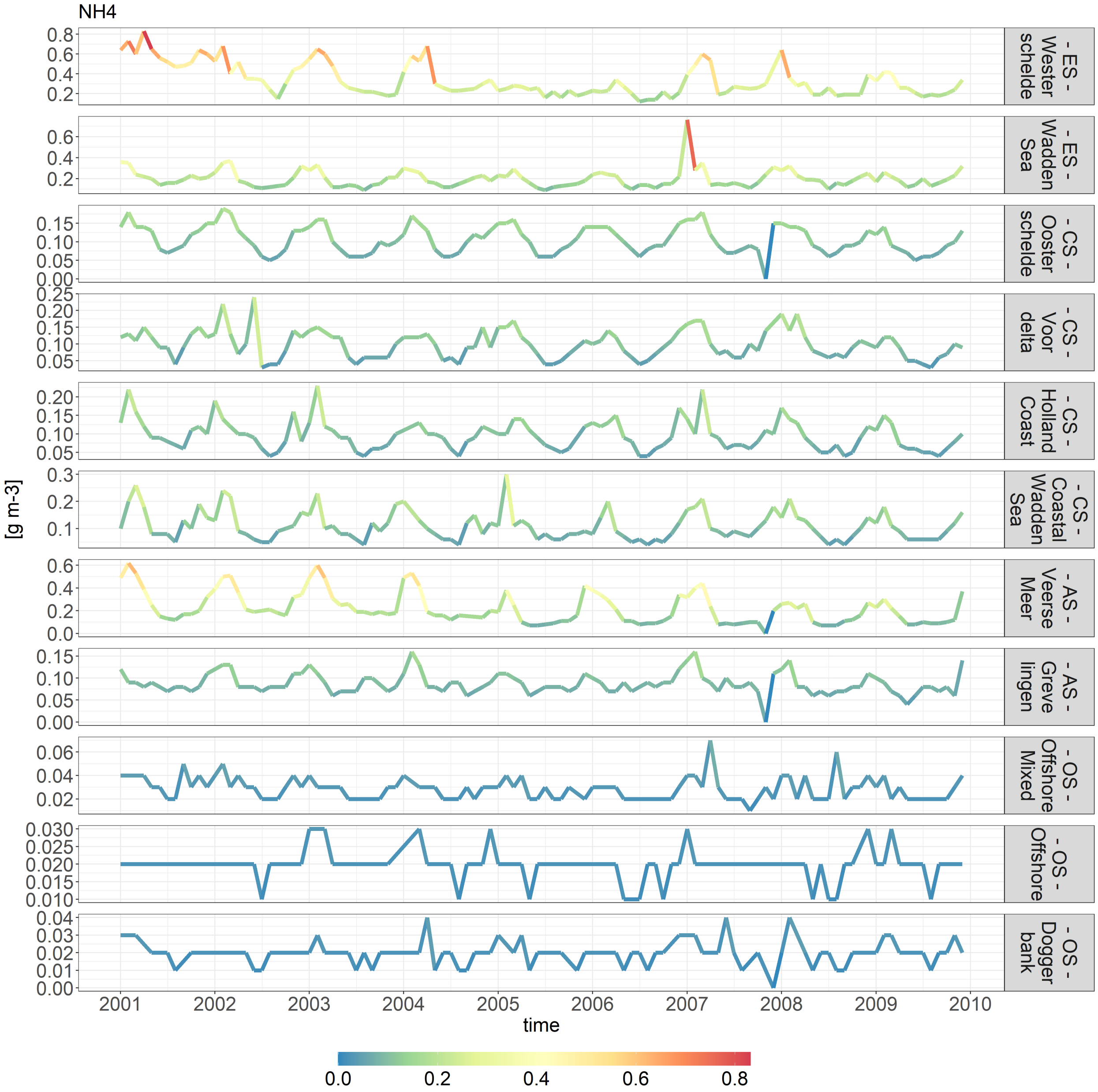

Figure B.2: Boundary transport of ammonium.

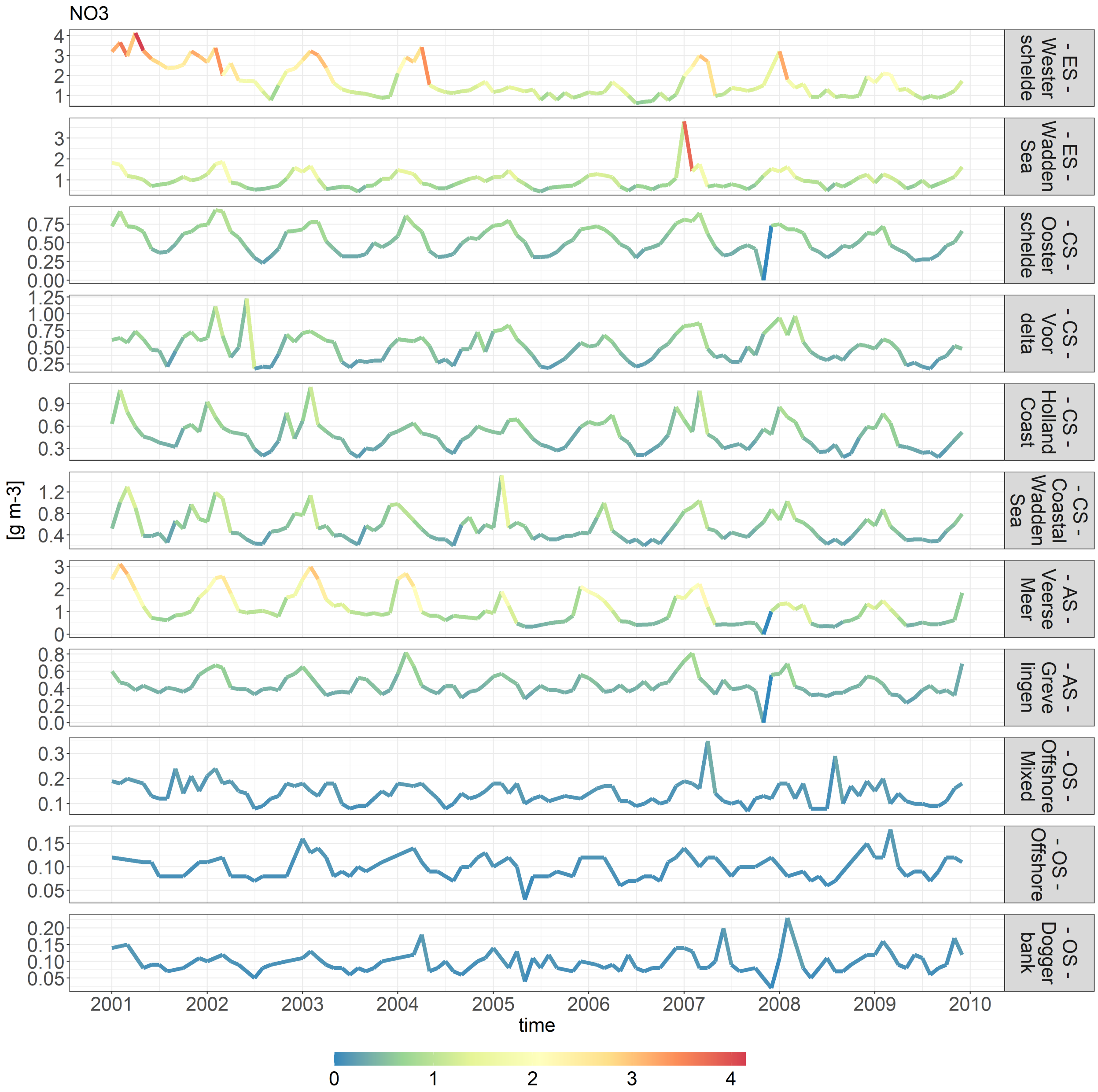

Figure B.3: Boundary transport of nitrate.

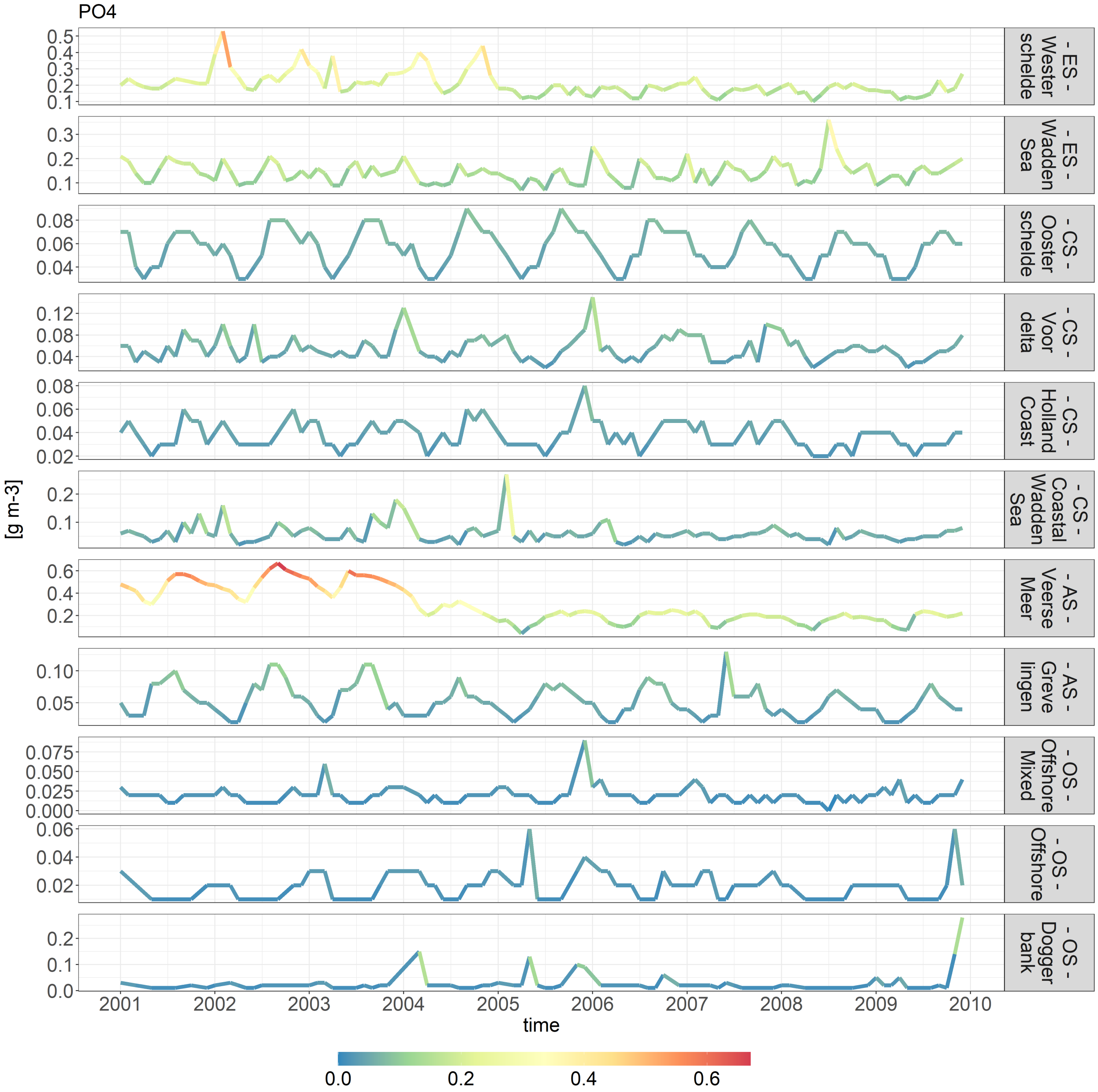

Figure B.4: Boundary transport of phosphorus.

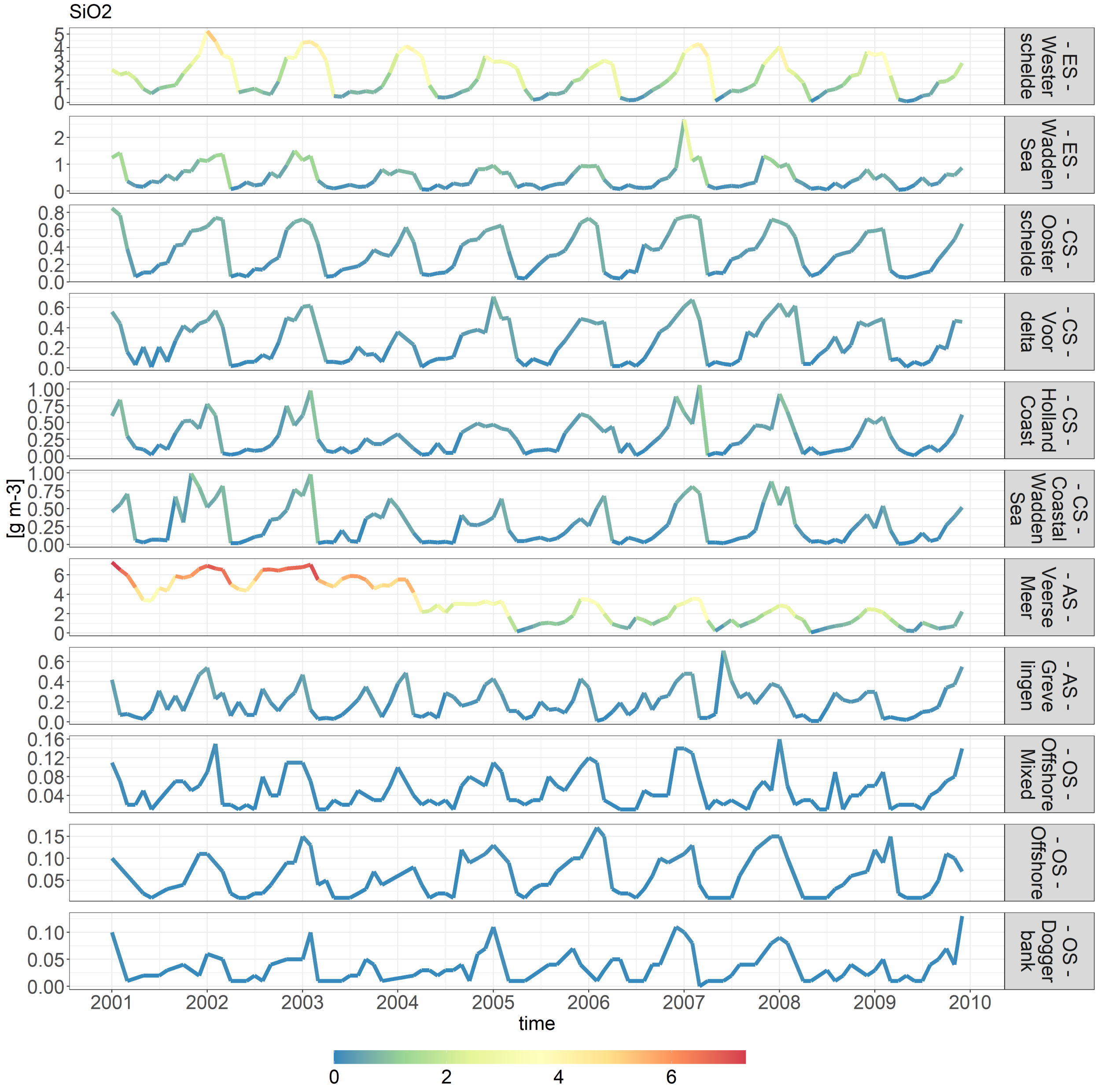

Figure B.5: Boundary transport of silica.

Appendix C. Model forcings

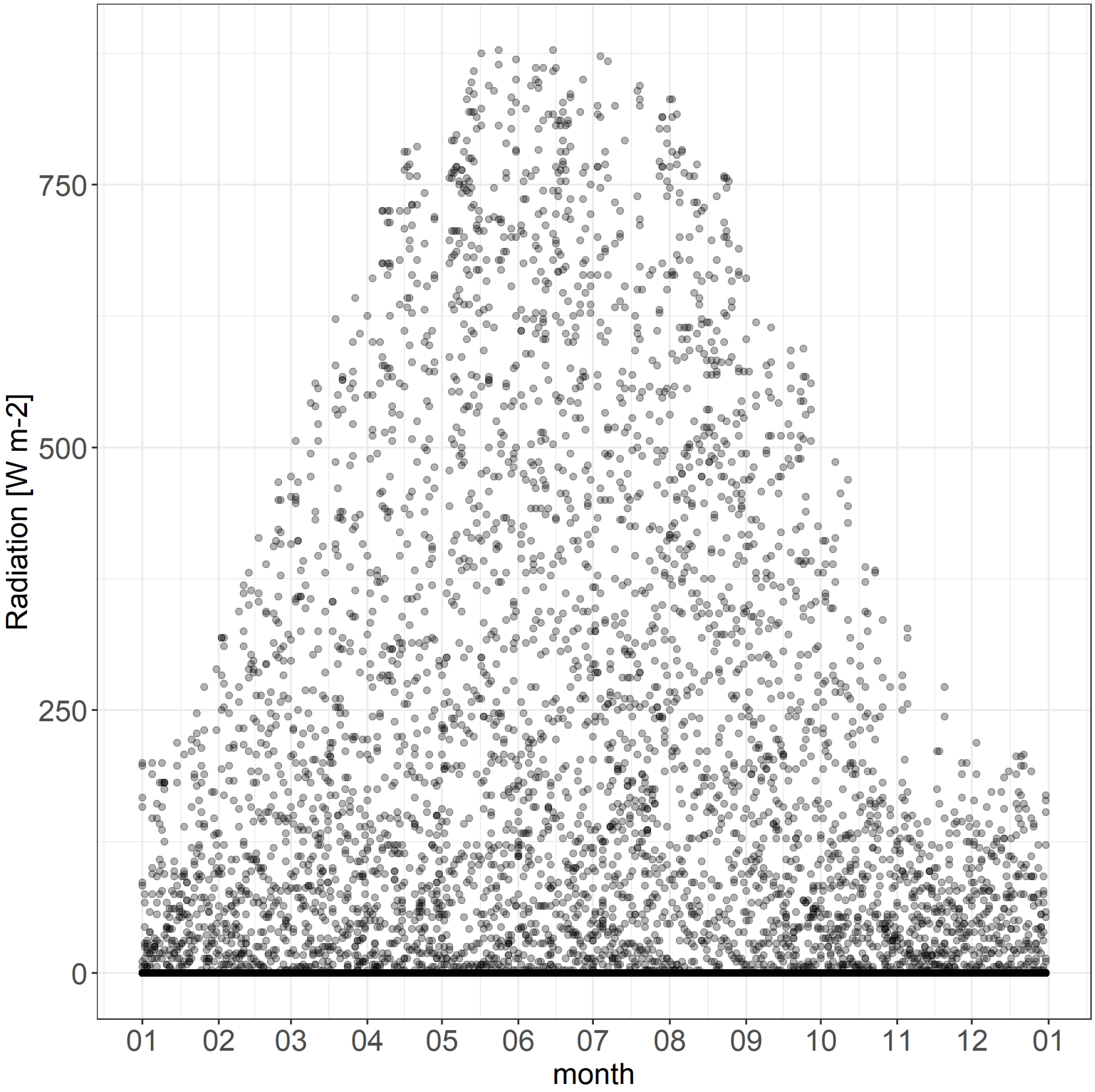

Figure C.1: Forced hourly radiation. Data was retrieved from the Royal Netherlands Meterorological Institute (KNMI) for the year 2019 for the sampling station de Kooy.

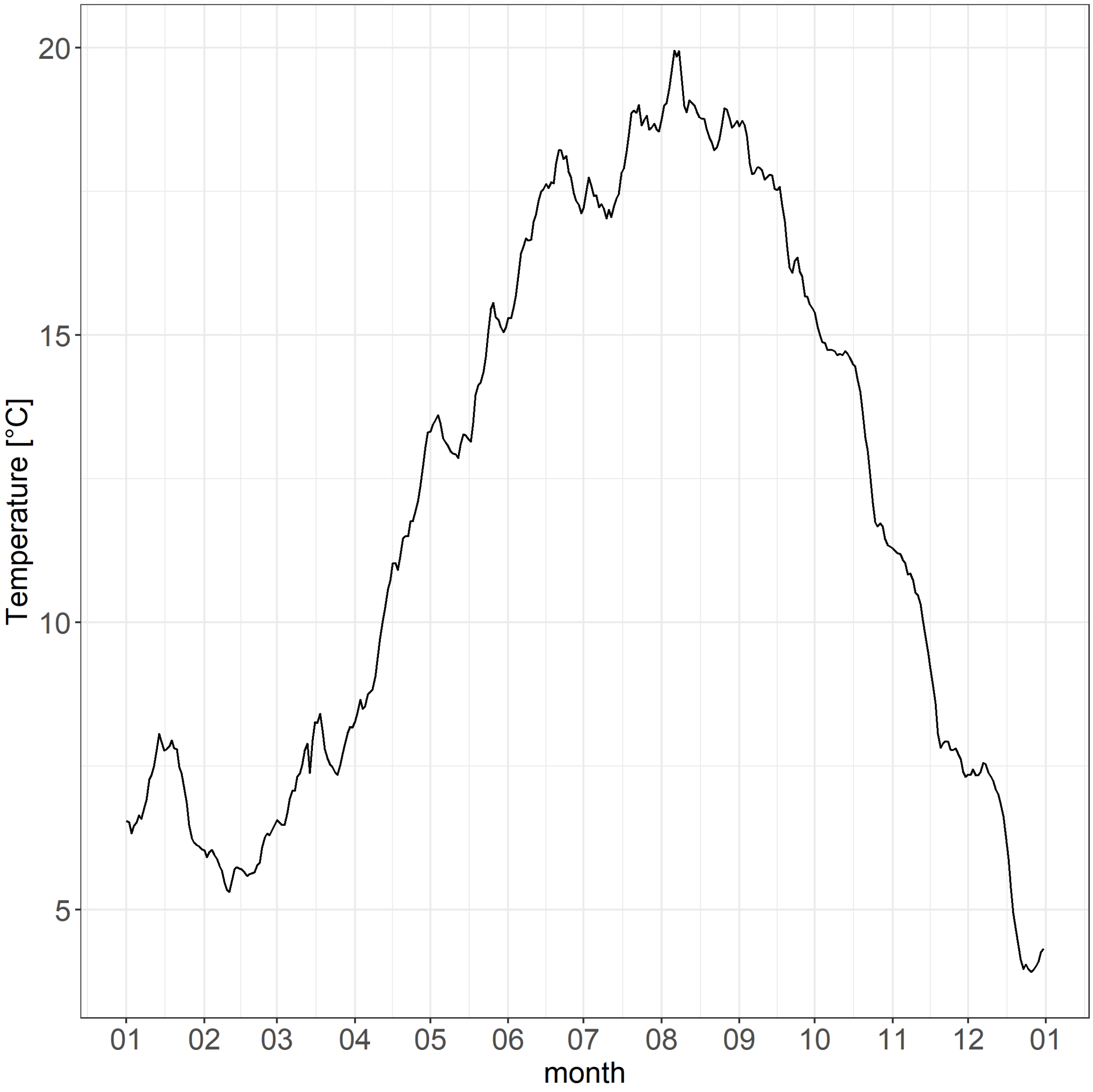

Figure C.2: Forced daily temperature. Data was retrieved from the 3D model Merzandwinning for the year 2014.

Appendix D. Box model

Appendix D.1. Box model set-up

The box model is used to demonstrate growth and competition between the five PFTs, diatoms, green algae, protozooplankton, CMs and NCMs. Only the PROTIST module was activated for the box model. It was run for 60 days with a timestep of 3 min and an output timestep of 2 h. The box model set-up mimics a batch culture with an initial nutrient supply, a day-night cycle of 12:8 h, no remineralization of particulate organics and no additional mortality apart from grazing.

All PFTs had a growth rate of 0.81 d^{-1}. Mortality was deactivated. The dimensionless parameter $relPS$ (the ratio of photosynthesis rate to maximum growth rate) was set to 2 for the primarily phototrophic organisms and to 0.5 for NCMs. Stoecker et al. (1988) showed that NCMs ingest less prey in the dark, so the ingestion of prey by NCMs is slightly light dependent (0.7). As there were no NCMs present in that dataset, the size for NCMs was set to 40 μm ESD to mimic an average *Strombidium*. The parameters for the other PFTs were set according to the table in A.2.

Appendix D.2. Box model results

Figure D.1 displays a 60 days run of the box model mimicking a batch culture. It displays the carbon biomass SVs (fig. D.1a), the nutrient SVs (figs. D.1b, D.1d, D.1f) as well as the assimilation rates (fig. D.1c) and carbon fixation rates (fig. D.1e). These plots demonstrate that PROTIST responds as would be expected.

The primarily phototrophic organisms bloom first with diatoms displaying the highest biomass peak (see fig. D.1a). All primarily phototrophic organisms initially display high rates of carbon fixation, which respond to the day-night cycle (see fig. D.1e), but those carbon fixation rates decline as the macro nutrients become limiting. Macronutrients become limiting after approximately 15 days (see figs. D.1b, D.1d, D.1f) leading to a decline of the diatoms and green algae. As the diatoms remain silica limited (see fig. D.1f), their biomass as well as carbon fixation rates remain low compared to green algae and CMs which display an increase of biomass as ammonium and phosphate become available again through voiding.

Fig. D.1c shows that all organisms capable of phagotrophy are prey limited as their assimilation rates closely follow their preys' biomass curves. The assimilation of prey by NCMs is also reflected in their carbon fixation rates, which are initially very low but increase as the NCM assimilated prey and retains their chloroplasts (see fig. D.1e).

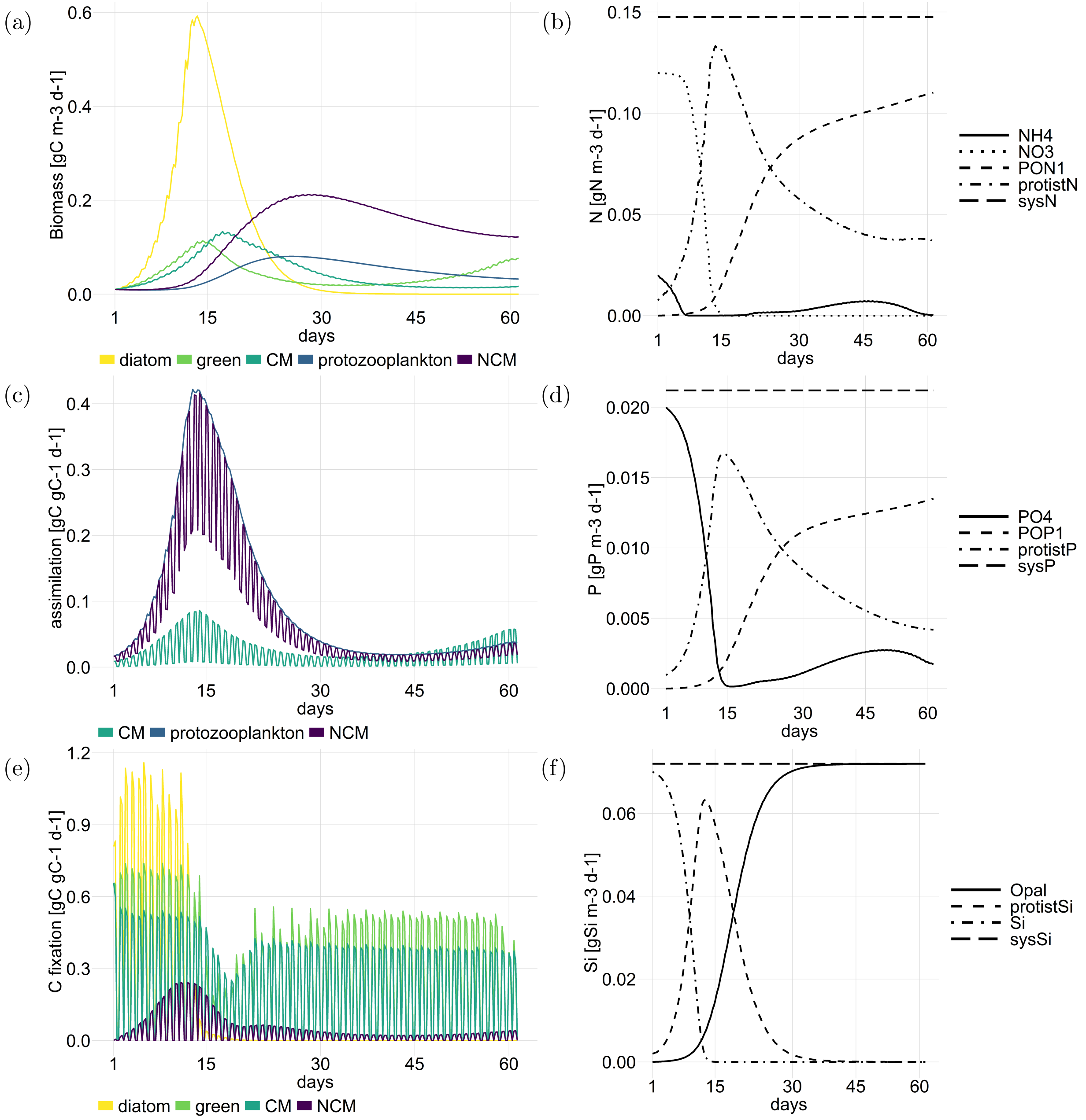

Figure D.1: Graphs displaying a) the carbon biomass per PFT, b) all SV related to nitrogen, c) assimilation of prey, d) all SV related to phosphate, e) carbon fixation and f) all SV related to silica.

Appendix E. Normalized standard deviation

Table E.1: Normalized standard deviations of the abiotic factor included in the sensitivity analysis

	$\overline{sd_x}$
NH_4^+	0.95
NO_3^-	0.95
PO_4^{3-}	1.24
SiO_2	1.78
suspended sediment	1.46
