# Supplementary material for: Corrigendum to ‘Modeling mixoplankton along the biogeochemical gradient of the Southern North Sea’ Ecological Modelling 458 (2021) 0304-3800/109690
Source: Ecol Modell. 2023 Jul;481:110374. doi: 10.1016/j.ecolmodel.2023.110374 (PMC10172845; doi:10.1016/j.ecolmodel.2023.110374)
Supplement: Supplementary file 1 [file mmc1.docx]

Appendix: PROTIST module description

## Lisa K. Schneider^a,b^, Nathalie Gypens^b^, Tineke A. Troost^a^, Willem Stolte^a,∗^

*^a^Deltares, Boussinesqweg 1, 2629 HV Delft, South-Holland, The Netherlands*

*^b^Universit´e Libre de Bruxelles, Laboratoire d’Ecologie des Syst`emes Aquatiques, CP221, Boulevard du Triomphe, B-1050, Belgium*

# Appendix A. General information

## The module PROTIST is based on model equations from [Flynn](#_bookmark51) [(200](#_bookmark51)1), [Flynn & Mitra](#_bookmark53) [(2009)](#_bookmark53) and [Flynn](#_bookmark52) [(2021).](#_bookmark52) Those model equations have recently been summarized in a model called SAPPM (Switchable Acclimative Protist Plankton - Model) that can be used

5 to describe protozooplankton, phytoplankton (as diatoms and non-diatoms) and constitutive mixoplankton [(Flynn, 2021).](#_bookmark52)

## For the module PROTIST, the SAPPM structure was implemented. The SAPPM equations were adapted to work in a 3D modelling software environment. To separate between the stand-alone version of the model and the 3D implementation, the name

10 PROTIST was applied. The module PROTIST can simultaneously model protozooplankton, phytoplankton (as diatoms and non-diatoms) and constitutive mixoplankton. The code for PROTIST was written in Fortran and can be downloaded at <https://github.com/lkschn/PROTISTcode.git>. The authors recommend to look at the Fortran code as it give the best overview of the PROTIST module structure. As of spring

## 15 2022, the PROTIST module code and description can also be retrieved from the official release of the 3D modelling software D-Water quality [(Deltares, 202](#_bookmark50)1).

In the following the PROTIST module state variable, parameters, auxiliaries, equations and their origin will be listed.

^∗^Corresponding author

*Email address:* [Willem.Stolte@deltares.nl](mailto:Willem.Stolte@deltares.nl) (Willem Stolte)

*Preprint submitted to Journal of Ecological Modelling February 3, 2022*

# Appendix B. Model description

20 *Appendix B.1. Model SV*

#### Table B.1: List of all model state variables, their description, unit and initial value. Values labeled with a * differ for each location class.

| **state**  **variable** | **state variable description** | **unit** | **value** |
| --- | --- | --- | --- |
| PO4 | initial DIP | gP m^−3^ | * |
| NH4 | initial NH^+^  4 | gN m^−3^ | * |
| NO3 | initial NO^−^_3_ | gN m^−3^ | * |
| Si | initial Si | gSi m^−3^ | * |
| Opal | Opal-Si | gSi m^−3^ | * |
| POC1 | POC1 (fast decomposing fraction) | gC m^−3^ | 0.0 |
| PON1 | PON1 (fast decomposing fraction) | gN m^−3^ | 0.0 |
| POP1 | POP1 (fast decomposing fraction) | gP m^−3^ | 0.0 |
| DOClab | labile DOC | gC m^−3^ | 0.0 |
| OXY | oxygen | gO2 m^−3^ | 0.0 |
| greenC | green C-biomass | gC m^−3^ | 0.01 |
| greenChl | green Chl-biomass | gChl m^−3^ | 0.0002 |
| greenN | green N-biomass | gN m^−3^ | 0.0015 |
| greenP | green P-biomass | gP m^−3^ | 0.00024 |
| diatC | diatom C-biomass | gC m^−3^ | 0.01 |
| diatChl | diatom Chl-biomass | gChl m^−3^ | 0.0002 |
| diatN | diatom N-biomass | gN m^−3^ | 0.0015 |
| diatP | diatom P-biomass | gP m^−3^ | 0.00024 |
| diatSi | diatom Si-biomass | gSi m^−3^ | 0.002 |
| cmC | CM C-biomass | gC m^−3^ | 0.01 |
| cmChl | CM Chl-biomass | gChl m^−3^ | 0.0002 |
| cmN | CM N-biomass | gN m^−3^ | 0.0015 |
| cmP | CM P-biomass | gP m^−3^ | 0.00024 |
| zooC | protozooplankton C-biomass | gC m^−3^ | 0.01 |
| zooN | protozooplankton N-biomass | gN m^−3^ | 0.0015 |

*Continued on next page*

Table B.1 – *Continued from previous page*

| **state**  **variable** | **state variable description** | **unit** | **value** |
| --- | --- | --- | --- |
| zooP | protozooplankton P-biomass | gP m^−3^ | 0.00024 |
| ncmC | NCM C-biomass | gC m^−3^ | 0.01 |
| ncmChl | NCM Chl-biomass | gChl m^−3^ | 0.0002 |
| ncmN | NCM N-biomass | gN m^−3^ | 0.0015 |
| ncmP | NCM P-biomass | gP m^−3^ | 0.00024 |

*Appendix B.2. Model parameters*

#### Table B.2: List of all model parameters for a generic PFT, their description, unit and default value. Values labeled with a * can be found in table [B.3.](#_bookmark1) The abbreviation dl is short for dimensionless.

| **parameter** | **parameter description** | **unit** | **value** |
| --- | --- | --- | --- |
| AEm | maximum assimilation efficiency (AE) | dl | 0.6 |
| AEo | minimum AE | dl | 0.3 |
| alpha | alpha for photosynthesis in protist | gC gChl^−1^ m^2^  umol^−1^ photon | * |
| abcChl | light absorbance coefficient for chlorophyll | m^2^ gChl^−1^ | 20 |
| Ccell | C content of protist cell | pgC cell^−1^ | * |
| ChlCm | maximum cellular Chl:C ratio | gChl gC^−1^ | * |
| ChlCo | minimum cellular Chl:C ratio | gChl gC^−1^ | 0.001 |
| CR | catabolic respiration quotient | dl | 0.05 |
| degChl | Chl degradation | d−1 | 0.72 |
| FrAut | fraction of mortality to autolysis | dl | 0.3 |
| FrDet | fraction of mortality to detritus | dl | 0.7 |
| kAE | control of AE in response to prey quality | dl | 1000 |
| KtNH4 | Kt for NH^+^ transport  4 | gN m^−3^ | 0.007 |
| KtNO3 | Kt for NO^−^_3_ transport | gN m^−3^ | 0.007 |
| KtP | Kt for DIP transport | gP m^−3^ | 0.031 |
| KtSi | Kt for DiSi transport | gSi m^−3^ | 0.028 |
| MrtRT | mortality at reference temperature | dl | * |
| Mphoto | acclimation rate to light | dl | 0.5 |
| NCmax | N:C that totally represses NH^+^ transport  4 | gN gC^−1^ | * |
| NCmin | minimum N-quota | gN gC^−1^ | * |
| NCopt | N:C for growth under optimal conditions | gN gC^−1^ | * |
| NO3Cmax | N:C that totally represses NO^−^3 transport | gN gC^−1^ | * |
| NO3Copt | N:C for growth on NO_3_^−^ under optimal conditions | gN gC^−1^ | * |
| optCR | proportion of prey captured by starved Zoo | dl | 0.1 |
| PCmax | PC maximum quota | gP gC^−1^ | * |
| PCmin | PC minimum quota | gP gC^−1^ | * |

*Continued on next page*

Table B.2 – *Continued from previous page*

| **parameter** | **parameter description** | **unit** | **value** |
| --- | --- | --- | --- |
| PCoNCm | maximum NC when PC is minimum (PCu = 0) | gN gC^−1^ | * |
| PCoNCop | optimum NC when PC is minimum (PCu = 0) | gN gC^−1^ | * |
| PCopt | PC optimum quota | gP gC^−1^ | * |
| PSDOC | proportion of current PS being leaked as DOC | dl | 0.1 |
| Q10 | Q10 for UmRT | dl | * |
| r | radius of nutrient repleted protist cell | um | * |
| redco | C respired to support nitrate reduction for NH^+^  4 | gC gN-1 | 1.71 |
| relPhag | relative phagotrophy in night:day | dl | * |
| relPS | relative PSmax:Umax on phototrophy | dl | * |
| ReUmNH4 | max. growth rate supported by NH^+^:Umax  4 | dl | 0.9 |
| ReUmNO3 | max. growth rate supported by NO^−^3 :Umax | dl | 0.8 |
| RT | reference temperature for UmRT | deg C | 10 |
| SDA | specific dynamic action | dl | 0.3 |
| UmRT | maximum growth rate at reference T | d−1 | * |
| SCmax | absolute maximum Si:C (diatom) | gSi gC^−1^ | 0.2 |
| SCmin | minimum Si:C (diatom) | gSi gC^−1^ | 0.02 |
| SCopt | optimum Si:C for (diatom) growth | gSi gC^−1^ | 0.1 |
| AR | anabolic respiration cost in terms of C | gC gN^−1^ d^−1^ | 1.5 |
| M | scalar for controlling photoacclimation rate | dl | 0.5 |

#### Table B.3: Summary of the PFT specific parameters established through literature as stated in text. Note that the protozooplankton mortality (marked with *) uses a quadratic closure function, while the phytoplankton and CM mortality use a linear mortality function.

Furthermore, the values for NCmin and PCmin in the 1D-V model runs are unintentionally set to unrealistically high values. For more realistic representation of the protist physiology, lower NCmin and PCmin values should be chosen (e.g. 0.05 for NCmin and 0.005 for PCmin).

| parameter | units | | diatom | green  algae | CM | protozoo-  plankton | origin | |
| --- | --- | --- | --- | --- | --- | --- | --- | --- |
| ESD | *µ*m | | 24.0 | 10.0 | 18.0 | 40.0 | [Schneider et al. (2020)](#_bookmark63) | |
| Ccell | pgC cell^−1^ | | 909.19 | 77.20 | 404.29 | 3833.51 | calculated [Menden-Deuer](#_bookmark61)  [Lessard (2000)](#_bookmark61) | using  [&](#_bookmark61) |
| ChlCmax | gChl gC^−1^ | | 0.058 | 0.033 | 0.021 | - | [Geider et al. (1997)](#_bookmark55) | |
| *αChl* | gC gChl^−1^ m^2^  umol^−1^ photon | | 9.5e-6 | 7e-6 | 7e-6 | - | [Geider et al. (1997)](#_bookmark55) | |
| NCmin | gN | gC−1 | 0.11 | 0.14 | 0.09 | 0.05 | [Leonardos &](#_bookmark59)  [(2004)](#_bookmark59) | [Geider](#_bookmark59) |
| NCopt | gN | gC−1 | 0.15 | 0.17 | 0.12 | 0.15 | [Leonardos &](#_bookmark59)  [(2004)](#_bookmark59) | [Geider](#_bookmark59) |
| NCmax | gN | gC−1 | 0.2 | 0.2 | 0.2 | 0.2 | [Leonardos &](#_bookmark59)  [(2004)](#_bookmark59) | [Geider](#_bookmark59) |
| PCminNCopt | gN | gC−1 | 0.12 | 0.15 | 0.1 | - | calibrated using [Flynn](#_bookmark52)  [(2021)](#_bookmark52) | |
| PCminNCmax | gN | gC−1 | 0.13 | 0.16 | 0.11 | - | calibrated using [Flynn](#_bookmark52)  [(2021)](#_bookmark52) | |
| NO3Copt | gN | gC−1 | 0.14 | 0.16 | 0.11 | - | based on [Leonardos &](#_bookmark59)  [Geider (2004)](#_bookmark59) | |
| NO3Cmax | gN | gC−1 | 0.16 | 0.18 | 0.13 | - | based on [Leonardos &](#_bookmark59)  [Geider (2004)](#_bookmark59) | |
| PCmin | gP | gC−1 | 0.009 | 0.02 | 0.006 | 0.005 | [Leonardos &](#_bookmark59)  [(2004)](#_bookmark59) | [Geider](#_bookmark59) |

*Continued on next page*

Table B.3 – *Continued from previous page*

| parameter | | units | diatom | green  algae | CM | protozoo-  plankton | origin | |
| --- | --- | --- | --- | --- | --- | --- | --- | --- |
| PCopt | | gP gC^−1^ | 0.014 | 0.028 | 0.012 | 0.024 | [Leonardos &](#_bookmark59)  [(2004)](#_bookmark59) | [Geider](#_bookmark59) |
| PCmax | | gP gC^−1^ | 0.029 | 0.036 | 0.028 | 0.05 | [Leonardos &](#_bookmark59)  [(2004)](#_bookmark59) | [Geider](#_bookmark59) |
| relPS | | dl | 2 | 2 | 2 | - | [Geider et al. (1998)](#_bookmark56) | |
| relPhag | | dl | - | - | 0.1 | 1 | [Skovgaard](#_bookmark64) [(1996);](#_bookmark64) [Li](#_bookmark60) [et al.](#_bookmark60) [(1999);](#_bookmark60) [Adolf](#_bookmark47) [et al. (2006);](#_bookmark47) [Anderson](#_bookmark48)  [et al. (2018)](#_bookmark48) | |
| PR diatom | | dl | - | - | - | 1 | information from  [Jeong et al. (2010)](#_bookmark58) | |
| PR  algae | green | dl | - | - | 1 | 1 | information from  [Jeong et al. (2010)](#_bookmark58) | |
| PR CM | | dl | - | - | - | 1 | information from  [Jeong et al. (2010)](#_bookmark58) | |
| sed | | m d−1 | 0.38 | - | - | - | Stokes law | |
| mrt | | d−1 | 0.07 | 0.07 | 0.07 | 0.007 * | [Blauw et al. (2009)](#_bookmark49) | |

### 25 Appendix B.3. Model auxiliaries

#### Table B.4: List of all model auxiliaries for a generic PFT, their description and unit.

| **auxiliary** | **auxiliary description** | **unit** |
| --- | --- | --- |
| NC | cellular nitrogen:carbon ratio | gN gC^−1^ |
| PC | cellular phosphate:carbon ratio | gP gC^−1^ |
| SC | cellular silica:carbon ratio | gSi gC^−1^ |
| ChlC | cellular chlorphyll:carbon ratio | gChl gC^−1^ |
| UmT | temperature dependent maximum growth rate | gC gC^−1^ d^−1^ |
| BR | temperature dependent basal respiration rate | gC gC^−1^ d^−1^ |
| NCu | cellular nitrogen status | dl |
| PCu | cellular phosphate status | dl |
| SCu | cellular silica status | dl |
| NPCu | Liebig nutrient limitation | dl |
| mot | motility of the protist | m s-1 |
| upP | uptake rate of phosphate | gP gC^−1^ d^−1^ |
| upNH4 | uptake rate of ammonium | gN gC^−1^ d^−1^ |
| upNO3 | uptake rate of nitrate | gN gC^−1^ d^−1^ |
| upSi | uptake rate of silica | gSi gC^−1^ d^−1^ |
| upChl | uptake rate of chlorphyll | gChl gC^−1^ d^−1^ |
| PSqm | maximum photosynthetic rate | gC gC^−1^ d^−1^ |
| PS | gross photosynthetic rate | gC gC^−1^ d^−1^ |
| Cfix | net photosynthetic rate | gC gC^−1^ d^−1^ |
| synChl | synthesis rate of chlorophyll-a | gChl gC^−1^ d^−1^ |
| degChl | degradation rate of chlorphyll | gChl gC^−1^ d^−1^ |
| sumCP | rate of all potential prey captures | gC gC^−1^ d^−1^ |
| ingNC | rate of captured nitrogen:carbon | gN gC^−1^ d^−1^ |
| ingPC | rate of captured phosphate:carbon | gP gC^−1^ d^−1^ |
| ppNC | ratio of captured prey nitrogen: predator nitrogen | dl |
| ppPC | ratio of captured prey nitrogen: predator nitrogen | dl |
| stoichP | limiting nutrient in prey | dl |
| opAE | assimilation efficiency of predator | dl |
| maxIng | maximum ingestion rate | gC gC^−1^ d^−1^ |

*Continued on next page*

Table B.4 – *Continued from previous page*

| **auxiliary** | **auxiliary description** | **unit** |
| --- | --- | --- |
| ingSat | satiation ingestion rate | gC gC^−1^ d^−1^ |
| ingC | ingestion rate of prey carbon | gC gC^−1^ d^−1^ |
| assC | assimilation rate of prey carbon | gC gC^−1^ d^−1^ |
| ingN | ingestion rate of prey nitrogen | gN gC^−1^ d^−1^ |
| ingP | ingestion rate of prey phosphate | gP gC^−1^ d^−1^ |
| assN | assimilation rate of prey nitrogen | gN gC^−1^ d^−1^ |
| assP | assimilation rate of prey phosphate | gP gC^−1^ d^−1^ |
| totR | total respiration rate | gC gC^−1^ d^−1^ |
| Cu | carbon-specific growth rate | gC gC^−1^ d^−1^ |
| mrt | mortality rate | gC gC^−1^ d^−1^ |
| lInh | light inhibition factor | dl |
| capPrey | potential C-specific capture of prey | gC gC^−1^ d^−1^ |
| exat | extinction by phytoplankton | m−1 |

### Appendix B.4. Model fluxes

#### Table B.5: List of all model fluxes for a generic PFT, their description and unit.

| **flux** | **flux description** | **unit** |
| --- | --- | --- |
| dNH4up | uptake of NH^+^ into algal biomass  4 | gN m−3 d−1 |
| dNO3up | uptake of NO3− into algal biomass | gN m−3 d−1 |
| dPup | uptake of PO^3−^ into algal biomass  4 | gP m−3 d−1 |
| dSiup | uptake of Si into algal biomass | gSi m^−3^ d^−1^ |
| dCfix | contribution to biomass growth from C-fixation | gC m−3 d−1 |
| dChlsyn | synthesis Chl rate of change | gChl m^−3^ d^−1^ |
| dChldeg | degradation Chl rate of change | gChl m^−3^ d^−1^ |
| dChlup | acquistion of prey Chl by NCM | gChl m^−3^ d^−1^ |
| dCresp | total respiration rate | gC m−3 d−1 |
| dCleak | release of DOC | gC m−3 d−1 |
| dCvoid | voiding of C as DOC if NC falls below NCmin | gC m−3 d−1 |
| dNH4out | NH^+^ release by regeneration  4 | gN −3 d−1 |
| dPout | PO^3−^ release by regeneration  4 | gP −3 d−1 |
| dCeat | assimilation of C from prey | gC m−3 d−1 |
| dNeat | assimilation of N from prey | gN m−3 d−1 |
| dPeat | assimilation of P from prey | gP m−3 d−1 |
| dPOCout | rate of voiding of C as particulates | gC m−3 d−1 |
| dPONout | rate of voiding of N as particulates | gN m−3 d−1 |
| dPOPout | rate of voiding of P as particulates | gP m−3 d−1 |
| dAutC | protist-C mortality through Autolysis | gC m−3 d−1 |
| dDetC | protist-C mortality through Detritus | gC m−3 d−1 |
| dAutN | protist-N mortality through Autolysis | gN m−3 d−1 |
| dDetN | protist-N mortality through Detritus | gN m−3 d−1 |
| dAutP | protist-P mortality through Autolysis | gP m−3 d−1 |
| dDetP | protist-P mortality through Detritus | gP m−3 d−1 |
| dAutSi | protist-Si mortality through Autolysis | gSi m^−3^ d^−1^ |
| dDetSi | protist-Si mortality through Detritus | gSi m^−3^ d^−1^ |
| dAutChl | protist-Chl mortality through Autolysis | gChl m^−3^ d^−1^ |
| dDetChl | protist-Chl mortality through Detritus | gChl m^−3^ d^−1^ |

*Continued on next page*

Table B.5 – *Continued from previous page*

| **flux** | **flux description** | **unit** |
| --- | --- | --- |
| dD1C | mortality of prey i through predator j | gC m−3 d−1 |
| dD1Chl | mortality of prey i through predator j | gChl m^−3^ d^−1^ |
| dD1N | mortality of prey i through predator j | gN m−3 d−1 |
| dD1P | mortality of prey i through predator j | gP m−3 d−1 |
| dD1Si | mortality of prey i through predator j | gSi m^−3^ d^−1^ |

#### Table B.6: Generic protist state variable names used in the following tables and equations.

| generic SV | generic SV description | unit |
| --- | --- | --- |
| protC | protist carbon biomass | gC m^−3^ |
| protN | protist nitrogen biomass | gN m^−3^ |
| protP | protist phosphorus biomass | gP m^−3^ |
| protChl | protist chlorophyll biomass | gChl m^−3^ |
| protSi | protist silica biomass | gSi m^−3^ |

*dNH*4*up* = *protC · upNH*4 (B.1)

*dNO*3*up* = *protC · upNO*3 (B.2)

*dPup* = *protC · upP* (B.3)

*dSiup* = *protC · upSi* (B.4)

*dCfix* = *protC · Cfix* (B.5)

*dChlsyn* = *protC · synChl* (B.6)

*dChldeg* = *protC · degChl or protC · degChl_NCM_* (B.7)

*capPrey dChlup* = *protC ·* (*ingC · sumCP* ) *·*

*preyChl*

*preyC · upChl* (B.8)

*dCresp* = *protC · totR* (B.9)

*dCleak* = *protC ·* (*PS − Cfix*) (B.10)

*dCvoid* = *protC − protN/NCmin* (B.11)

*dNH*4*out* = *max*(0*.*0*, protN − protC · NCmax*) (B.12)

*dPout* = *max*(0*.*0*, protP − protC · PCmax*) (B.13)

*dCeat* = *protC · assC* (B.14)

*dNeat* = *protC · assN* (B.15)

*dPeat* = *protC · assP* (B.16)

*dPOCout* = *protC ·* (*ingC − assC*) (B.17)

*dPONout* = *protC ·* (*ingN − assN* ) (B.18)

*dPOPout* = *protC ·* (*ingP − assP* ) (B.19)

*dAutC* = *protC · mrt · FrAut* (B.20)

*dDetC* = *protC · mrt · FrDet* (B.21)

*dAutN* = *protN · mrt · FrAut* (B.22)

*dDetN* = *protN · mrt · FrDet* (B.23)

*dAutP* = *protP · mrt · FrAut* (B.24)

*dDetP* = *protP · mrt · FrDet* (B.25)

*dAutSi* = *protSi · mrt · FrAut* (B.26)

*dDetSi* = *protSi · mrt · FrDet* (B.27)

(B.28)

*dAutChl* = *protChl · mrt · FrAut* (B.29)

*dDetChl* = *protChl · mrt · FrDet* (B.30)

*capPrey*

*dD*1*C* = *protC ·* (*ingC · sumCP* ) (B.31)

*dD*1*Chl* = *dD*1*C ·* (*preyChl/preyC*) (B.32)

*dD*1*N* = *dD*1*C ·* (*preyN/preyC*) (B.33)

*dD*1*P* = *dD*1*C ·* (*preyP/preyC*) (B.34)

*dD*1*Si* = *dD*1*C ·* (*preySi/preyC*) (B.35)

(B.36)

### Appendix B.5. Conservation equations

#### Table B.7: Conservation equations for diatom SVs.

conservation equation unit

*dDiatC* = *dCfix* − *dCleak* − *dCvoid* − *dCresp* − *dAutC* − *dDetC* − Σ *Pred* (B.37) gC m*^−^*^3^ d*^−^*^1^ *dDiatN* = *dNH*4*up* + *dNO*3*up* − *dNH*4*out* − *dAutN* − *dDetN* − Σ *Pred* (B.38) gN m*^−^*^3^ d*^−^*^1^ *dDiatP* = *dPup* − *dPout* − *dAutP* − *dDetP* − Σ *Pred* (B.39) gP m*^−^*^3^ d*^−^*^1^

*dt*

*dt*

*dt*

*dDiatSi* = *dSiup* − *dAutSi* − *dDetSi* − Σ *Pred* (B.40) gSi m*^−^*^3^ d*^−^*^1^

*dt*

*dDiatChl* = *dChlsyn* − *dChldeg* − *dAutChl* − *dDetChl* − Σ *Pred* (B.41) gChl m*^−^*^3^ d*^−^*^1^

*dt*

#### Table B.8: Conservation equations for green algae SVs.

conservation equation unit

*dGreenC* = *dCfix* − *dCleak* − *dCvoid* − *dCresp* − *dAutC* − *dDetC* − Σ *Pred* (B.42) gC m*^−^*^3^ d*^−^*^1^ *dGreenN* = *dNH*4*up* + *dNO*3*up* − *dNH*4*out* − *dAutN* − *dDetN* − Σ *Pred* (B.43) gN m*^−^*^3^ d*^−^*^1^ *dGreenP* = *dPup* − *dPout* − *dAutP* − *dDetP* − Σ *Pred* (B.44) gP m*^−^*^3^ d*^−^*^1^

*dt*

*dt*

*dt*

*dGreenChl* = *dChlsyn* − *dChldeg* − *dAutChl* − *dDetChl* − Σ *Pred* (B.45) gChl m*^−^*^3^ d*^−^*^1^

*dt*

#### Table B.9: Conservation equations for protozooplankton SVs.

conservation equation unit

*dZooC* = *dCeat* − *dPOCout* − *dCresp* − *dAutC* − *dDetC* (B.46) gC m*^−^*^3^ d*^−^*^1^

*dt*

*dZooN* = *dNeat* − *dPONout* − *dAutN* − *dDetN* (B.47) gN m*^−^*^3^ d*^−^*^1^

*dt*

*dZooP* = *dPeat* − *dPOPout* − *dAutP* − *dDetP* (B.48) gP m*^−^*^3^ d*^−^*^1^

*dt*

#### Table B.10: Conservation equations for CM SVs.

conservation equation unit

*dCMC* = *dCfix* + *dCeat* − *dCleak* − *dCvoid* − *dPOCout* − *dCresp* − *dAutC* − *dDetC* − Σ *Pred*

*dt*

(B.49)

gC m*−*3 d*−*1

*dCMN* = *dNH*4*up* + *dNO*3*up* + *dNeat* − *dNH*4*out* − *dPONout* − *dAutN* − *dDetN* − Σ *Pred*

*dt*

(B.50)

gN m*−*3 d*−*1

*dCMP* = *dPup* + *dPeat* − *dPout* − *dPOPout* − *dAutP* − *dDetP* − Σ *Pred* (B.51) gP m*^−^*^3^ d*^−^*^1^

*dt*

*dCMChl* = *dChlsyn* − *dChldeg* − *dAutChl* − *dDetChl* − Σ *Pred* (B.52) gChl m*^−^*^3^ d*^−^*^1^

*dt*

#### Table B.11: Conservation equations for NCM SVs.

conservation equation unit

*dNCMC* = *dCfix* + *dCeat* − *dCleak* − *dCvoid* − *dPOCout* − *dCresp* − *dAutC* − *dDetC* − Σ *Pred*

*dt*

(B.53)

gC m*−*3 d*−*1

*dNCMN* = *dNeat* − *dPONout* − *dAutN* − *dDetN* − Σ *Pred* (B.54) gN m*^−^*^3^ d*^−^*^1^

*dt*

*dNCMP* = *dPeat* − *dPOPout* − *dAutP* − *dDetP* − Σ *Pred* (B.55) gP m*^−^*^3^ d*^−^*^1^

*dt*

*dNCMChl* = *dChlup* − *dChldeg* − *dAutChl* − *dDetChl* − Σ *Pred* (B.56) gChl m*^−^*^3^ d*^−^*^1^

*dt*

### 30 Appendix B.6. Model equations

*Appendix B.6.1. Mathematical equations*

*normalize*(*x, x*

*min*

*, xmax*

) = *x − x_min_*

*xmax − xmin*

(B.57)

*gompertz*(*L, b, x*) = *L · exp*(*−b · exp*(*−k · x*)) (B.58)

*R*

*monod*(*R, kt*) =

*R* + *kt*

(B.59)

Table B.12: List of all parameters for the mathematical functions listed above.

| **parameter** | **parameter description** | **unit** |
| --- | --- | --- |
| L | upper asymptote | dl |
| b | displacement along the x-axis | dl |
| k | growth rate of gompetz curve | dl |
| R | resource | dl |
| kt | hald-saturation constant | dl |

### Appendix B.6.2. Module cellular status

#### Table B.13: Summary of the auxiliaries in the module cellular status.

| auxiliary | description | unit | origin | eq. # |
| --- | --- | --- | --- | --- |
| *Nut_i_C* | cellular carbon quota for nitrogen, phosphate, silica and chlorophyll-a | gNut gC^−1^ | [Flynn (2001)](#_bookmark51) | [B.60,](#_bookmark2)  [B.61,](#_bookmark3) [B.62,B.63](#_bookmark5) |
| *UmT* | maximum possible growth rate at the  current temperature | d−1 | [Flynn (2021)](#_bookmark52) | [B.64](#_bookmark6) |
| *BR* | basal respiration at the current  temperature | d−1 | [Flynn (2001)](#_bookmark51) | [B.66](#_bookmark8) |
| *totR* | total respiration taking metabolic,  anabolic and foraging costs into account. | gC gC^−1^ d^−1^ | [Flynn (2021)](#_bookmark52) | [B.71](#_bookmark13) |
| *Cu* | net carbon specific growth rate taking  phagotrophic and phototrophic carbon sources into account | gC gC^−1^ d^−1^ | [Flynn (2021)](#_bookmark52) | [B.72](#_bookmark14) |
| *NCu* | cellular nitrogen status (1 = saturated;  0 = limited) determined using a linear relationship. | dl | modified  from [Flynn](#_bookmark52) [(2021)](#_bookmark52) | [B.68](#_bookmark10) |
| *PCu* | cellular phosphate status (1 = saturated;  0 = limited) determined using a Gompertz curve | dl | modified  from [Flynn](#_bookmark52) [(2021)](#_bookmark52) | [B.69](#_bookmark11) |
| *SCu* | cellular silica status (1 = saturated; 0 =  limited) | dl | [Flynn (2021)](#_bookmark52) | [B.70](#_bookmark12) |
| *DOCvoid* | voiding of DOC if minimum quota is  reached | gC gC^−1^ | [Flynn (2021)](#_bookmark52) | [B.67](#_bookmark9) |
| *mrt* | mortality rate | gC gC^−1^ | [Flynn (2021)](#_bookmark52) | [B.65](#_bookmark7) |

*NC* = *PC* = *SC* =

*protN protC protP protC protSi protC*

*protChl*

(B.60)

(B.61)

(B.62)

*ChlC* =

*protC*

*T emp−RT*

(B.63)

*UmT* = *UmRT · Q*10

10 (B.64)

*mrt* = *mrtRT · Q*10

*T emp RT*

10 (B.65)

*−*

*BR* = *UmT · CR* (B.66)

*protN*

*DOCvoid* = *NC < NCmin, protC − NCmin,* 0*.*0 (B.67)

*NCu* = *min*(1*.*0*, max*(0*.*0*, normalizeNC, NCmin, NCmax*)) (B.68)

*PCu* = *gompertz*(1*.*0*,* 6*.*0*,* 10*.*0*, normalize*(*PC, PCmin, PCmax*)) (B.69)

*SCopt*

*SCu* = *min*((*monod*(*Si, ktSi*) *· SCmin* )*,* 1*.*0) (B.70)

*totR* = (*redco · upNO*3) + *AR ·* (*upNH*4 + *upNO*3 + *assN · SDA*) + (*assC · SDA*) + *BR* (B.71)

*Cu* = *Cfix* + *assC − totR* (B.72)

### 35 Appendix B.6.3. Module uptake

#### Table B.14: Summary of the auxiliaries in the module uptake.

| auxiliary | description | unit | origin | eq. # |
| --- | --- | --- | --- | --- |
| *upP* | uptake of phosphate described using the monod function and enhanced or repressed using two logistic sigmoid  functions. | gP gC^−1^ d^−1^ | modified from [Flynn (2021)](#_bookmark52) | [B.73](#_bookmark15) |
| *upNH*4 | uptake of ammonium described using the monod function and enhanced or repressed using two logistic sigmoid  functions. | gN gC^−1^ d^−1^ | modified from [Flynn (2021)](#_bookmark52) | [B.74](#_bookmark16) |
| *upNO*3 | uptake of nitrite described using the  monod function and enhanced using a logistic sigmoid functions. | gN gC^−1^ d^−1^ | modified from [Flynn (2021)](#_bookmark52) | [B.75](#_bookmark17) |
| *upSi* | uptake of silica described using the monod function and enhanced using a  logistic sigmoid functions. | gSi gC^−1^ d^−1^ | modified from [Flynn (2021)](#_bookmark52) | [B.76](#_bookmark18) |

P uptake

*APin_P_* = *logistic*(1*.*0*, −*16*.*0*,* 0*.*7*, normalize*(*PC, PCmin, PCopt*)) *APde_P_* = *logistic*(1*.*0*, −*40*.*0*,* 0*.*9*, normalize*(*PC, PCmin, PCmax*)) *upP_opt_* = *monod*(*P, ktP* ) *· UmT · PCopt*

*upP* = *upP_opt_ · APin_P_ ·* 10*.*0 + *upP_opt_ · APde_P_* (B.73)

NH^+^ uptake

4

*NCPopt* = ((*PCu < NCu*)*, PCoNCop* + *PCu ·* (*NC − PCoNCop*)*, NC*)

*APin_NH_*_4_ = *logistic*(1*.*0*, −*24*.*0*,* 0*.*85*, normalize*(*NC, NCmin, NCPopt*)) *NCPopt* = ((*PCu < NCu*)*, PCoNCm* + *PCu ·* (*NC − PCoNCm*)*, NC*)

*APde_P_* = *logistic*(1*.*0*, −*40*.*0*,* 0*.*85*, normalize*(*NC, NCmin, NCPmax*)) *upNH*4*_opt_* = *monod*(*NH*4*, ktNH*4) *· UmT · NCopt · relUm_NH_*_4_

*upNH*4 = *upNH*4*_opt_ · APin_NH_*_4_ *·* 3*.*0 + *upNH*4*_opt_ · APde_NH_*_4_ (B.74)

NO^−^_3_ uptake

*NCPm* = ((*PCu < NCu*)*, PCoNCm* + *PCu ·* (*NC − PCoNCm*)*, NC*)

*APde_NO_*_3_ = *logistic*(1*.*0*, −*55*.*0*,* 0*.*9*, normalize*(*NC, NCmin, NCPm*)) *upNO*3*_opt_* = *monod*(*NO*3*, ktNO*3) *· UmT · NCopt · relUm_NO_*_3_

*upNO*3 = *upNO*3*_opt_ · APde_NO_*_3_ (B.75)

Si uptake

*APde_Si_* = *logistic*(1*.*0*, −*80*.*0*,* 0*.*95*, normalize*(*SC, SCmin, SCmax*)) *upSi_opt_* = *monod*(*Si, ktSi*) *· UmT · SCopt*

*upSi* = *upSi_opt_ · APde_Si_* (B.76)

### Appendix B.6.4. Module phototrophy

#### Table B.15: Summary of the auxiliaries in the module phototrophy.

| auxiliary | description | unit | origin | eq. # |
| --- | --- | --- | --- | --- |
| *PSqm* | maximal attainable photosynthetic rate  under optimum light (plateau of the PE-curve) | gC gC^−1^ d^−1^ | [Flynn (2001)](#_bookmark51) | [B.77](#_bookmark19) |
| *PS* | carbon fixation through photosynthesis at current light and current cellular  status | gC gC^−1^ d^−1^ | [Flynn (2001)](#_bookmark51) | [B.78](#_bookmark20) |
| *Cfix* | net carbon fixation taking leakage into  account | gC gC^−1^ d^−1^ | [Flynn (2001)](#_bookmark51) | [B.79](#_bookmark21) |
| *synChl* | synthesis of chlorophyll-a | gChl gC d^−1^ | modified  from [Flynn](#_bookmark52) [(2021)](#_bookmark52) | [B.80](#_bookmark22) |
| *degChl* | degradation of chlorophyll-a | gChl gC^−1^ d^−1^ | [Flynn (2021)](#_bookmark52) | [B.81](#_bookmark23) |
| *degChl_NCM_* | loss of chlorophyll-a | gChl gC^−1^ d^−1^ | [Ghyoot et al.](#_bookmark57)  [(2017)](#_bookmark57) | [B.82](#_bookmark24) |
| *upChl* | uptake of chlorophyll-a from prey | gChl gC^−1^ d^−1^ | modified  from [Ghyoot](#_bookmark57) [et al. (2017)](#_bookmark57) | [B.83](#_bookmark25) |

*PSqm* = [*UmT · relPS ·* (1 + *PSDOC*) + *NCm · UmT ·* (*redco* + *AR*)] *· NCu* + *BR* (B.77)

*α^Chl^ · ChlC · PFD ·* 24*.*0 *·* 60*.*0 *·* 60*.*0

*X* =

*PS* =

*PSqm*

*PSqm ·* (*log*(*X* + *sqrt*(1*.*0 + *X*^2^)) *− log*(*X · exat* + *sqrt*(1*.*0 + (*X · exat*)^2^))) *atten*

(B.78)

*Cfix* = *PS ·* (1*.*0 *− PSDOC*) (B.79)

*Cfix synChl* = *ChlCmax · UmT · NPSiCu · M ·* (1*.*0 *− PSqm* )*·*

*logistic*(0*.*95*, −*24*.*0*,* 0*.*85*, normalize*(*ChlC, ChlCmin, ChlCmax*)) (B.80)

*degChl* = (*min*(*ChlC, ChlCmax*) *· UmT ·* (1*.*0 *− NPSiCu*)) (B.81)

*degChl_NCM_* = *constant* (B.82)

*upChl* = *logistic*(1*.*0*, −*80*,* 0*.*93*, normalize*(*ChlC,* 0*.*0*, ChlCmax*)) (B.83)

### Appendix B.6.5. Module phagotrophy

#### Table B.16: Summary of the auxiliaries in the module phagotrophy.

| auxiliary | description | unit | origin | eq. # |
| --- | --- | --- | --- | --- |
| *mot* | motility of the protists | m s-1 | [Flynn & Mitra](#_bookmark54)  [(2016)](#_bookmark54) | [B.84](#_bookmark26) |
| *nrPrey* | density of prey in segment | nr cells m^−3^ | modified from  [Flynn (2021)](#_bookmark52) | [B.85](#_bookmark27) |
| *enc* | encounter rate | prey  predator- 1 d−1 | [Rothschild &](#_bookmark62) [Osborn (1988)](#_bookmark62) | [B.86](#_bookmark28) |
| *capPrey* | potential C-specific capture of prey | gC gC^−1^ d^−1^ | [Flynn (2021)](#_bookmark52) | [B.87](#_bookmark29) |
| *sumCP* | captured prey | gC gC^−1^ d-1 | [Flynn (2021)](#_bookmark52) | [B.88](#_bookmark30) |
| *opAE* | assimilation efficiency | dl | [Flynn (2021)](#_bookmark52) | [B.92](#_bookmark31) |
| *maxIng* | maximum ingestion rate | gC gC^−1^ d-1 | [Flynn (2021)](#_bookmark52) | [B.93](#_bookmark32) |
| *satIng* | saturation ingestion rate | gC gC^−1^ d^−1^ | [Flynn (2021)](#_bookmark52) | [B.94](#_bookmark33) |
| *ingC* | actual carbon ingestion rate | gC gC^−1^ d^−1^ | [Flynn (2021)](#_bookmark52) | [B.95](#_bookmark34) |
| *ingNut_i_* | nutrient ingestion rate | gNut gC^−1^ d−1 | [Flynn (2021)](#_bookmark52) | [B.96,](#_bookmark35)  [B.97](#_bookmark36) |
| *assC* | carbon assimilation rate | gC gC^−1^ d^−1^ | [Flynn (2021)](#_bookmark52) | [B.98](#_bookmark37) |
| *assNut_i_* | nutrient assimilation rate | gNut gC^−1^ d−1 | [Flynn (2021)](#_bookmark52) | [B.99,](#_bookmark38)  [B.100](#_bookmark39) |

*mot* = 1*e*^−6^ *·* (38*.*542 *·* (*r ·* 2)^0^*^.^*^5424^) (B.84)

*lightInh* = *sigmoidLogistic*((1 *− relPhag*)*,* 10*.*0*,* 1*.*0*, PFD*) + (1*.*0 *−* (1 *− relPhag*))

*preyC*

*nrPrey* = *lightInh ·* 1*e*12 *· CcellPrey* (B.85)

*encPrey* = (24*.*0 *·* 60*.*0 *·* 60*.*0) *· π ·* ( *rPrey* + *rProt* )^2^ *· nrPrey*

1*E*6 1*E*6

2 2 2 2 −0*.*5 −1*.*0

*·* ((*vel* + 3 *· vel* + 4 *· wTurb* ) *·* ((*vel* + *wTurb* ) )) *·* 3*.*0 (B.86)

2

*prey*

*pred*

*pred*

*CcellPrey*

*capPrey* = *encPrey · PR · optCR · CcellPred* (B.87)

*sumCP* = *sum*(*capPrey*) (B.88)

*capPrey preyN*

*ingNC* =

*sumCP · preyC* (B.89)

*capPrey preyP*

*ingPC* =

*sumCP · preyC* (B.90)

*stoichP* = *min*(

*ingNC*

*,*

*NCopt*

*ingPC PCopt*

*,* 1*.*0) (B.91)

*opAE* = (*AEo* + (*AEm − AEo*) *· monod*(*stoichP, kAE*) *·* (1*.*0 + *kAE*)) *· stoichP* (B.92)

*UmT* + *BR* 1

*maxIng* =

- 1. *− SDA · opAE opAE* (B.93)

*maxIng*

| *satIng* = *maxIng · monod*(*sumCP,* | 4 | ) (B.94) |
| --- | --- | --- |
| *ingC* = *min*(*ingSat, sumCP* ) |  | (B.95) |
| *ingN* = *ingC · ingNC ingP* = *ingC · ingPC assC* = *ingC · opAE assN* = *assC · NCopt*  *assP* = *assC · PCopt* |  | (B.96)  (B.97)  (B.98)  (B.99)  (B.100) |

# Appendix C. Boundary forcings


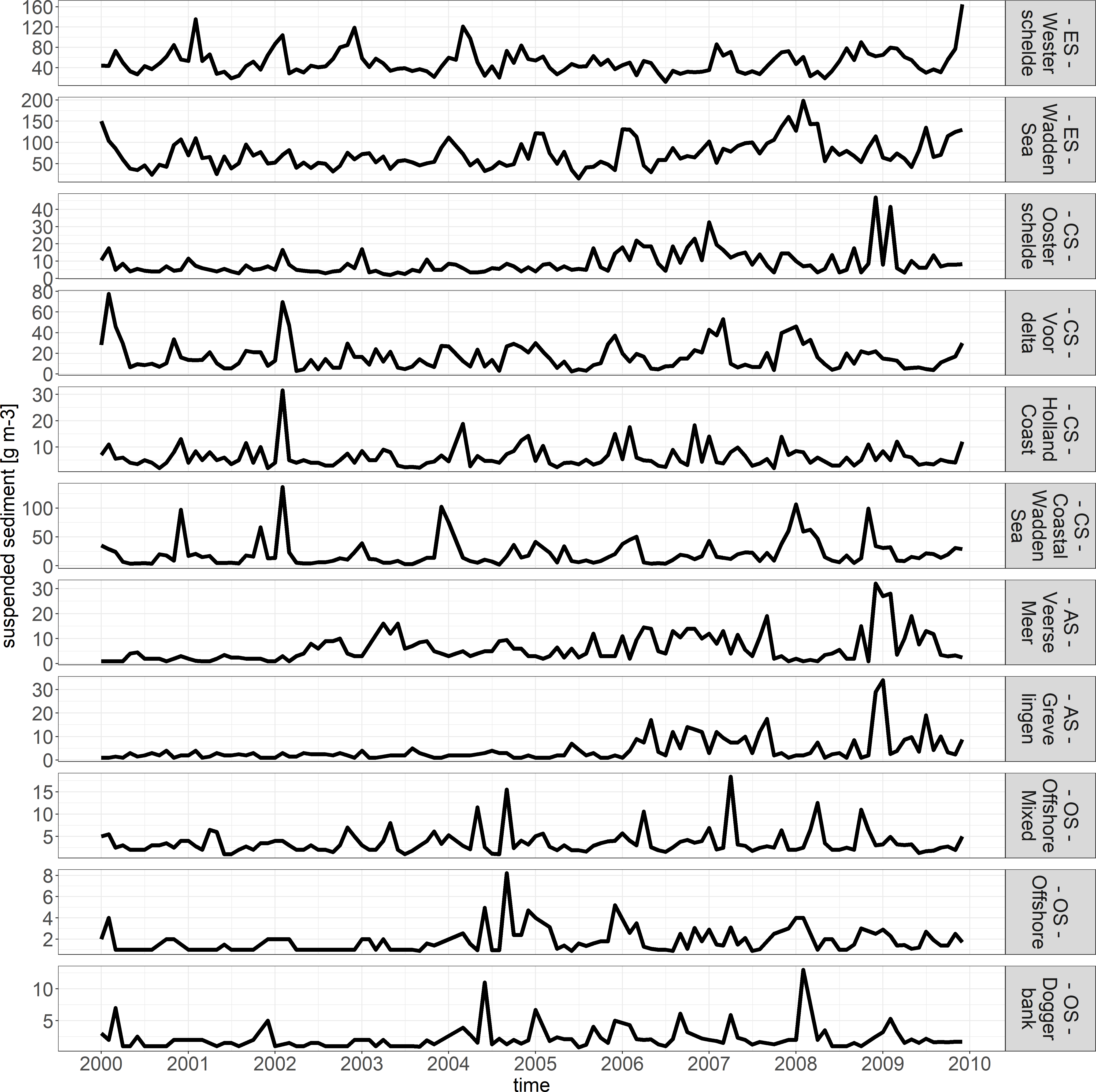


#### Figure C.1: Boundary transport of suspended sediment.


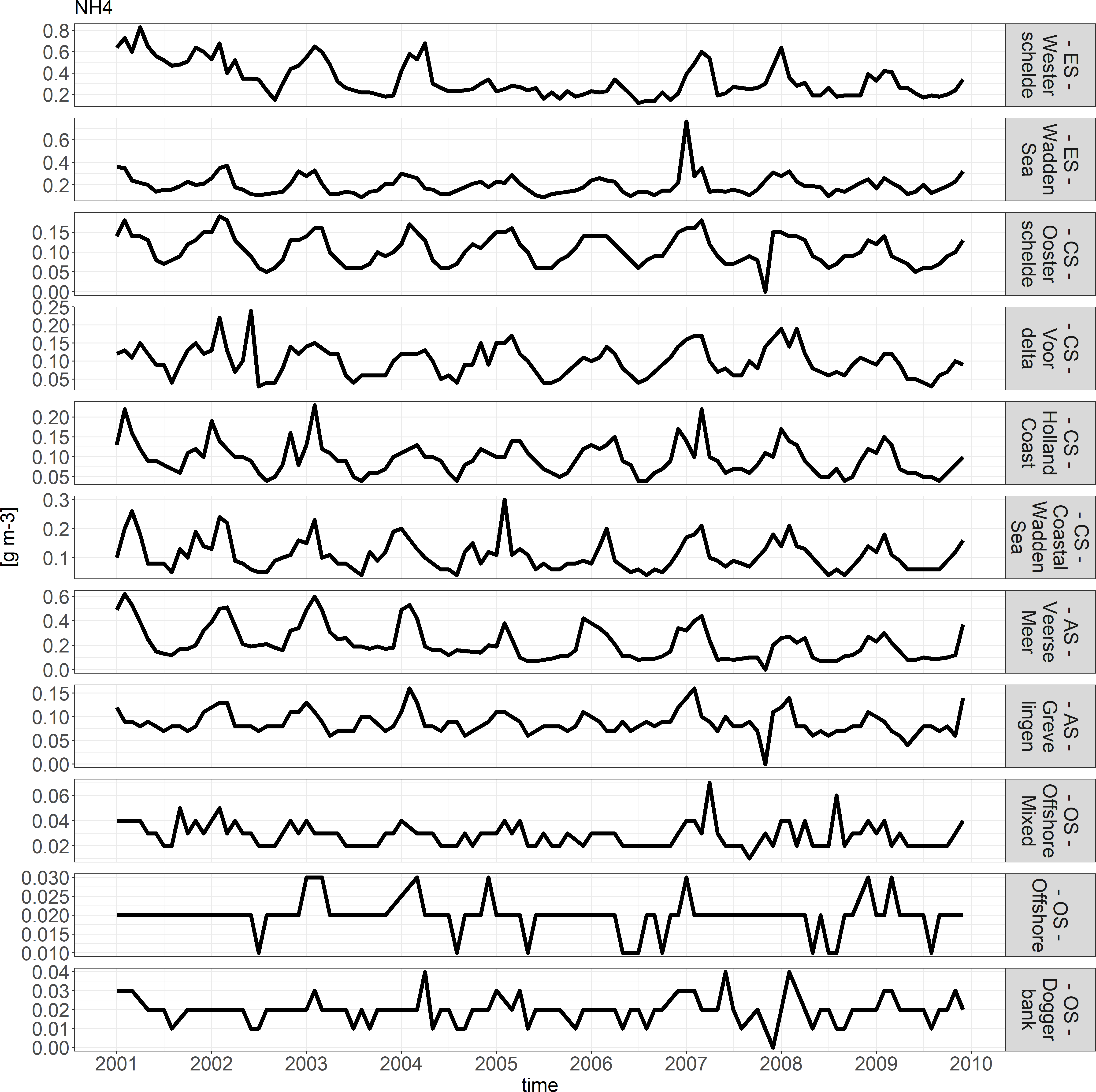


Figure C.2: Boundary transport of ammonium.


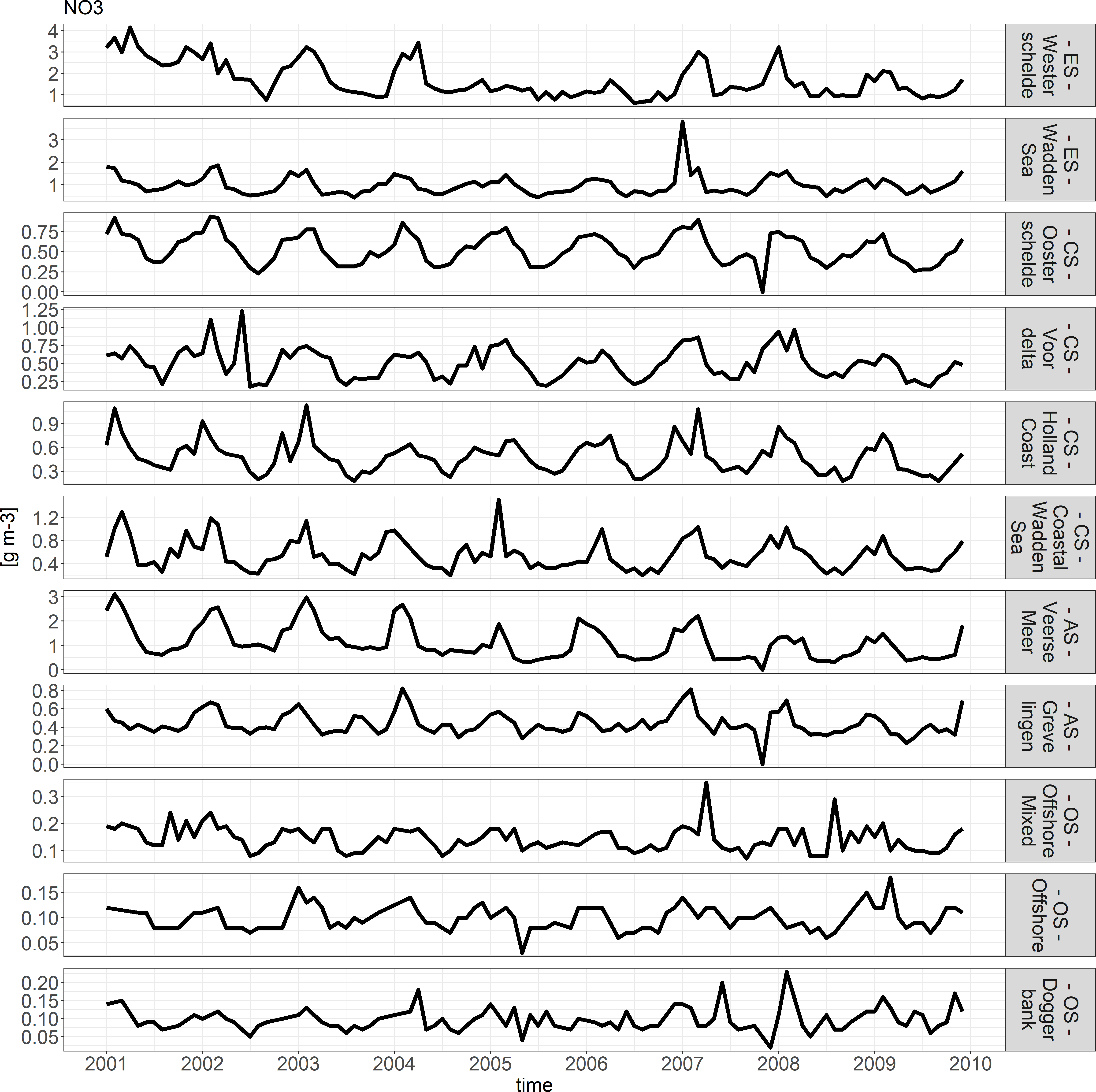


#### Figure C.3: Boundary transport of nitrate.


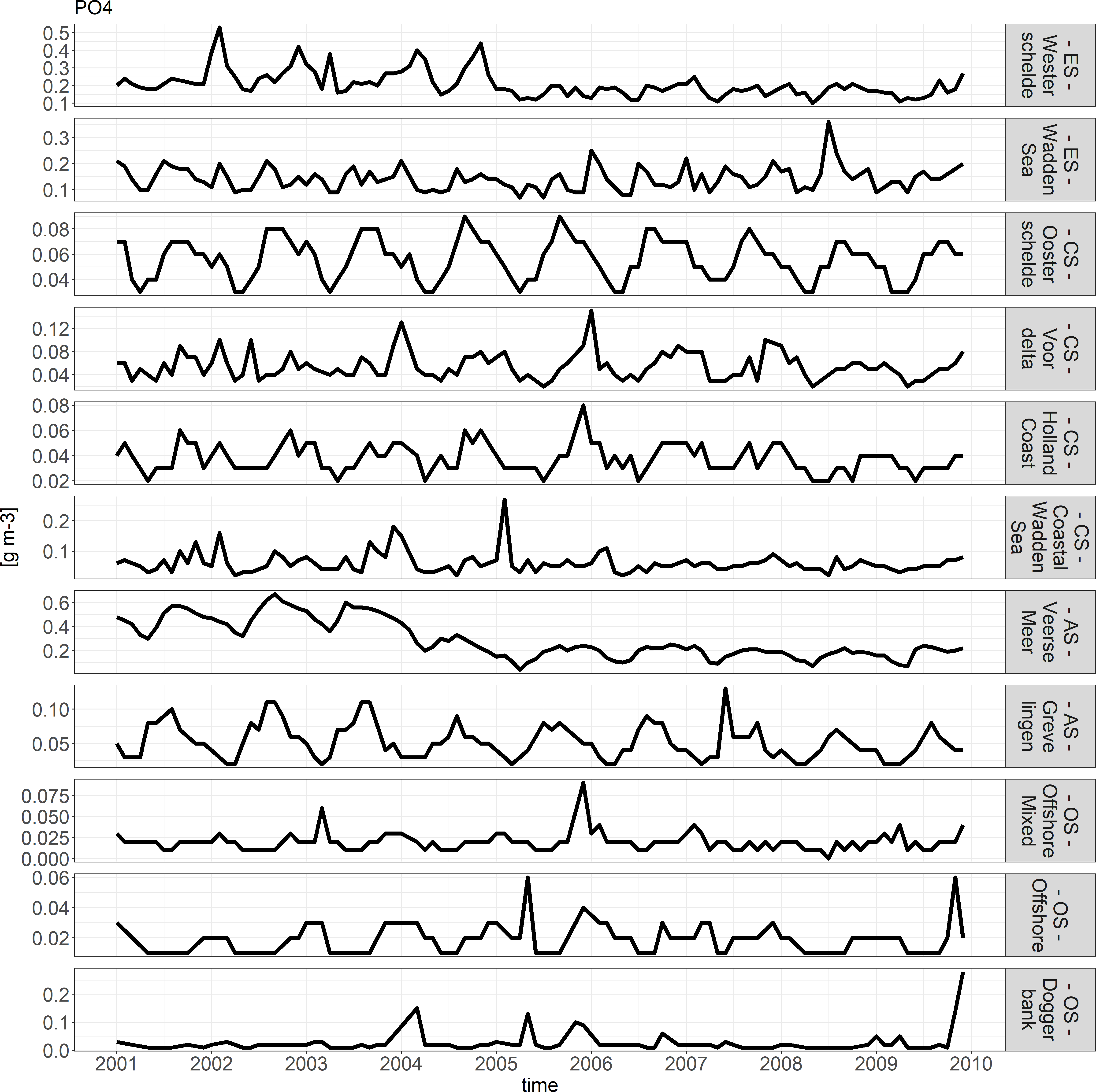


Figure C.4: Boundary transport of phosphorus.


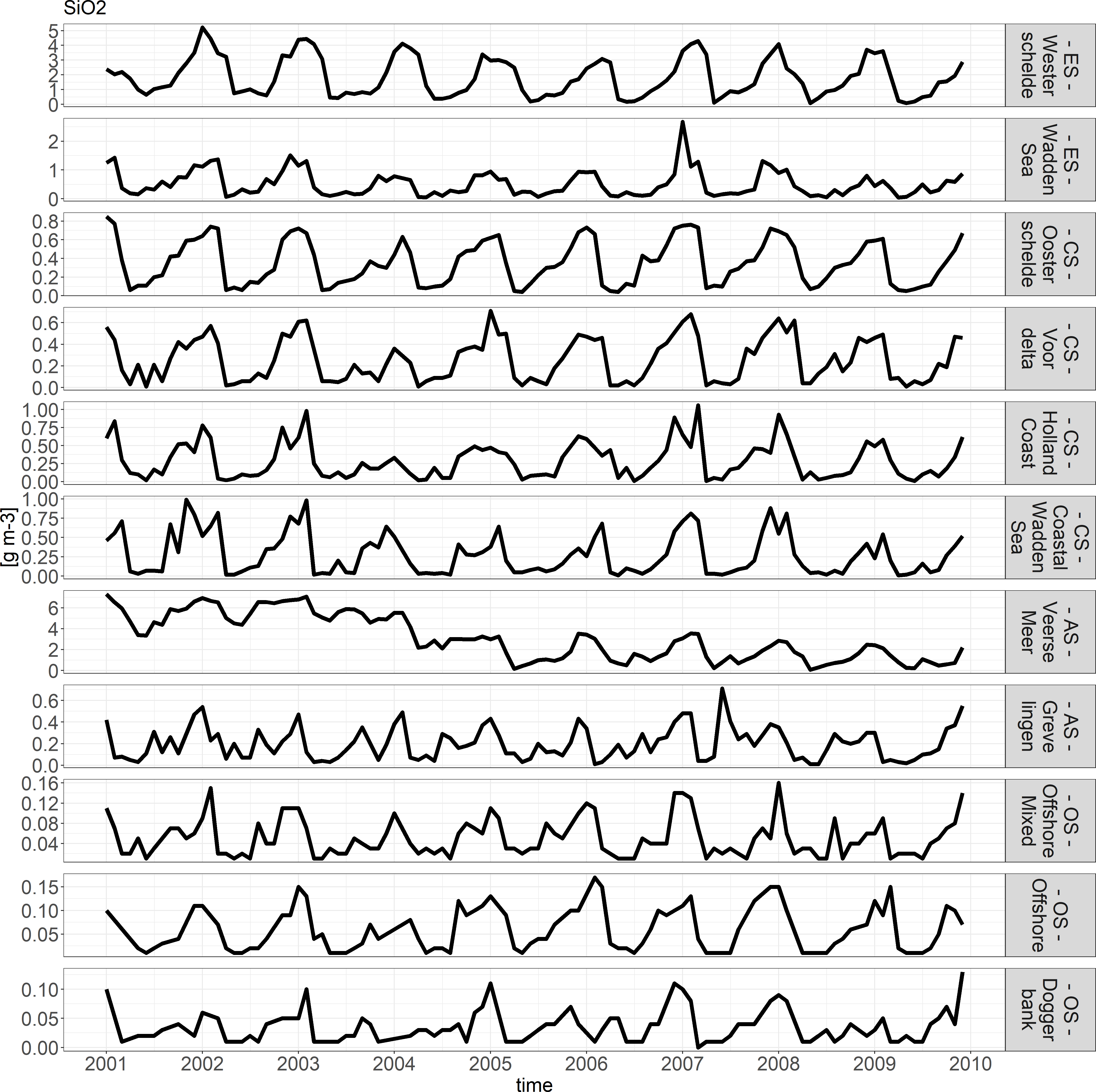


Figure C.5: Boundary transport of silica.

# Appendix D. Model forcings


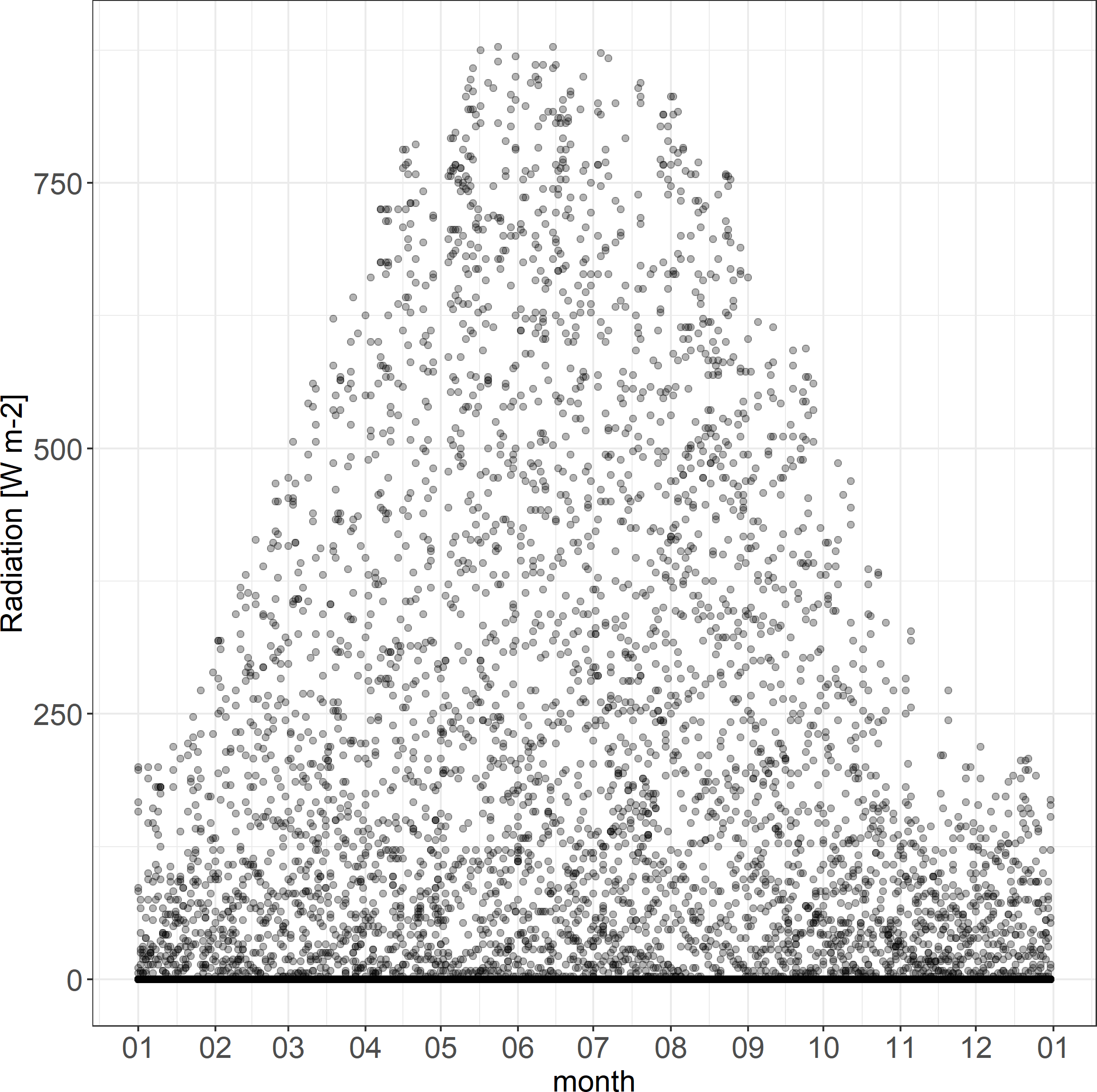


#### Figure D.6: Forced hourly radiation. Data was retrieved from the Royal Netherlands Meterorological Institute (KNMI) for the year 2019 for the sampling station de Kooy.


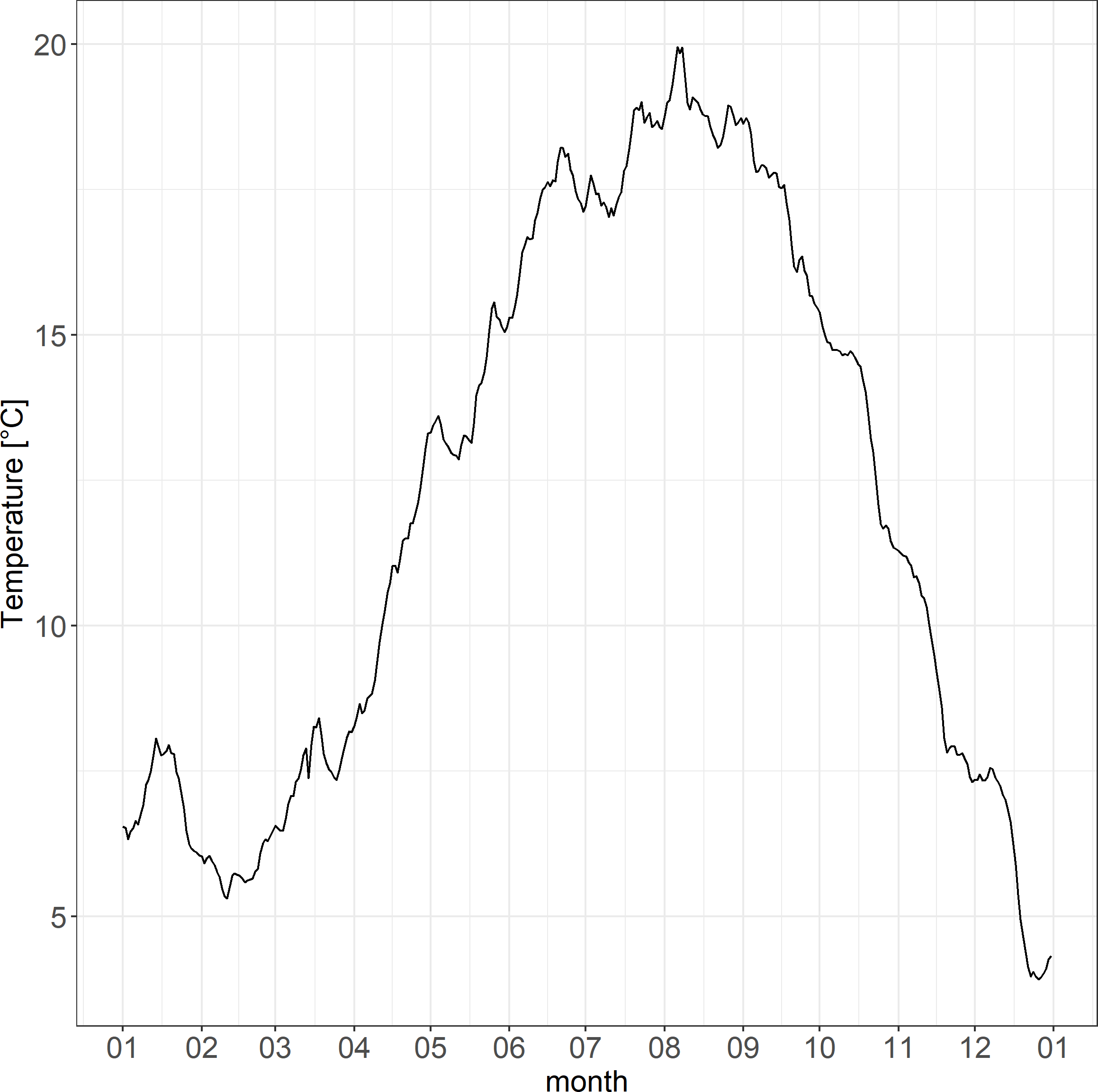


Figure D.7: Forced daily temperature. Data was retrieved from the 3D model Merzandwinning for the year 2014.

40 **Appendix E. Box model**

*Appendix E.1. Box model set-up*

The box model is used to demonstrate growth and competition between the five PFTs, diatoms, green algae, protozooplankton, CMs and NCMs. Only the PROTIST module was activated for the box model. It was run for 60 days with a timestep of 3 min and an output timestep of 2 h. The box model set-up mimics a

45 batch culture with an initial nutrient supply, a day-night cycle of 12:8 h, no remineralization of particulate organics and no additional mortality apart from grazing.

All PFTs had a growth rate of 0.81 d^−1^. Mortality was deactivated. The dimensionless parameter

*relPS* (the ratio of photosynthesis rate to maximum growth rate) was set to 2 for the primarily phototrophic organisms and to 0.5 for NCMs. [Stoecker et al.](#_bookmark65) [(1988)](#_bookmark65) showed that NCMs ingest less prey in the dark, so

50 the ingestion of prey by NCMs is slightly light dependent (0.7). As there were no NCMs present in that dataset, the size for NCMs was set to 40 *µ*m ESD to mimic an average *Strombidium*. The parameters for the other PFTs were set according to the table in [B.2.](#_bookmark0)

### Appendix E.2. Box model results

Figure [E.8](#_bookmark40) displays a 60 days run of the box model mimicking a batch culture. It displays the carbon

55 biomass SVs (fig. [E.8a),](#_bookmark41) the nutrient SVs (figs. [E.8b,](#_bookmark42) [E.8d,](#_bookmark43) [E.8f)](#_bookmark45) as well as the assimilation rates (fig. [E.8c)](#_bookmark44) and carbon fixation rates (fig. [E.8e).](#_bookmark46) These plots demonstrate that PROTIST responds as would be expected.

The primarily phototrophic organisms bloom first with diatoms displaying the highest biomass peak (see fig. [E.8a).](#_bookmark41) All primarily phototrophic organisms initially display high rates of carbon fixation, which

60 respond to the day-night cycle (see fig. [E.8e),](#_bookmark46) but those carbon fixation rates decline as the macro nutrients become limiting. Macronutrients become limiting after approximately 15 days (see figs. [E.8b,](#_bookmark42) [E.8d,](#_bookmark43) [E.8f](#_bookmark45)) leading to a decline of the diatoms and green algae. As the diatoms remain silica limited (see fig. [E.8f),](#_bookmark45) their biomass as well as carbon fixation rates remain low compared to green algae and CMs which display an increase of biomass as ammonium and phosphate become available again through voiding.

65 Fig. [E.8c](#_bookmark44) shows that all organisms capable of phagotrophy are prey limited as their assimilation rates closely follow their preys’ biomass curves. The assimilation of prey by NCMs is also reflected in their carbon fixation rates, which are initially very low but increase as the NCM assimilated prey and retains their chloroplasts (see fig. [E.8e).](#_bookmark46)

- - 1. (b)


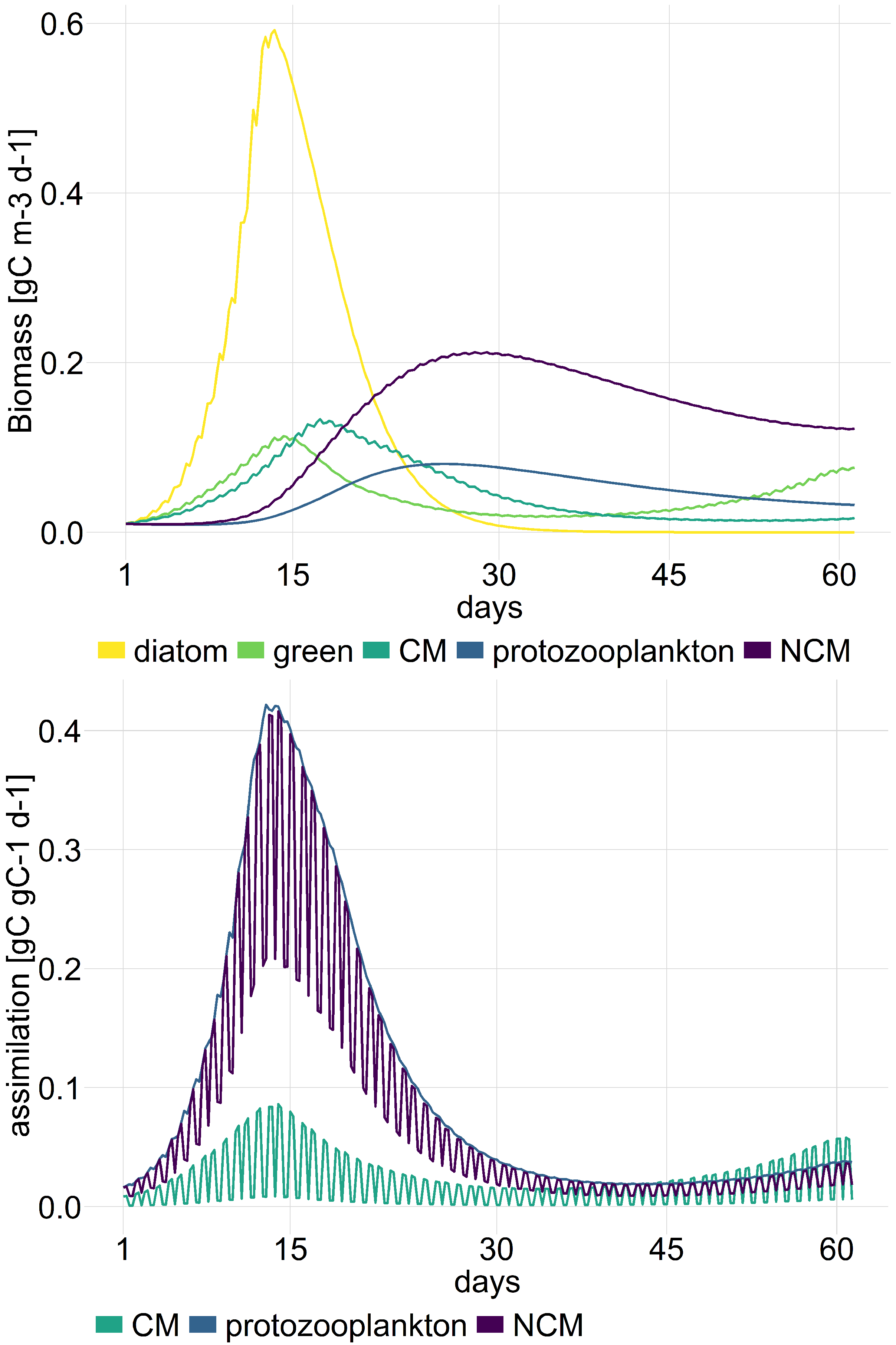

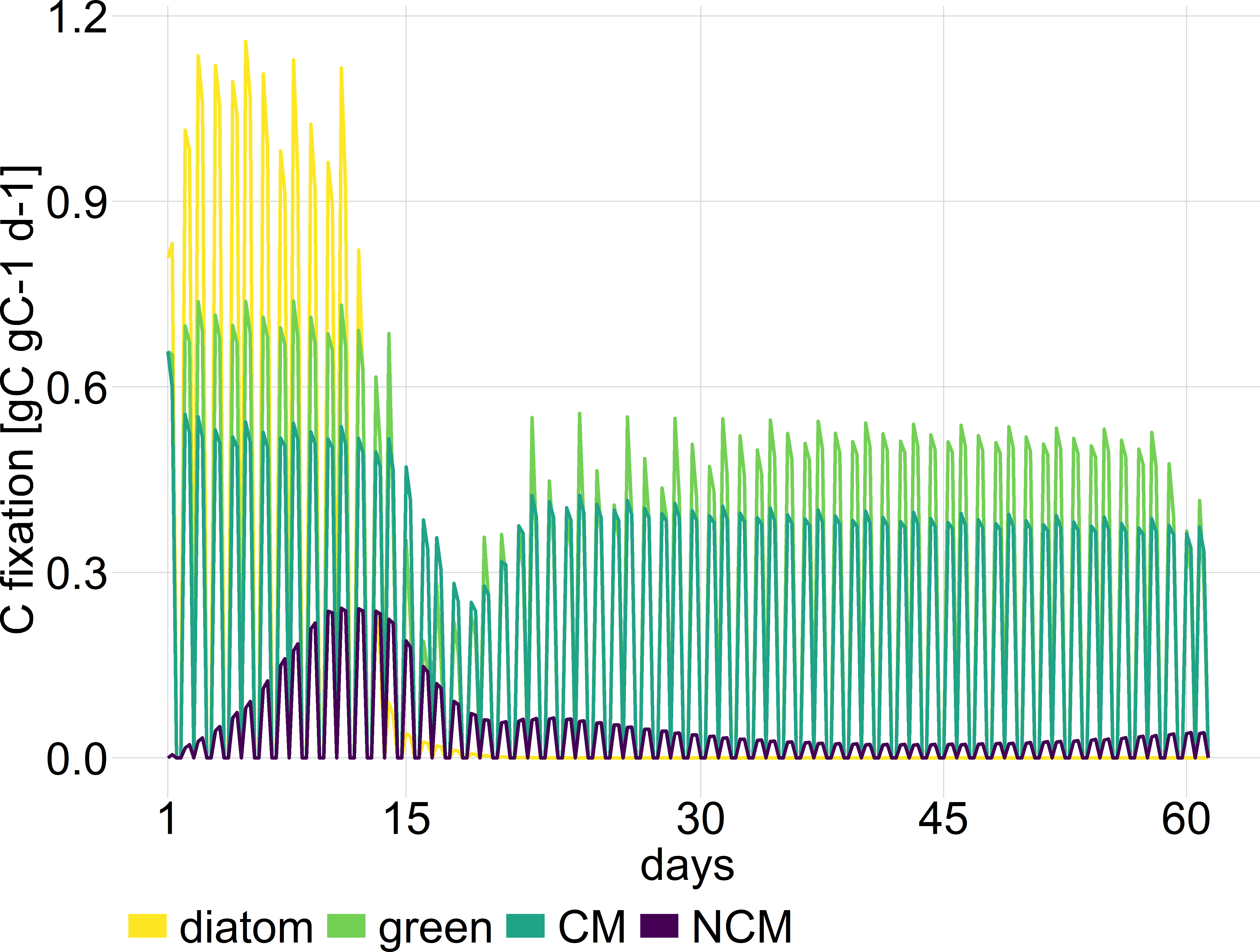

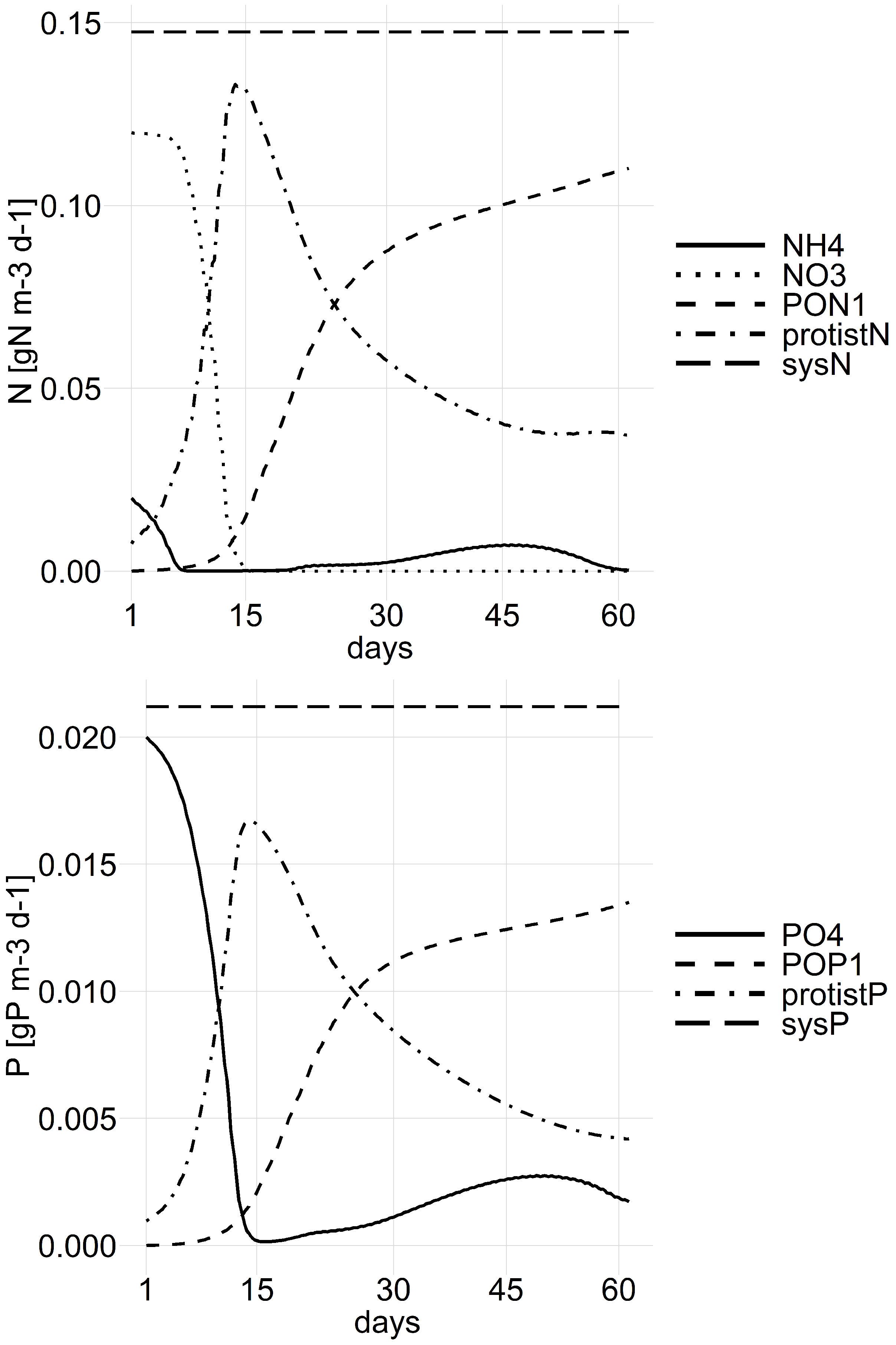

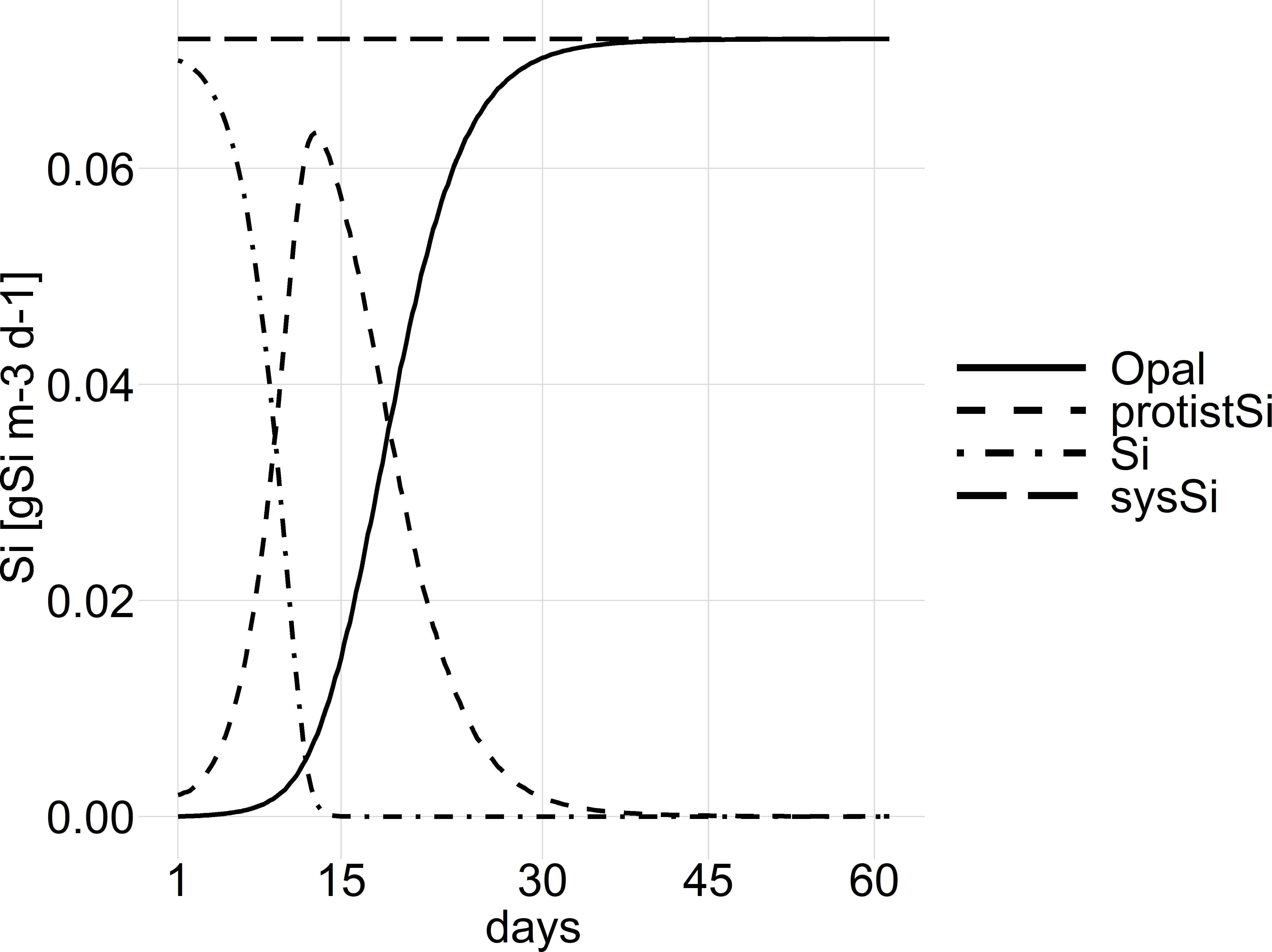


(c) (d)

(e) (f)

Figure E.8: Graphs displaying a) the carbon biomass per PFT, b) all SV related to nitrogen, c) assimilation of prey, d) all SV related to phosphate, e) carbon fixation and f) all SV related to silica.

# Appendix F. Normalized standard deviation

Table F.17: Normalized standard deviations of the abiotic factor included in the sensitivity analysis.

NH^+^

4

*sd_x_*

## 0.95

NO^−^3 0.95

PO3−

4

## 1.24

SiO_2_ 1.78

## suspended sediment 1.46

70 **References**

Adolf, J. E., Stoecker, D. K., & Harding, L. W. (2006). The balance of autotrophy and heterotrophy during mixotrophic growth of Karlodinium micrum (Dinophyceae). *Journal of Plankton Research*, *28* , 737–751. doi:[10.1093/plankt/fbl007](http://dx.doi.org/10.1093/plankt/fbl007).

Anderson, R., Charvet, S., & Hansen, P. J. (2018). Mixotrophy in Chlorophytes and Haptophytes—Effect of

75 Irradiance, Macronutrient, Micronutrient and Vitamin Limitation. *Frontiers in Microbiology* , *9* . doi:[10.](http://dx.doi.org/10.3389/fmicb.2018.01704) [3389/fmicb.2018.01704](http://dx.doi.org/10.3389/fmicb.2018.01704).

Blauw, A. N., Los, H. F. J., Bokhorst, M., & Erftemeijer, P. L. A. (2009). GEM: A generic ecological model for estuaries and coastal waters. *Hydrobiologia*, *618* , 175–198. doi:[10.1007/s10750-008-9575-x](http://dx.doi.org/10.1007/s10750-008-9575-x).

Deltares (2021). D-Water Quality, Versatile water quality modelling. Available at:

80 https://content.oss.deltares.nl/delft3d/manuals/D-Water Quality Processes Technical Reference Manual.pdf.

Flynn, K. J. (2001). A mechnistic model for describing dynamic multi-nutrient, light, temperature interactions in phytoplankton. *Journal of Plankton Research*, *23* , 977–997. doi:https://doi.org/10. 1093/plankt/23.9.977.

Flynn, K. J. (2021). *Enhancing Microalgal Production - constructing decision support tools using system*

85 *dynamics modelling*. Zenodo. doi:[http://doi.org/10.5281/zenodo.5036605.](http://doi.org/10.5281/zenodo.5036605)

Flynn, K. J., & Mitra, A. (2009). Building the ”perfect beast”: modelling mixotrophic plankton. *Journal of Plankton Research*, *31* , 965–992. doi:[10.1093/plankt/fbp044](http://dx.doi.org/10.1093/plankt/fbp044).

Flynn, K. J., & Mitra, A. (2016). Why Plankton Modelers Should Reconsider Using Rectangular Hyperbolic (Michaelis-Menten, Monod) Descriptions of Predator-Prey Interactions. *Frontiers in Marine Science*, *3* .

90 doi:[10.3389/fmars.2016.00165](http://dx.doi.org/10.3389/fmars.2016.00165).

Geider, R. J., MacIntyre, H. L., & Kana, T. M. (1997). Dynamic model of phytoplankton growth and acclimation: Responses of the balanced growth rate and the chlorophyll a:carbon ratio to light, nutrient- limitation and temperature. *Marine Ecology Progress Series*, *148* , 187–200. doi:[10.3354/meps148187](http://dx.doi.org/10.3354/meps148187).

Geider, R. J., MacIntyre, H. L., & Kana, T. M. (1998). A dynamic regulatory model of phytoplanktonic

95 acclimation to light, nutrients, and temperature. *Limnology and Oceanography* , *43* , 679–694. doi:[10.](http://dx.doi.org/10.4319/lo.1998.43.4.0679) [4319/lo.1998.43.4.0679](http://dx.doi.org/10.4319/lo.1998.43.4.0679).

Ghyoot, C., Flynn, K. J., Mitra, A., Lancelot, C., & Gypens, N. (2017). Modeling Plankton Mixotrophy: A Mechanistic Model Consistent with the Shuter-Type Biochemical Approach. *Frontiers in Ecology and Evolution*, *5* . doi:[10.3389/fevo.2017.00078](http://dx.doi.org/10.3389/fevo.2017.00078).

100

105

Jeong, H. J., du Yoo, Y., Kim, J. S., Seong, K. A., Kang, N. S., & Kim, T. H. (2010). Growth, feeding and ecological roles of the mixotrophic and heterotrophic dinoflagellates in marine planktonic food webs. *Ocean Science Journal* , *45* , 65–91. doi:[10.1007/s12601-010-0007-2](http://dx.doi.org/10.1007/s12601-010-0007-2).

Leonardos, N., & Geider, R. J. (2004). Effects of nitrate: Phosphate supply ratio and irradiance on the C:N:P stoichiometry of Chaetoceros muelleri. *European Journal of Phycology* , *39* , 173–180. doi:[10.1080/](http://dx.doi.org/10.1080/0967026042000201867) [0967026042000201867](http://dx.doi.org/10.1080/0967026042000201867).

Li, A., Stoecker, D. K., & Adolf, J. E. (1999). Feeding, pigmentation, photosynthesis and growth of the mixotrophic dinoflagelatte Gyrodinium galatheanum. *Aquatic Microbial Ecology* , *19* , 163–176. doi:doi: 10.3354/ame019163.

Menden-Deuer, S., & Lessard, E. J. (2000). Carbon to volume relationships for dinoflagellates, diatoms, and

110 other protist plankton. *Limnology and Oceanography* , *45* , 569–579. doi:[10.4319/lo.2000.45.3.0569](http://dx.doi.org/10.4319/lo.2000.45.3.0569).

Rothschild, B., & Osborn, T. (1988). Small-scale turbulence and plankton contact rates. *Journal of Plankton Research*, *10* . doi:https://doi.org/10.1093/plankt/10.3.465.

Schneider, L. K., Flynn, K., Herman, P. M., Troost, T. A., & Stolte, W. (2020). Exploring the trophic spectrum: placing mixoplankton into plankton community assessments of the Southern North Sea.

115 *Frontiers in Marine Science*, *7* . doi:[10.3389/fmars.2020.586915](http://dx.doi.org/10.3389/fmars.2020.586915).

Skovgaard, A. (1996). Mixotrophy in Fragilidium subglobosum (Dinophyceae): Growth and grazing responses as functions of light intensity. *Marine Ecology Progress Series*, *143* , 247–253. doi:[10.3354/meps143247](http://dx.doi.org/10.3354/meps143247).

Stoecker, D. K., Silver, M. W., Michaels, A. E., & Davis, L. H. (1988). Obligate mixotrophy in Laboea strobila, a ciliate which retains chloroplasts. *Marine Biology*, *99* , 415–423. doi:[10.1007/BF02112135](http://dx.doi.org/10.1007/BF02112135).
